# Synthesis of the tetracyclic skeleton of *Aspidosperma* alkaloids via PET-initiated cationic radical-derived interrupted [2 + 2]/*retro*-Mannich reaction

**DOI:** 10.3762/bjoc.21.189

**Published:** 2025-11-10

**Authors:** Ru-Dong Liu, Jian-Yu Long, Zhi-Lin Song, Zhen Yang, Zhong-Chao Zhang

**Affiliations:** 1 Laboratory of Chemical Genomics, School of Chemical Biology and Biotechnology, Peking University Shenzhen Graduate School, Shenzhen 518055, China; 2 Key Laboratory of Bioorganic Chemistry and Molecular Engineering of Ministry of Education and Beijing National Laboratory for Molecular Science, and Peking-Tsinghua Centre for Life Sciences, Peking University, Beijing 100871, China; 3 Shenzhen Bay Laboratory, Shenzhen 518055, China; 4 Key Laboratory of Structure-Based Drug Design & Discovery of Ministry of Education, Shenyang Pharmaceutical University, Liaoning Shenyang 110016, China

**Keywords:** *Aspidosperma* alkaloids, [2 + 2]-cycloaddition/*retro*-Mannich reaction, DFT study, photoinduced electron transfer

## Abstract

Natural products with topologically complex architectures are important sources in drug discovery. The pursuit of conciseness and efficiency in the total synthesis of natural products promotes continuous innovation and the development of new reactions and strategies. In this work, a PET-initiated cationic radical-derived interrupted [2 + 2]/*retro*-Mannich reaction of N-substituted cyclobutenone provided a facile approach to the direct construction of the ABCE tetracyclic framework of *Aspidosperma* alkaloids. DFT calculations showed that the rate-determining step of the key PET reaction involved C19–C12 bond formation and C19–C3 bond cleavage. Investigation of the bond length changes along the IRC path, spin density, and NBO analysis indicated that this process is neither strictly concerted nor stepwise, but falls in between, and involves a formal 1,3-C shift.

## Introduction

Photochemical reactions, which enable the construction of topologically complex architectures from simple building blocks, have attracted considerable attention in recent decades. Numerous approaches to natural product synthesis have been reported in which a photochemical transformation represents a key step [1–3]. In this context, the photochemical [2 + 2] cycloaddition and subsequent fragmentation of the resulting cyclobutane provides a valuable strategy for synthesizing natural and unnatural products from simple building blocks [4]. Three distinct photoinitiated approaches have been established for the formation of the [2 + 2] cycloadducts: direct irradiation [5–6], energy transfer (EnT) [7], and photoinduced electron transfer (PET, or photoredox catalysis) processes [8–10].

**Figure 1:**
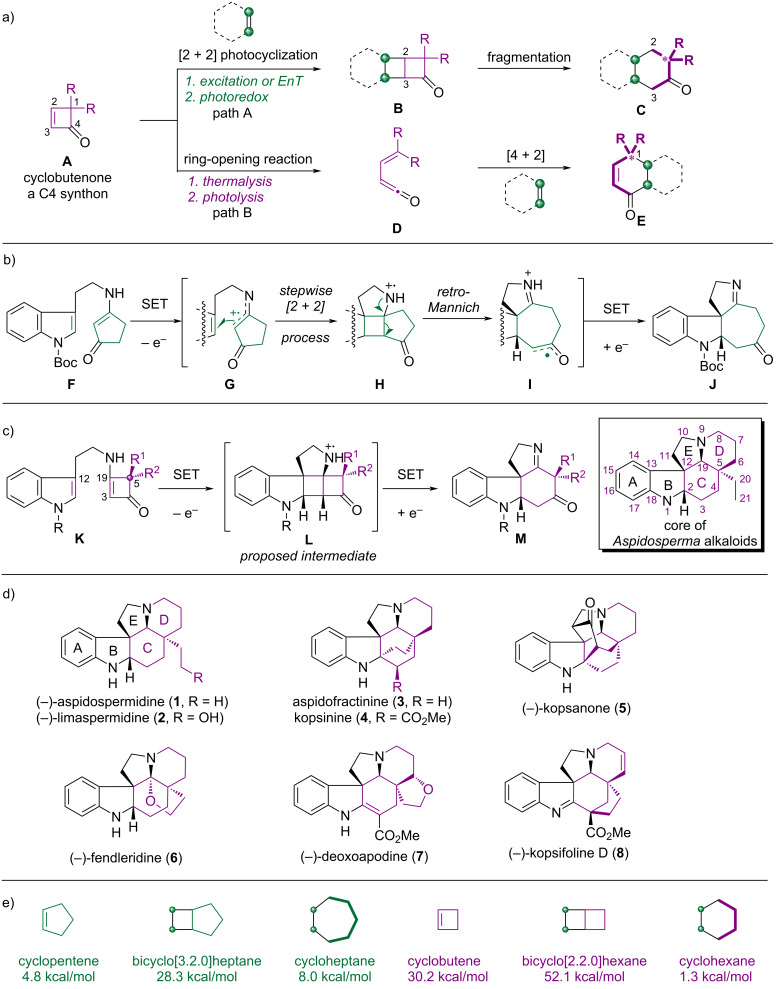
Synthetic plan. a) General model of cyclobutenone bond cleavage; b) our previously reported method; c) plan for indole *Aspidosperma* alkaloid synthesis; d) naturally occurring *Aspidosperma* alkaloids; e) ring strain energy of cyclopentene, bicyclo[3.2.0]heptane, cycloheptane, cyclobutene, bicyclo[2.2.0]hexane and cyclohexane.

Cyclobutenone (**A**) is a versatile C4 synthon [11] – its [2 + 2] photocyclization yields **B**, featuring a strained bicyclo[2.2.0]hexane unit [12], which can fragment to form **C** (Figure 1a) [13–14]. However, competitive C1–C4 bond cleavage under irradiation or heating leads to ketene **D**, which can undergo cycloaddition with an alkene to yield **E**. This fragmentation pathway dominates under various conditions (e.g., transition-metal catalysis, nucleophilic addition) and is driven by ring-strain release [11].

PET, an alternative to direct excitation and EnT, enables the formation of unique radical intermediates [9–10]. We previously demonstrated the Ir-catalyzed [2 + 2] cyclization/*retro*-Mannich reaction of a tryptamine-substituted cyclopentenone **F**, which led to the formation of indoline **J** (Figure 1b) [15]. Unlike other reported methods [16–18], the PET reaction of **F** generates the cationic radical **G**, which initiates formation of **H**, which has a strained bicyclo [3.2.0]heptane core. Strain release of **H** triggers a downstream radical-driven *retro*-Mannich reaction, which ultimately results in the formation of **J** via reductive quenching of intermediate **I**.

As part of our current interest in the synthesis of complex natural products via photochemical reactions, we decided to achieve such an unusual bond cleavage (Figure 1a, path A) of cyclobutenone by generating a radical cation species via a PET reaction. The synthetic plan is shown in Figure 1c and includes a PET-initiated [2 + 2] cyclization of the tryptamine-substituted cyclobutenone **K** to form the radical cation **L**, which has a highly functionalized and rigid bicyclo[2.2.0]hexane core. Fragmentation of the C3–C19 bond would afford a redox-active intermediate which upon further reductive quenching would lead to the tetracyclic indoline **M**, which was expected to serve as a common intermediate for the total synthesis of *Aspidosperma* alkaloids. These alkaloids constitute a large family of structurally complex compounds, which incorporate a pentacyclic ABCDE skeleton (Figure 1d, **1**–**8**) [19–23]. However, the formation of intermediate **L** is challenging because its ring-strain energy (Figure 1e, 52.1 kcal/mol) is higher than that of its counterpart, i.e. the bicyclo[3.2.0]heptane motif (28.3 kcal/mol) in **H** [24].

Herein, we report our recent results on the development of a novel strategy for the stereoselective construction of the tetracyclic core of *Aspidosperma* alkaloids. Our method involves an Ir-catalyzed PET reaction of **K** for the stereoselective formation of the *cis*-configured BC bicyclic core with an all-carbon quaternary center [25–26]. Computational studies suggest that the observed tandem PET reaction of **K** proceeds via an unusual 1,3-C shift to afford **M**, i.e. through an interrupted [2 + 2] cyclization/*retro*-Mannich reaction.

## Results and Discussion

### Conditions optimization

Our study commenced with the evaluation of the proposed PET-based tandem [2 + 2] cyclization/*retro*-Mannich reaction. We selected compound **10a**, which has a spiro-pentacyclic core, as the target to be synthesized via the proposed PET reaction of substrate **9a** (Table 1). Substrate **9a** can be synthesized from tryptamine and a substituted cyclobutane-1,3-dione according to a published protocol [27].

**Table 1:** Conditions screening.^a^

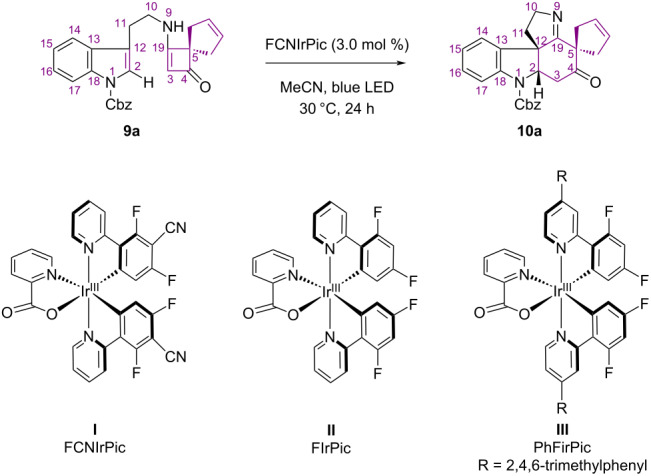				

Entry	Photocatalyst	Solvent	Temp.	Conv./yield (%)

1	FCNIrPic (**I**)	MeCN	30 °C	100/86^b^
2	FirPic (**II**)	MeCN	30 °C	83/26
3	PhFIrPic (**III**)	MeCN	30 °C	65/43
4	in dark	MeCN	30 °C	<5/n.d.
5	no catalyst	MeCN	30 °C	<5/n.d.
6	FCNIrPic (**I**)	MeOH	30 °C	84/23
7	FCNIrPic (**I**)	THF	30 °C	30/20
8	FCNIrPic (**I**)	DCM	30 °C	59/35
9	FCNIrPic (**I**)	MeCN	30 °C	86/42^c^
10	FCNIrPic (**I**)	MeCN/PhMe	30 °C	67/56^c,d^
11	FCNIrPic (**I**)	MeCN	10 °C	100/60
12	FCNIrPic (**I**)	MeCN	20 °C	100/66
13	FCNIrPic (**I**)	MeCN	40 °C	90/50

^a^Reaction conditions: A 15 mL glass vial was charged with **9a** (0.1 mmol) and a photocatalyst (3.0 mol %) in an appropriate solvent (5.0 mL), and irradiated by two blue LEDs (center wavelength 455 nm; light intensity, 0.21 W/cm^2^). The yield and conversion were determined by ^1^H NMR spectroscopy with 1,3,5-trimethoxybenzene as the internal standard. ^b^Isolated yield = 86%. ^c^Cbz is replaced by -Boc (**9f**). ^d^*V*_MeCN_/*V*_PhMe_ = 10:1.

Initially, we focused on identifying catalysts that could promote the proposed PET reaction of **9a** on the basis of our previous results [15]. The reaction was performed in MeCN in the presence of photocatalysts under blue light-emitting diode (LED) irradiation at 30 °C. The results are listed in Table 1. Catalysts **I** [28], **II** [29], and **III** [30] gave the desired product **10a**, with catalyst **I** giving the best result (Table 1, entries 1–3). The necessities of irradiation and the presence of a photocatalyst were also defined (Table 1, entries 4 and 5). However, use of the other tested catalysts did not give the desired product under the reaction conditions (see Supporting Information File 1 for details). Next, we evaluated the effects of different solvents on the production of **10a** in the presence of catalyst **I**. Changing the solvent to MeOH, tetrahydrofuran (THF), and dichloromethane (DCM) resulted in decreased yields and substrate conversions, and intense substrate decomposition (Table 1, entries 6–8). These results showed that MeCN is the best solvent for the reaction.

Finally, we investigated the effects of an alternative N-substituent in the indole moiety, and the reaction time and temperature on the photocyclization results. Boc-substituted substrate **9f** was subjected to the optimized conditions, which resulted in both a lower conversion and yield (Table 1, entry 9). In contrast, when the reaction of **9f** was performed in a mixed MeCN/toluene 10:1 solvent, the conversion of substrate **9f** to product **10f** decreased, but the yield increased to 56% (Table 1, entry 10) compared with that in entry 9 (42%). These results indicate that Cbz is a more effective protecting group than Boc under the profiled conditions. The effects of the reaction temperature on the outcome of the PET reaction of **9a** were investigated. Among the conditions screened (Table 1, entries 11–13), the reaction in MeCN at 30 °C for 24 h gave the best result, namely a quantitative conversion and 90% yield.

### Substrate scope

**Scheme 1:**
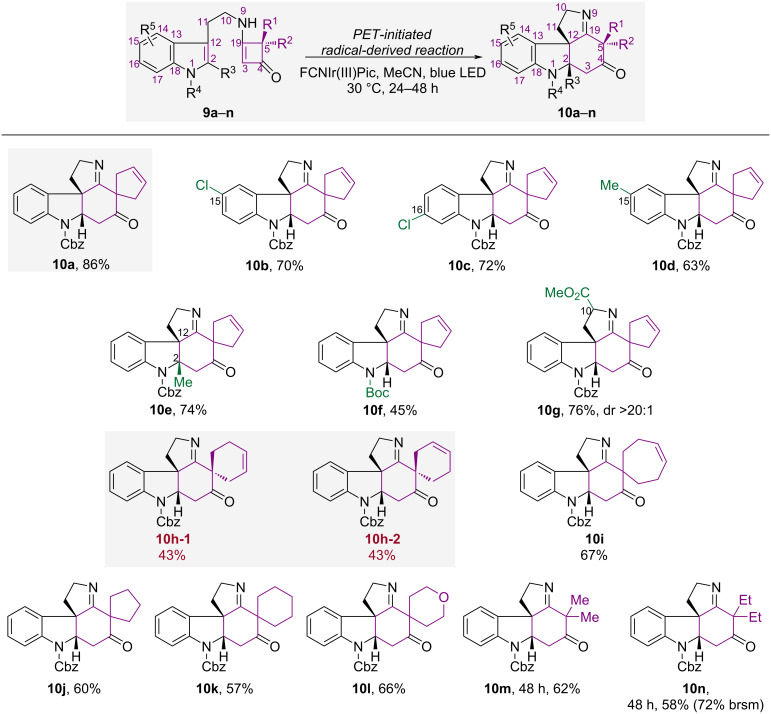
Substrate scope.

With the optimal conditions in hand, we then explored the substrate scope. Targeting on the total synthesis of *Aspidosperma* alkaloids, different tryptamine-substituted cyclobutenones **9a**–**n** were prepared and reacted under the optimal conditions. The results are shown in Scheme 1. Substrate **9a**, which contains a spiro-cyclopentene moiety, delivered the best result and gave **10a** in 86% isolated yield. When C15 or C16 was substituted with a chlorine atom, **10b** and **10c** were obtained in 70% and 72% yield, respectively. However, when C15 was substituted with a methyl group, the yield of **10d** decreased slightly to 63%. Product **10e**, which has two contiguous quaternary stereogenic centers at C2 and C12, was obtained in 74% yield without a significant change in the yield, which indicates that steric hindrance at C2 has a limited effect on the reactivity. However, when the steric hindrance of the protecting group was increased by replacing –Cbz with –Boc, the activity of the PET reaction decreased, which resulted in a lower yield of **10f** (45%). In the case of substrate **9g**, which has a stereogenic centre at C10, photocyclization afforded **10g** in moderate yield and with excellent diastereoselectivity (76% yield, dr >20:1). This indicates that the reaction is controlled by the substrate conformation.

To investigate the effects of the spirocyclic ring size on the photocyclization, substrates **9h** and **9i** were prepared and subjected to the optimal conditions. The PET reaction of **9h** with a spiro-cyclohexene unit gave a pair of separable isomers, **10h-1** and **10h-2**, in 86% yield and a 1:1 ratio. The reaction of **9i**, which bears a spiro-cycloheptene moiety, afforded the annulated product **10i** in 67% yield. This indicates that an increase in the spirocyclic ring size negatively affects the photocyclization outcome. We expanded the reaction scope by synthesizing substrates **9j**–**l**, which contain various types of saturated spirocycles. As anticipated, **10j**, **10k**, and **10l** were obtained in moderate yields (57–66%) under the standard reaction conditions. When substrates **9m** and **9n**, which have a *gem*-dimethyl and *gem*-diethyl group, respectively, were used, their PET reactions required a longer reaction time to achieve full conversion. The resulting products **10m** and **10n** were obtained in yields of 62% and 58%, respectively.

### Computational study

**Figure 2:**
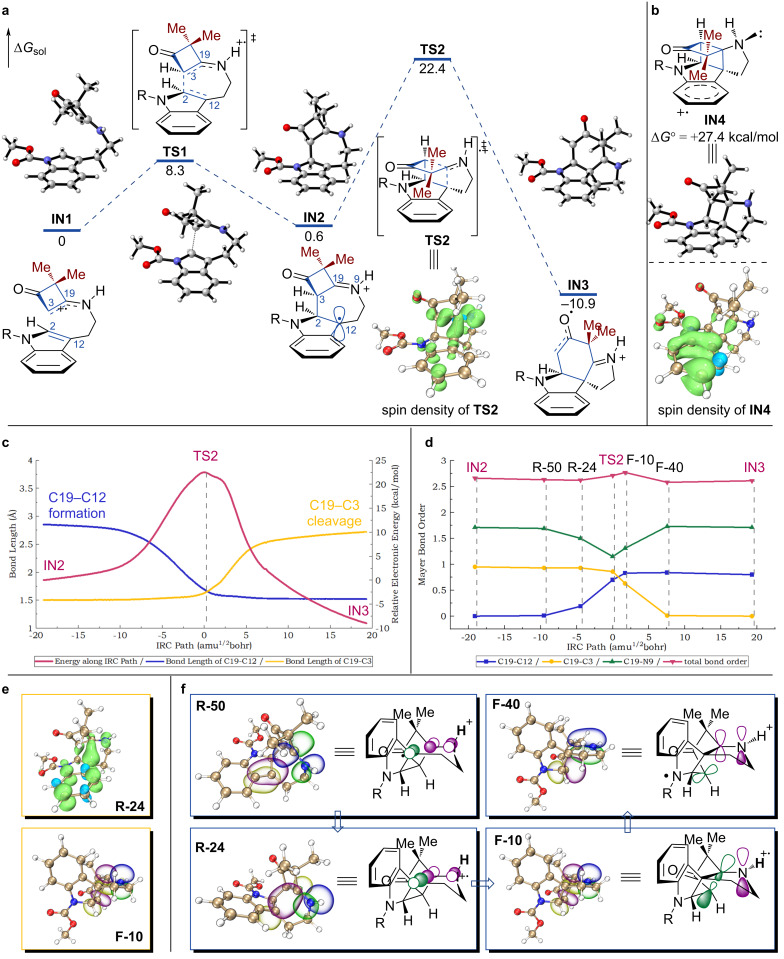
Computational study. a) Energy profiles from **IN1** to **IN3** and spin density of **TS2** (isovalue = 0.004), solvated (SMD) Gibbs free energies were calculated at the B3LYP-D3/def2-tzvp//B3LYP/6-31G(d) level in MeCN. b) Spin density of **IN4** (isovalue = 0.004). c) IRC analysis of **TS2** (red line) and bond-length changes of C19–C3 (yellow line) and C19–C12 (blue line) along IRC path. d) Mayer bond order changes of selected structures along IRC path. e) Spin-density analyses of **R-24** and **F-10** (isovalue = 0.004). f) NBO analyses of the selected transient structures (isovalue = 0.04).

The synthesis of (±)-aspidospermidine (**1**) and (±)-limaspermidine (**2**) showcased the effectiveness of our strategy for constructing complex monoterpene indole alkaloids [26]. In this work, we turned our attention to investigating the mechanistic intricacies of the key PET reaction for formation of the unique bicyclo[2.2.0]hexane unit present in the proposed intermediate **L** (Figure 1). In the presence of the excited photocatalyst [FCNIr(III)Pic]*, the substrate participates in an oxidative single-electron transfer (SET) process, which leads to the formation of **IN1**. The radical cation **IN1** served as the reference point for DFT investigations. As illustrated in Figure 2a, facilitated by a favorable radical cation–π interaction [31], **IN1** proceeds to the first radical addition transition state (**TS1**), with an energy barrier of 8.3 kcal/mol. This leads to the formation of benzyl radical **IN2**, which has a boat-like seven-membered ring. This structural feature may facilitate formation of the C19–C12 bond, even in the presence of critical steric hindrance between the C5 quaternary carbon and the indole moiety.

Further DFT investigation proved challenging because of the peculiar features that the potential energy surface (PES) exhibits in the rate-determining step, which involves both formation of the C19–C12 bond and cleavage of the C19–C3 bond. This process has an energy barrier of 21.8 kcal/mol via **TS2**, and results in direct formation of **IN3**, which can undergo reductive SET in the presence of [FCNIr(II)Pic]^−^. This leads to the regeneration of [FCNIr(III)Pic] to complete the catalytic cycle and formation of the final product via proton transfer. Intrinsic reaction coordinate (IRC) analysis showed the reaction coordinate connecting the transition state **TS2**, the reactant (**IN2**), and the C19–C3 bond cleavage product **IN3** wells (Figure 2c).

Possible intermediates with a bicyclo[2.2.0]hexane unit located on the PES minimum were investigated by performing two-dimensional scans of simplified structures to find a minimum. When the nonbonding sp^3^ orbital of the N atom is positioned antiperiplanar to the adjacent C19–C3 bond, an intermediate **IN4** with a bicyclo[2.2.0]hexane unit was successfully located. However, the Gibbs free energy of **IN4** (Δ*G*° = +27.4 kcal/mol) is significantly higher than the activation energy of **TS2** (Δ*G*^‡^ = 22.4 kcal/mol). This energy difference can be attributed to a subtle discrepancy between the spin localizations of **IN4** and **TS2**. As depicted in Figure 2b, the spin density of **IN4** is predominantly concentrated at the benzene ring, whereas in **TS2** it is primarily localized at C19, C12, C3, and N9 (Figure 2a) [32]. Therefore, the intermediacy of **IN4** was ruled out.

We assume that the overlap between the sp^2^-hybridized N spin center and σ* (C19–C3) in **TS2**, and the ring-strain release of the transient bicyclo[2.2.0]hexane unit, play essential roles that enable the reaction to occur. Thus, natural bond orbital (NBO) [33] and Mayer bond order [34–35] analyses were performed to determine the hyperconjugative interactions in the intermediates, transition states, and transient structures along the IRC path (Figure 2c and 2d). In the early stage of this process (Figure 2f, **IN2** → **R-50** → **R-24** → **TS2**), formation of the C19–C3 bond arises from the orbital overlap between the p orbital of the C19 atom (Figure 2e, **R-24**) and the π_(C3=N9)_* orbital. During this process, the bond order of C19–C12 increases, but no significant change is observed for C19–C3. This leads to the formation of a nonbonding p orbital at the N9 atom (Figure 2e, **F-10**). Further geometrical adjustment and conformational restriction of the transient structure enable the N9 nonbonding p orbital to align parallel to the σ_(C19–C3)_* orbital (Figure 2f, **TS2** → **F-10** → **F-40** → **IN3**), which reinforces the hyperconjugative interaction. Facilitated by the bond stretching and bond-angle bending of the transient structure with a pseudo bicyclo[2.2.0]hexane unit, the favorable hyperconjugative interaction ultimately leads to cleavage of the C19–C3 bond (**TS2** → **IN3**) and release of the ring strain.

DFT analysis hereby explains that the orbital symmetry involved in this process does not conform to a sigmatropic rearrangement reaction [36]. Inspection of the changes in the bond lengths (Figure 2c) and Mayer bond orders (Figure 2d) along the IRC path clearly show that C19–C12 bond formation and C19–C3 bond cleavage are asynchronous. On the basis of these premises, we assume that the process **IN2** → **IN3** is neither strictly concerted nor stepwise [37–38]. This can be attributed to the inherent ring-strain release in the transient structure located on the PES and the hyperconjugative interaction between the N9 nonbonding p orbital and σ_(C19–C3)_* during geometrical distortions. This ambiguous mechanistic feature suggests an unusual 1,3-C shift, and indicates that this reaction proceeds via a PET-initiated interrupted [2 + 2]/*retro*-Mannich process.

## Conclusion

In summary, a PET-initiated cationic radical-derived interrupted [2 + 2]/*retro*-Mannich reaction has been developed for constructing the ABCE tetracyclic cores of *Aspidosperma* alkaloids from tryptamine-substituted cyclobutenones. Importantly, this methodology has already been successfully applied in the total syntheses of (±)-aspidospermidine and (±)-limaspermidine using **10a** and **10h** as substrates, respectively [26]. The functionalized C5 atom in the formed ABCE tetracyclic core provides potential opportunities for accessing more complex indole alkaloids. The extensive DFT study indicated that the observed PET-initiated cationic radical-derived reaction proceeds via an unconventional formal 1,3-C shift, which is neither concerted nor stepwise. These findings shed light on the mechanistic innovation of a PET-initiated radical-derived reaction that was driven by the ring-strain release.

## Supporting Information

File 1 Experimental procedures, characterization data, NMR spectra, and computational study.

## Data Availability

All data that supports the findings of this study is available in the published article and/or the supporting information of this article.
