# Supplementary material for: Synthesis of the tetracyclic skeleton of Aspidosperma alkaloids via PET-initiated cationic radical-derived interrupted [2 + 2]/retro-Mannich reaction
Source: Beilstein J Org Chem. 2025 Nov 10;21:2470–8. doi: 10.3762/bjoc.21.189 (PMC12621632; doi:10.3762/bjoc.21.189)
Supplement: File 1 — Experimental procedures, characterization data, NMR spectra, and computational study. [file Beilstein_J_Org_Chem-21-2470-s001.pdf]

BEILSTEIN JOURNAL OF ORGANIC CHEMISTRY

Supporting Information

for

Synthesis of the tetracyclic skeleton of *Aspidosperma* alkaloids via PET-initiated cationic radical-derived interrupted [2 + 2]/*retro*-Mannich reaction

Ru-Dong Liu, Jian-Yu Long, Zhi-Lin Song, Zhen Yang and Zhong-Chao Zhang

Beilstein J. Org. Chem. **2025,** *21,* 2470–2478. doi:10.3762/bjoc.21.189

Experimental procedures, characterization data, NMR spectra, and computational study

License and Terms: This is a supporting information file under the terms of the Creative Commons Attribution License (https://creativecommons.org/licenses/by/4.0). Please note that the reuse, redistribution and reproduction in particular requires that the author(s) and source are credited and that individual graphics may be subject to special legal provisions.
The license is subject to the *Beilstein Journal of Organic Chemistry* terms and conditions: (https://www.beilstein-journals.org/bjoc/terms)

Table of contents

I: Experimental procedures and spectroscopic data S2

1. Synthesis of substrates S3
2. Visible-light-induced [2 + 2]-cycloaddition/*retro*-Mannich reactions S8

II: Computational details S16

Table S3-1. Calculated imaginary frequencies of all transition states species S16

Table S3-2. Calculated energy values (in Hartee) S16

Two-dimension scans of simplified structures S17

Cartesian coordinates S18

III: ^{1}H NMR and ^{13}C NMR spectra S24

IV. References S48

I: Experimental procedures and spectroscopic data

General procedure.

Unless otherwise mentioned, all reactions were carried out under an argon atmosphere with dry solvents. Reagents were purchased at the highest commercial quality and used without further purification, unless otherwise stated. Solvents purification was conducted according to purification of laboratory chemicals (Peerrin, D. D.; Armarego, W. L. and Perrins, D. R., Pergamon Press: Oxford, 1980). Concentration of solutions was accomplished using a Büchi rotary evaporator with a water aspirator. Yields refer to chromatographically and spectroscopically (^{1}H NMR) homogeneous materials. This was generally followed by removal of residual solvents on a vacuum line held at 0.1–1 torr.

Reactions were monitored by thin-layer chromatography (TLC) carried out on 0.25 mm Tsingdao silica gel plates (GF-254). Visualization on TLC was achieved by use of UV light at 254 nm. Staining was performed with an ethanolic solution of phosphomolybdic acid (PMA) and cerium sulfate, or by oxidative staining with an aqueous basic potassium permanganate ($KMnO_4$) solution and subsequent heating. Tsingdao silica gel (60, particle size 0.040–0.063 mm) was used for flash column chromatography.

NMR spectra were recorded on either a Bruker Advance 400 (^{1}H: 400 MHz, ^{13}C: 100 MHz) or Bruker Advance 500 (^{1}H: 500 MHz, ^{13}C: 126 MHz) and were calibrated using residual undeuterated solvent or TMS (tetramethylsilane) as an internal reference ($CDCl_3$: ^{1}H NMR = 7.26 ppm, ^{13}C NMR = 77.2 ppm; CD_3CN: ^{1}H NMR = 1.94 ppm, ^{3}C NMR = 118.7, 1.39 ppm; TMS: ^{1}H NMR = 0.00 ppm, ^{13}C NMR = 0.00 ppm.). The following abbreviations were used to explain the multiplicities: s = singlet, d = doublet, dd = doublet of doublets， t = triplet, q = quartet, m = multiplet. High resolution mass spectrometric (HRMS) data were recorded on a Bruker Apex IV RTMS instrument and a VG Auto Spec-3000 spectrometer, respectively.

1. Synthesis of substrates

General procedure A

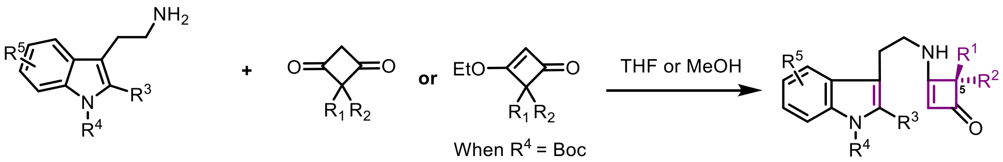

S1 S2 9a-m

Tryptamine **S1** and **S2** can be obtained by revised published methods [1,2]. **S1** (1.5 equiv) and **S2** (1.0 equiv) were dissolved in THF or MeOH at room temperature, and then the reaction mixture was stirred at 35 °C or 60 °C until compound **S2** was consumed. The solvent was removed under vacuum, and the residue was purified by flash chromatography on silica gel (petroleum ether/ethyl acetate =2:1—1:2) to give products **9a–m**.

General procedure B

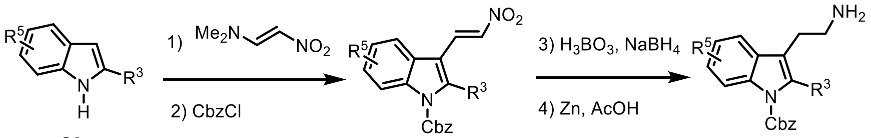

S3 S4 S1

To a solution of **S3** (1.0 equiv) and 1-dimethylamino-2-nitroethylene (0.8 equiv) in dry dichloromethane was added TFA (8.0 equiv) slowly at 0 °C, and then the reaction mixture was stirred at room temperature until 1-dimethylamino-2-nitroethylene was consumed. The reaction was quenched by slow addition of saturated solution of Na_2CO_3 and the resultant mixture was extracted with ethyl acetate. The combined organic extracts were washed with brine, and dried over Na_2SO_4. The solvent was removed under vacuum, and the residue was dissolved in dry dichloromethane at room temperature. To this solution were added consecutively NaOH (3.0 equiv), $(n\text{-Bu})_4NHSO_4$ (0.1 equiv), and benzyl chloroformate (3.0 equiv) subsequently at room temperature. The reaction mixture was stirred at room temperature for 3–8 h. The reaction was quenched by slow addition of saturated solution of NH_4Cl and the resultant mixture was extracted with dichloromethane. The combined organic extracts were washed with brine, and the solvent was removed under vacuum to yield crude product **S4**.

To the solution of **S4** (1.0 equiv) and H_3BO_3 (6.0 equiv) in THF/isopropanol 1:2 was added $NaBH_4$ (6.0 equiv) in portions at room temperature, and then the reaction mixture was stirred at room temperature for 2–4 h. The reaction was quenched by addition of 1 M HCl and saturated solution of NH_4Cl slowly, and the resultant mixture was extracted with ethyl acetate. The combined organic extracts were washed with brine, and the solvent was removed under vacuum to yield a crude product which was dissolved in acetic acid. Zn powder (3.0 equiv) was slowly added at room temperature, and then the reaction mixture was stirred at room temperature overnight. The reaction was quenched by slow addition of a saturated solution of K_2CO_3 and the resultant mixture was extracted with ethyl acetate. The combined organic extracts were washed with brine, and dried over with Na_2SO_4. The solvent was removed under vacuum, and the residue was purified by flash chromatography on silica gel (dichloromethane/methanol 20:1 to 10:1) to give tryptamine **S1**.

Characterization of substrates

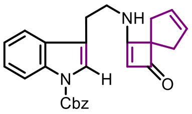

9a

Compound **9a** was synthesized following general procedure A, brown foam, yield: 75%; characterization data was identical with previously reported data [3].

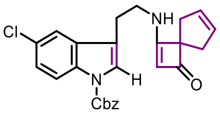

9b

Compound **9b** was synthesized following general procedure A, brown foam, yield: 65%;

R*f* = 0.20 (petroleum ether/ethyl acetate = 1:1);

^{1}H NMR (400 MHz, Chloroform-*d*) δ 8.18 – 7.91 (m, 1H), 7.50 – 7.36 (m, 7H), 7.33 – 7.25 (m, 1H), 5.78 (t, *J* = 6.1 Hz, 1H), 5.56 (s, 2H), 5.43 (s, 2H), 4.57 (s, 1H), 3.50 (q, *J* = 6.6 Hz, 2H), 2.95 (t, *J* = 6.7 Hz, 2H), 2.67 (d, *J* = 15.7 Hz, 2H), 2.35 (d, *J* = 15.9 Hz, 2H) ppm;

^{13}C NMR (101 MHz, Chloroform-*d*) δ 189.1, 175.3, 150.4, 134.8, 134.1, 131.4, 130.0, 129.5, 129.1, 129.0, 129.0, 128.7, 125.3, 118.5, 117.2, 116.7, 98.0, 69.2, 66.5, 44.9, 36.6, 24.7 ppm;

HRMS (ESI): *m*/*z* calcd. for $C_{26}H_{24}N_2O_3Cl$ $[M+H]^+$: 447.1470, found: 447.1462.

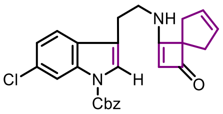

9c

Compound **9c** was synthesized following general procedure A, brown foam, yield: 79%;

R*f* = 0.20 (petroleum ether/ethyl acetate = 1:1);

^{1}H NMR (400 MHz, Chloroform-*d*) δ 8.20 (s, 1H), 7.42 (m, 7H), 7.23 (t, *J* = 11.1 Hz, 1H), 6.07 (t, *J* = 43.5 Hz, 1H), 5.54 (s, 2H), 5.42 (s, 2H), 4.53 (d, *J* = 6.6 Hz, 1H), 3.50 (q, *J* = 6.8 Hz, 2H), 2.95 (d, *J* = 7.1 Hz, 2H), 2.63 (d, *J* = 16.9 Hz, 2H), 2.35 (d, *J* = 16.7 Hz, 2H) ppm;

^{13}C NMR (101 MHz, Chloroform-*d*) δ 189.3, 175.4, 150.4, 136.1, 134.9, 131.2, 130.0, 129.5, 129.1, 129.0, 128.7, 123.7, 123.6, 119.6, 117.6, 115.9, 97.8, 69.2, 66.3, 44.9, 36.6, 24.7 ppm;

HRMS (ESI): *m*/*z* calcd. for $C_{26}H_{22}N_2O_3Cl$ $[M-H]^-$: 445.1324, found: 445.1325.

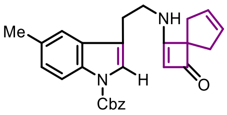

9d

Compound **9d** was synthesized following general procedure A, brown foam, yield: 42%;

R*f* = 0.20 (petroleum ether/ethyl acetate = 1:1);

^{1}H NMR (400 MHz, Chloroform-*d*) δ 8.04 (s, 1H), 7.49 – 7.33 (m, 6H), 7.29 – 7.22 (m, 1H), 7.16 (dd, *J* = 8.4, 1.7 Hz, 1H), 5.72 (t, *J* = 6.1 Hz, 1H), 5.55 (s, 2H), 5.41 (s, 2H), 4.60 (s, 1H), 3.51 (q, *J* = 6.6 Hz, 2H), 2.96 (t, *J* = 6.7 Hz, 2H), 2.68 (d, *J* = 15.4 Hz, 2H), 2.44 (s, 3H), 2.35 (d, *J* = 15.3 Hz, 2H) ppm;

^{13}C NMR (101 MHz, Chloroform-*d*) δ 189.2, 175.3, 150.7, 135.1, 133.9, 132.8, 130.3, 129.9, 129.5, 128.9, 128.6, 126.5, 123.2, 118.7, 117.4, 115.3, 98.0, 68.8, 66.4, 44.9, 36.6, 24.8, 21.5 ppm;

HRMS (ESI): *m*/*z* calcd. for $C_{27}H_{27}N_2O_3$ $[M+H]^+$: 427.2016, found: 427.2011.

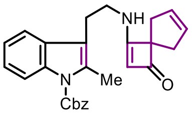

9e

Compound **9e** was synthesized following general procedure A, brown foam, yield: 77%;

R*f* = 0.20 (petroleum ether/ethyl acetate = 1:1);

^{1}H NMR (400 MHz, Chloroform-*d*) δ 8.13 – 8.07 (m, 1H), 7.52 – 7.45 (m, 2H), 7.45 – 7.28 (m, 4H), 7.28 – 7.20 (m, 2H), 5.56 (s, 2H), 5.50 (d, *J* = 6.1 Hz, 1H), 5.46 (s, 2H), 4.61 (s, 1H), 3.46 (q, *J* = 6.6 Hz, 2H), 2.97 (t, *J* = 6.7 Hz, 2H), 2.68 (d, *J* = 15.9 Hz, 2H), 2.54 (s, 3H), 2.31 (d, *J* = 15.3 Hz, 2H) ppm;

^{13}C NMR (101 MHz, Chloroform-*d*) δ 189.3, 175.4, 152.1, 135.9, 135.1, 134.5, 129.8, 129.6, 129.0, 129.0, 128.8, 124.3, 123.3, 117.6, 116.0, 115.0, 97.9, 69.0, 66.5, 45.3, 36.6, 24.0, 14.2 ppm;

HRMS (ESI): *m*/*z* calcd. for $C_{27}H_{27}N_2O_3$ $[M+H]^+$: 427.2016, found: 427.2011.

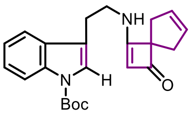

9f

Compound **9f** was synthesized following revised reported procedure [4], brown foam, yield: 53%;

R*f* = 0.20 (petroleum ether/ethyl acetate = 1:1);

^{1}H NMR (400 MHz, Chloroform-*d*) δ 8.14 (d, *J* = 8.3 Hz, 1H), 7.49 (d, *J* = 7.8 Hz, 1H), 7.41 (s, 1H), 7.33 (t, *J* = 7.7 Hz, 1H), 7.26 (d, *J* = 7.7 Hz, 1H), 6.00 (t, *J* = 6.1 Hz, 1H), 5.59 (s, 2H), 4.58 (s, 1H), 3.53 (q, *J* = 6.7 Hz, 2H), 3.01 (t, *J* = 6.8 Hz, 2H), 2.69 (d, *J* = 16.8 Hz, 2H), 2.41 (d, *J* = 16.7 Hz, 2H), 1.66 (s, 9H).

^{13}C NMR (101 MHz, Chloroform-*d*) δ 189.3, 175.4, 149.6, 135.6, 130.1, 129.5, 124.8, 123.5, 122.7, 118.6, 116.7, 115.6, 97.8, 83.9, 66.3, 45.0, 36.6, 28.3, 24.8 ppm.

HRMS (ESI): *m*/*z* calcd. for $C_{23}H_{27}N_2O_3$ $[M+H]^+$: 379.2016, found: 379.2017.

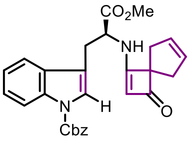

9g

Compound **9g** was synthesized following general procedure A, white foam, yield: 50%;

R*f* = 0.20 (petroleum ether/ethyl acetate = 1:1);

^{1}H NMR (500 MHz, Chloroform-*d*) δ 8.17 (s, 1H), 7.54 – 7.30 (m, 8H), 7.26 (t, *J* = 7.5 Hz, 1H), 5.96 (d, *J* = 7.9 Hz, 1H), 5.63 – 5.45 (m, 2H), 5.43 (d, *J* = 2.9 Hz, 2H), 4.55 (s, 1H), 4.34 (q, *J* = 5.9 Hz, 1H), 3.74 (s, 3H), 3.34 (dd, *J* = 14.8, 5.1 Hz, 1H), 3.24 (dd, *J* = 14.8, 6.0 Hz, 1H), 2.71 – 2.58 (m, 2H), 2.41 (d, *J* = 17.2 Hz, 1H), 2.28 (d, *J* = 17.4 Hz, 1H) ppm;

^{13}C NMR (126 MHz, Chloroform-*d*) δ 189.7, 174.2, 170.9, 150.7, 135.6, 135.0, 130.2, 129.5, 129.5, 129.1, 129.0, 128.8, 125.4, 124.2, 123.3, 118.6, 115.7, 114.8, 99.2, 69.2, 66.9, 57.6, 53.2, 42.7, 36.6, 27.3 ppm;

HRMS (ESI): *m*/*z* calcd for $C_{28}H_{27}N_2O_5$ $[M+H]^+$: 471.1915, found: 471.1923.

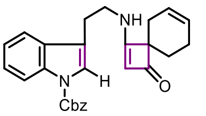

9h

Compound **9h** was synthesized following general procedure A, white foam, yield: 66%;
Characterization data was identical with previously reported data [3].

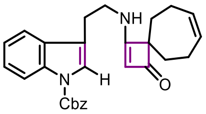

9i

Compound **9i** was synthesized following general procedure A, brown foam, yield: 22%;

R*f* = 0.40 (petroleum ether/ethyl acetate = 1:1);

^{1}H NMR (400 MHz, Chloroform-*d*) δ 8.22 – 7.77 (m, 1H), 7.46 – 7.27 (m, 8H), 7.23 – 7.17 (m, 1H), 5.85 (s, 1H), 5.53 (t, *J* = 3.2 Hz, 2H), 5.36 (s, 2H), 4.48 (s, 1H), 3.44 (q, *J* = 6.5 Hz, 2H), 2.92 (t, *J* = 6.7 Hz, 2H), 2.40 – 2.30 (m, 2H), 2.00 – 1.85 (m, 4H), 1.72 – 1.60 (m, 2H) ppm;

^{13}C NMR (101 MHz, Chloroform-*d*) δ 192.2, 177.7, 150.7, 135.8, 135.1, 131.2, 130.1, 129.0, 128.9, 128.6, 125.2, 123.2, 123.1, 118.8, 117.7, 115.7, 96.1, 68.9, 65.3, 44.9, 32.0, 25.8, 24.7 ppm;

HRMS (ESI): *m/z* calcd. for $C_{28}H_{29}N_2O_3$ $[M+H]^+$: 441.2178, found: 441.2178.

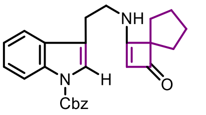

9j

Compound **9j** was synthesized following general procedure A, brown oil, yield: 21%;

R*f* = 0.20 (petroleum ether/ethyl acetate = 1:1);

^{1}H NMR (400 MHz, Chloroform-*d*) δ 8.27 – 8.04 (m, 1H), 7.54 – 7.30 (m, 8H), 7.26 (t, *J* = 7.4 Hz, 1H), 5.87 (d, *J* = 6.2 Hz, 1H), 5.42 (s, 2H), 4.55 (s, 1H), 3.52 (q, *J* = 6.6 Hz, 2H), 3.00 (t, *J* = 6.8 Hz, 2H), 1.85 (dd, *J* = 12.1, 6.2 Hz, 2H), 1.65 (m, 4H), 1.55 – 1.43 (m, 2H) ppm;

^{13}C NMR (101 MHz, Chloroform-*d*) δ 191.3, 176.2, 150.8, 135.8, 135.1, 130.2, 129.0, 129.0, 128.7, 125.3, 123.3, 123.2, 118.9, 117.9, 115.7, 97.5, 69.0, 67.9, 45.0, 30.7, 27.0, 24.8 ppm;

HRMS (ESI): *m/z* calcd. for $C_{26}H_{27}N_2O_3$ $[M+H]^+$: 415.2016, found: 415.2014.

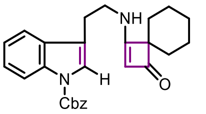

9k

Compound **9k** was synthesized following general procedure A, brown foam, yield: 63%;

R*f* = 0.20 (petroleum ether/ethyl acetate = 1:1);

^{1}H NMR (400 MHz, Chloroform-*d*) δ 8.17 (s, 1H), 7.57 – 7.29 (m, 8H), 7.25 (t, *J* = 7.4 Hz, 1H), 6.43 (s, 1H), 5.41 (s, 2H), 4.50 (s, 1H), 3.50 (q, *J* = 6.6 Hz, 2H), 2.99 (t, *J* = 6.8 Hz, 2H), 1.72 (q, *J* = 11.3, 8.4 Hz, 4H), 1.58 (dd, *J* = 13.6, 9.5 Hz, 2H), 1.50 – 1.39 (m, 3H), 1.30 – 1.20 (m, 1H) ppm;

^{13}C NMR (101 MHz, Chloroform-*d*) δ 193.0, 178.4, 150.7, 135.7, 135.1, 130.2, 128.9, 128.6, 125.1, 123.1, 123.1, 118.8, 117.9, 115.6, 95.5, 77.5, 76.9, 68.8, 62.4, 45.1, 31.5, 25.5, 24.7, 24.5 ppm;

HRMS (ESI): *m/z* calcd. for $C_{27}H_{29}N_2O_3$ $[M+H]^+$: 429.2173, found: 429.2176.

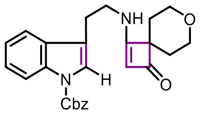

9l

Compound **9l** was synthesized following general procedure A, brown foam, yield: 42%;

R*f* = 0.20 (petroleum ether/ethyl acetate = 1:1);

^{1}H NMR (400 MHz, Chloroform-*d*) δ 8.23 – 7.94 (m, 1H), 7.46 – 7.14 (m, 9H), 6.23 (t, *J* = 5.9 Hz, 1H), 5.34 (s, 2H), 4.48 (s, 1H), 3.72 (m, 2H), 3.63 (m, 2H), 3.44 (q, *J* = 6.5 Hz, 2H), 2.92 (t, *J* = 6.6 Hz, 2H), 1.71 (m, 2H), 1.61 (m, 2H) ppm;

^{13}C NMR (101 MHz, Chloroform-*d*) δ 191.1, 176.4, 150.8, 135.8, 135.1, 130.2, 129.0, 129.0, 128.7, 125.3, 123.3, 123.2, 118.8, 117.7, 115.7, 96.6, 69.0, 66.0, 59.5, 45.1, 31.3, 24.7 ppm;

HRMS (ESI): *m/z* calcd. for $C_{26}H_{27}N_2O_4$ $[M+H]^+$: 431.1965, found: 431.1962.

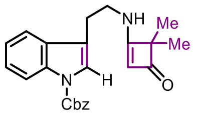

9m

Compound **9m** was synthesized following general procedure A, brown foam, yield: 36%;

R*f* = 0.20 (petroleum ether/ethyl acetate = 1:1);

^{1}H NMR (400 MHz, Chloroform-*d*) δ 8.18 (s, 1H), 7.41 (m, 8H), 7.28 (s, 1H), 6.09 (d, *J* = 6.3 Hz, 1H), 5.42 (s, 2H), 4.51 (s, 1H), 3.52 (q, *J* = 6.6 Hz, 2H), 3.00 (t, *J* = 6.8 Hz, 2H), 1.16 (s, 6H) ppm;

^{13}C NMR (101 MHz, Chloroform-*d*) δ 192.3, 177.9, 150.8, 135.8, 135.1, 130.2, 129.0, 129.0, 128.7, 125.3, 123.2, 118.9, 117.8, 115.7, 95.6, 69.0, 58.7, 45.0, 24.8, 20.2 ppm;

HRMS (ESI): *m/z* calcd for $C_{24}H_{24}N_2O_3Na$ $[M+Na]^+$: 411.1679, found: 411.1677.

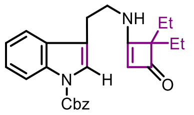

9n

Compound **9n** was synthesized following general procedure A, brown solid, yield: 46%;

R*f* = 0.20 (petroleum ether/ethyl acetate = 1:1);

^{1}H NMR (400 MHz, Chloroform-*d*) δ 8.18 (s, 1H), 7.53 – 7.30 (m, 8H), 7.29 – 7.26 (m, 1H), 6.03 (t, *J* = 6.0 Hz, 1H), 5.42 (s, 2H), 4.70 (s, 1H), 3.55 (q, *J* = 6.6 Hz, 2H), 3.04 – 2.94 (t, *J* = 6.9 Hz 2H), 1.59 (m, 2H), 1.44 (m, 2H), 0.82 (t, *J* = 7.5 Hz, 6H) ppm;

^{13}C NMR (101 MHz, Chloroform-*d*) δ 190.5, 174.4, 150.8, 135.8, 135.1, 130.2, 129.0, 129.0, 128.7, 125.3, 123.2, 123.1, 118.8, 117.8, 115.7, 99.1, 69.0, 67.7, 44.8, 25.1, 24.8, 9.5 ppm;

HRMS (ESI): *m/z* calcd. for $C_{26}H_{28}N_2O_3Na$ $[M+Na]^+$: 439.1992, found: 439.1991.

2. Visible-light-induced [2 + 2]-cycloaddition/*retro*-Mannich reactions

General procedure C

9 (0.10 mmol, 1.0 equiv) and FCNIrPic (0.003 mmol, 2.0 mg, 0.03 equiv) were dissolved in MeCN (5 mL), and the mixture was bubbled under ultrasound at room temperature for 5 min, which was then stirred under irradiation of blue LED (455 nm, 10 W) at 30 °C for 24 h or 48 h (**10m** and **10n**). The solvent was removed under vacuum, and the residue was purified by flash chromatography on silica gel to give unstable products **10**.

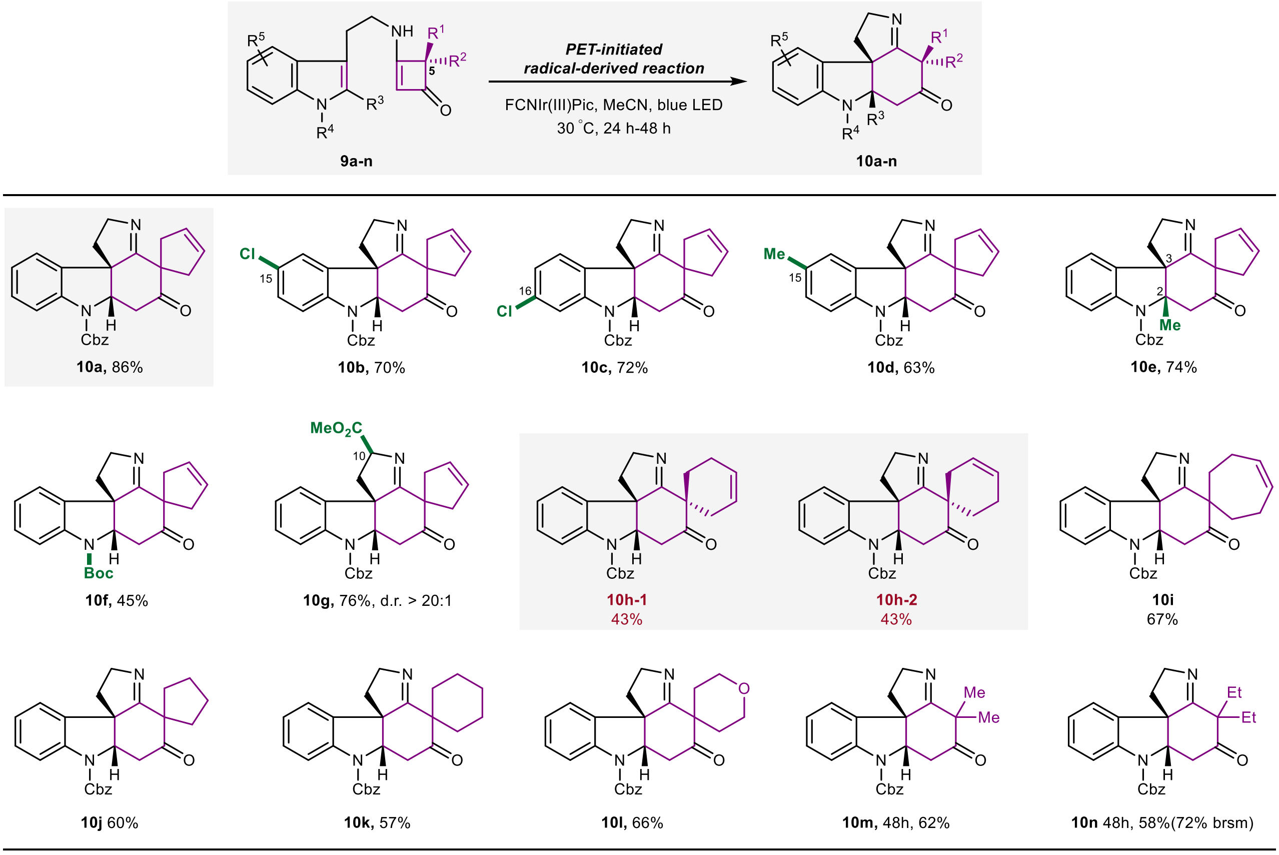

Figure S1-1. **Substrate scope**

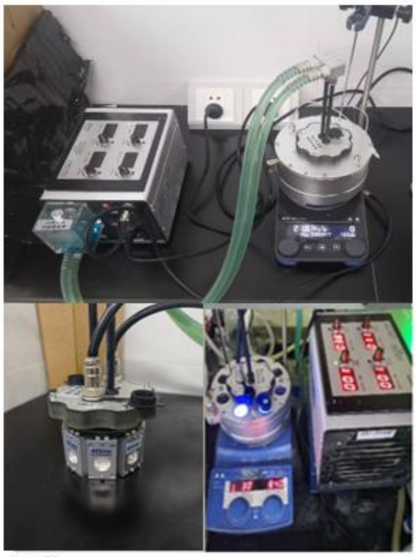

Parameter	Value	Parameter	Value	Parameter	Value
UV hazard irradiation (mW/cm²)	0.0000	Color Tolerance Adjustment	100.00	Coordinate (x, y)	0.1491,0.0282
UVC Irradiance (mW/cm²)	0.0000	color render index Ra	-70.5	Coordinate (u, v)	0.1962,0.0556
UVB Irradiance (mW/cm²)	0.0000	Irradiance Ee(mW/cm²)	205.57959	Coordinate (u', v')	0.1962,0.0834
UVA Irradiance (mW/cm²)	0.0000	S/P ratio	23.008	CIE1931 X	600616.125
Euv Irradiance (mW/cm²)	0.0000	Dominant wavelength (nm)	457.40	CIE1931 Y	113456.516
Eb Irradiance (mW/cm²)	205.32	Color purity (%)	99.2	CIE1931 Z	3313825.500
Eg Irradiance (mW/cm²)	0.54	Half width (nm)	19.6	TLCI-2012	0
Er Irradiance (mW/cm²)	0.00	Peak wavelength(nm)	453.5	Integral time (ms)	0.1
Eir Irradiance (mW/cm²)	0.00	Center wavelength (nm)	453.7	Peak signal	61384
Illuminance E(lx)	77490.80	Centroid wavelength (nm)	455.2	Dark signal	2287
Candlepower E(Fc)	7199.07	Luminous flux color ratio (RGB)	0.0,5.0,95.0	Compensation level	2880
Correlated color temperature (K)	100000	Bold body deviation (Duv)	-0.06722		

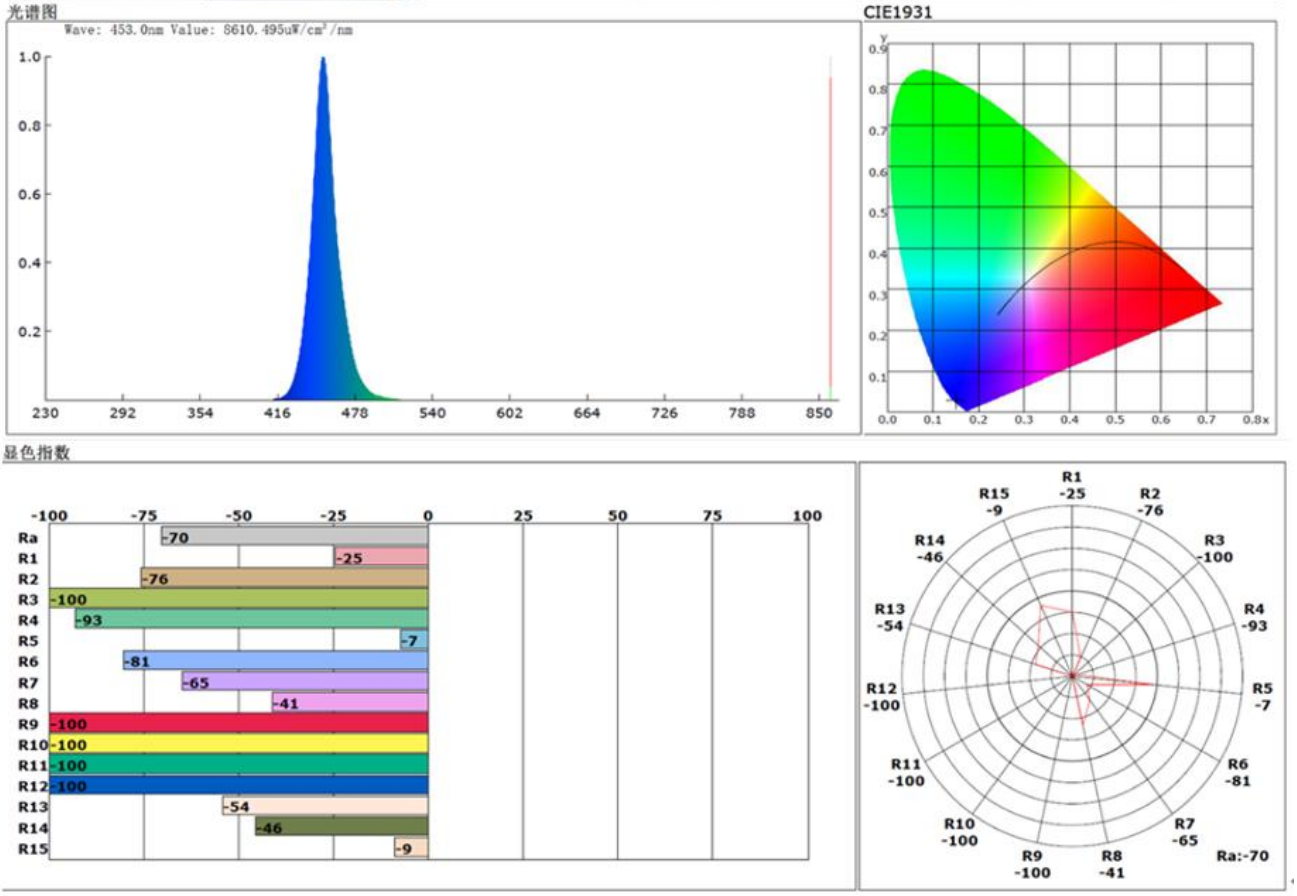

Figure S1-2. **The Photoreaction Apparatus**

RLH-18 8-position photo reaction system was used, manufactured by Beijing Rogertech Co. Ltd. based in Beijing PRC. This photoreactor was equipped with 8 LEDs (455 nm, 10 W), which could be selected and replaced in each position. The blue LEDs' energy peak wavelength was 453.0 nm; peak width at half-height was 19.6 nm; Irradiance@10 W is 206 mW/cm^2. The irradiation vessel was a borosilicate glass test tube. LED irradiated through a high-reflection channel to the test tube, whose path length was 2 cm. No filter was between the LED and the test tube.

Optimization of reaction conditions

Table S1-1. **Photocatalysts and solvents screening**

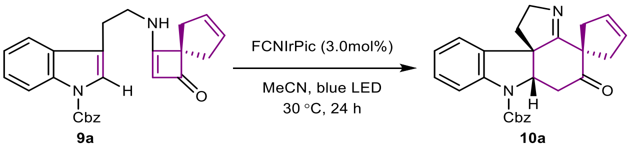

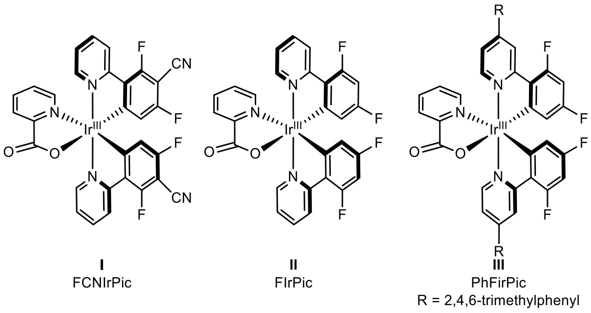

Entry	Difference from the Standard Condition	Conv./Yield (%)
	Photocatalyst	
**1**	none	100/90 (86 [a])
**2**	FIrPic (**II**)	83/26
**3**	PhFIrPic (**III**)	65/43
**4**	$Ir[CH_3ppy]_2(dtbbpy)PF_6$	< 5/N. D
**5**	$Ir[dF(CF_3)ppy]_2(dtbbpy)$ PF_6	< 5/N. D
**6**	$Ir[dF(CH_3)ppy]_2(dtbbpy)$ PF_6	< 5/N. D
**7**	$Ir[dF(CF_3)ppy]_2(bpy)PF_6$	< 5/N. D
**8**	$Ir[p\text{-}F(CH_3)ppy]_2(dtbbpy)PF_6$	< 5/N. D
**9**	$[Ru(bpz)_3][PF_6]_2$	< 5/N. D
**10**	2CzPN	< 5/N. D
**11**	4CzIPN	< 5/N. D
**12**	CF_3FIrPic I	< 5/N. D
**13**	In dark	< 5/N. D.
**14**	No catalyst	< 5/N. D.
	Solvent	
**15**	sol. = MeOH	84/23
**16**	sol. = THF	30/20
**17**	sol. = DCM	59/35
	N-substituent	
**18**	-Cbz is replaced by -Boc (**9f**)	86/42
**19**	-Cbz is replaced by -Boc (**9f**) [b]	67/56
	Ambient temperature	
**20**	Temp. = 10 °C	100/60
**21**	Temp. = 20 °C	100/66
**22**	Temp. = 40 °C	90/50

Reaction conditions: A 15 mL glass vial was charged with **9a** (0.1 mmol) and a photoredox catalyst (3.0 mol %) in an appropriate solvent (5.0 mL), and irradiated by two blue LEDs (center wavelength, 455 nm; light intensity, 0.21 W/cm^2). The yield and conversion were determined by ^{1}H NMR spectroscopy with 1,3,5-trimethoxybenzene as the internal standard. [a] Isolated yield. [b] Solvent V_{MeCN}/V_{PhMe} = 10:1

When the reaction scale was 1.00 mmol (the concentration was raised to 0.2 M), even the reaction was conducted for 168 h at 30 °C, the conversion and yield were both moderately declined, probably due to the comparably lowing of the light efficiency limited by the apparatus. We envisioned that it might be improved via flow chemistry.

Characterization of products

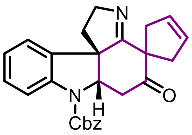

10a, 86%

Compound **10a** was synthesized following general procedure C, pale yellow foam, yield: 86%; Characterization data was identical with previously reported data [3].

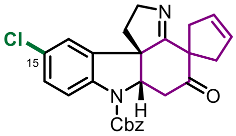

10b, 70%

Compound **10b** was synthesized following general procedure C, yellow foam, yield: 70%;

R*f* = 0.40 (petroleum ether/ethyl acetate = 1:1);

^{1}H NMR (400 MHz, Acetonitrile-*d*$_3$) δ 7.84 – 7.49 (m, 1H), 7.48 – 7.32 (m, 5H), 7.25 – 7.16 (m, 1H), 7.02 (d, *J* = 2.2 Hz, 1H), 5.46 (m, 2H), 5.27 (q, *J* = 12.4 Hz, 2H), 4.76 (dd, *J* = 5.8, 2.6 Hz, 1H), 4.18 – 3.98 (m, 2H), 3.34 (m, 1H), 2.92 (dd, *J* = 13.7, 5.8 Hz, 1H), 2.85 – 2.74 (m, 1H), 2.52 (m, 1H), 2.43 – 2.32 (m, 2H), 2.32 – 2.25 (m, 1H), 2.06 – 1.97 (m, 1H) ppm;

^{13}C NMR (101 MHz, Acetonitrile-*d*$_3$) δ 210.1, 180.0, 153.6, 141.6, 137.6, 136.7, 130.4, 130.0, 129.7, 129.6, 129.3, 129.2, 128.4, 125.5, 117.6, 68.9, 67.1, 62.7, 60.8, 60.0, 45.1, 44.7, 43.4, 43.0 ppm;

HRMS (ESI): *m*/*z* calcd. for $C_{26}H_{24}N_2O_3Cl$ [M+H]$^{+}$: 447.1470, found: 447.1468.

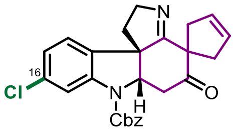

10c, 72%

Compound **10c** was synthesized following general procedure C, yellow foam, yield: 72%;

R*f* = 0.40 (petroleum ether/ethyl acetate = 1:1);

^{1}H NMR (400 MHz, Acetonitrile-*d*$_3$) δ 7.95 – 7.53 (m, 1H), 7.51 – 7.31 (m, 5H), 7.03 – 6.94 (m, 2H), 5.57 – 5.39 (m, 2H), 5.30 (q, *J* = 12.1 Hz, 2H), 4.78 (dd, *J* = 5.8, 2.6 Hz, 1H), 4.13 (m, 1H), 4.02 (m, 1H), 3.33 (m, 1H), 2.94 (dd, *J* = 13.7, 5.8 Hz, 1H), 2.80 (dd, *J* = 13.9, 2.6 Hz, 1H), 2.53 (m, 1H), 2.40 – 2.34 (m, 2H), 2.34 – 2.27 (m, 1H), 2.05 – 1.98 (m, 1H) ppm;

^{13}C NMR (101 MHz, Acetonitrile-*d*$_3$) δ 210.2, 180.4, 153.6, 144.0, 137.5, 135.5, 133.6, 130.0, 129.7, 129.6, 129.2, 128.4, 126.8, 124.7, 116.3, 69.0, 67.3, 62.4, 60.8, 60.0, 45.1, 44.8, 43.3, 42.9 ppm;

HRMS (ESI): *m*/*z* calcd. for $C_{26}H_{22}N_2O_3Cl$ [M-H]$^{-}$: 445.1324, found: 445.1325.

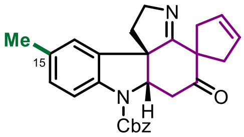

10d, 63%

Compound **10d** was synthesized following general procedure C, pale brown foam, yield: 63%;

R*f* = 0.50 (petroleum ether/ethyl acetate = 1:1);

^{1}H NMR (500 MHz, Acetonitrile-*d*$_3$) δ 7.58 (d, *J* = 72.0 Hz, 1H), 7.49 – 7.30 (m, 5H), 7.03 (q, *J* = 8.5, 6.1 Hz, 1H), 6.83 (d, *J* = 1.7 Hz, 1H), 5.46 (m, 2H), 5.33 – 5.19 (m, 2H), 4.72 (dd, *J* = 5.8, 2.7 Hz, 1H), 4.06 (m, 2H), 3.33 (m, 1H),

2.90 (dd, J = 13.6, 5.8 Hz, 1H), 2.81 (s, 1H), 2.51 (m, 1H), 2.42 – 2.30 (m, 2H), 2.27 (q, J = 2.5 Hz, 1H), 2.02 (d, J = 16.8 Hz, 1H) ppm;

^{13}C NMR (126 MHz, Acetonitrile-d_3) δ 210.4, 181.0, 153.6, 140.4, 137.9, 134.8, 134.7, 131.0, 130.0, 129.6, 129.5, 129.3, 128.4, 125.9, 116.1, 68.6, 66.9, 62.9, 60.8, 59.9, 45.1, 44.7, 43.5, 43.0, 21.3 ppm;

HRMS (ESI): m/z calcd. for $C_{27}H_{27}N_2O_3$ $[M+H]^+$: 427.2016, found: 427.2011.

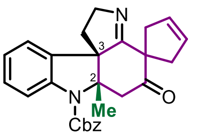

10e, 74%

Compound **10e** was synthesized following general procedure C, yellow oil, yield: 74%;

R$_f$ = 0.40 (petroleum ether/ethyl acetate = 1:1);

^{1}H NMR (500 MHz, Acetonitrile-d_3) δ 7.73 (s, 1H), 7.47 (d, J = 7.5 Hz, 2H), 7.39 (m, 2H), 7.34 (dd, J = 8.5, 6.2 Hz, 1H), 7.19 (dd, J = 13.4, 7.5 Hz, 2H), 7.00 – 6.94 (m, 1H), 5.52 (m, 1H), 5.28 (m, 2H), 5.21 (m, 1H), 4.16 – 3.96 (m, 2H), 3.46 – 3.30 (m, 2H), 2.64 – 2.50 (m, 2H), 2.44 (m, 1H), 2.33 – 2.25 (m, 1H), 2.23 – 2.14 (m, 1H), 1.53 (m, 1H), 1.47 (d, J = 2.3 Hz, 3H) ppm;

^{13}C NMR (126 MHz, Acetonitrile-d_3) δ 212.0, 180.0, 153.4, 141.6, 137.2, 135.0, 130.0, 129.6, 129.2, 129.0, 127.6, 124.9, 124.4, 116.4, 69.8, 68.3, 67.0, 59.6, 59.1, 44.2, 43.6, 36.0, 21.7 ppm;

HRMS (ESI): m/z calcd. for $C_{27}H_{27}N_2O_3$ $[M+H]^+$: 427.2016, found: 427.2015.

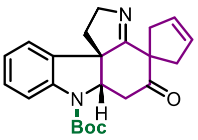

10f, 45%

Compound **10f** was synthesized following general procedure C, brown foam, yield: 45%;

R$_f$ = 0.50 (petroleum ether/ethyl acetate = 1:1);

^{1}H NMR (400 MHz, Methylene Chloride-d_2) δ 7.86 – 7.22 (m, 1H), 7.20 – 7.06 (m, 1H), 6.94 – 6.76 (m, 2H), 5.46 (dt, J = 5.5, 2.6 Hz, 1H), 5.33 (dt, J = 5.9, 2.7 Hz, 1H), 4.54 (s, 1H), 4.10 (ddd, J = 16.1, 9.2, 2.3 Hz, 1H), 3.96 (dt, J = 16.3, 8.1 Hz, 1H), 3.26 (dt, J = 16.8, 2.7 Hz, 1H), 2.82 (dd, J = 13.9, 2.8 Hz, 1H), 2.73 (dd, J = 13.7, 5.9 Hz, 1H), 2.46 (d, J = 16.7 Hz, 1H), 2.38 (ddd, J = 13.1, 7.5, 2.3 Hz, 1H), 2.29 – 2.16 (m, 2H), 1.99 – 1.90 (m, 1H), 1.49 (s, 9H) ppm;

^{13}C NMR (101 MHz, Methylene Chloride-d_2) δ209.4, 180.7, 152.1, 142.0, 133.3, 129.7, 128.4, 127.6, 124.3, 123.6, 116.0, 82.4, 66.0, 61.8, 60.2, 59.4, 54.4, 44.3, 42.9, 28.7 ppm;

HRMS (ESI): m/z calcd. for $C_{23}H_{27}N_2O_3$ $[M+H]^+$: 379.2016, found: 379.2017.

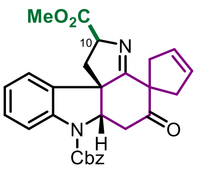

10g, 76%, d.r. > 20:1

Compound **10g** was synthesized following general procedure C; White foam, yield: 76%, d.r. > 20:1.

R$_f$ = 0.50 (petroleum ether/ethyl acetate = 1:1);

^{1}H NMR (400 MHz, Acetonitrile-d_3) δ 7.91 – 7.50 (m, 1H), 7.50 – 7.30 (m, 5H), 7.29 – 7.21 (m, 1H), 7.16 (dd, J = 7.6, 1.3 Hz, 1H), 7.03 (t, J = 7.5 Hz, 1H), 5.41 – 5.54 (m), 5.36 – 5.19 (m, 2H), 5.06 (dd, J = 9.8, 4.4 Hz, 1H), 4.82 (dd, J = 6.3, 1.9 Hz, 1H), 3.76 (s, 3H), 3.35 – 3.45 (m, 1H), 2.95 (dd, J = 13.5, 6.3 Hz, 1H), 2.79 (dd, J = 14.2, 9.9 Hz, 1H), 2.73 – 2.64 (m, 1H), 2.54 – 2.48 (m, 1H), 2.45 (dd, J = 14.2, 4.5 Hz, 1H), 2.28 – 2.12 (m, 2H) ppm;

^{13}C NMR (101 MHz, Acetonitrile-*d_3*) δ 209.2, 182.9, 174.3, 153.4, 143.1, 137.7, 134.1, 130.8, 130.0, 130.0, 129.7, 129.5, 128.0, 127.0, 124.9, 116.1, 74.5, 68.7, 67.0, 64.2, 59.9, 53.4, 45.9, 45.5, 44.3, 43.9 ppm;

HRMS (ESI): *m/z* calcd for $C_{28}H_{27}N_2O_5$ $[M+H]^+$: 471.1915, found: 471.1917.

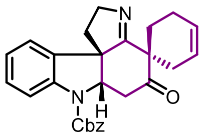

10h-1, 43%

Compound **10h-1** was synthesized following general procedure C; brown foam, yield: 43%. Characterization data was identical with previously reported data [3].

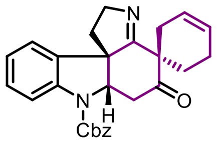

10h-2
43%

Compound **10h-2** was synthesized following general procedure C; brown foam, yield: 43%. Characterization data was identical with previously reported data [3].

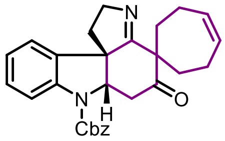

10i
67%

Compound **10i** was synthesized following general procedure C, brown foam, yield: 67%;

R*f* = 0.50 (petroleum ether/ethyl acetate = 1:1);

^{1}H NMR (400 MHz, Acetonitrile-*d_3*) δ 7.75 (s, 1H), 7.50 – 7.31 (m, 5H), 7.21 (q, *J* = 8.1, 7.1 Hz, 1H), 7.03 – 6.92 (m, 2H), 5.64 – 5.48 (m, 2H), 5.35 – 5.20 (m, 2H), 4.70 (dd, *J* = 5.7, 2.7 Hz, 1H), 4.19 – 3.99 (m, 2H), 2.98 (dd, *J* = 13.7, 5.8 Hz, 1H), 2.86 – 2.72 (m, 1H), 2.71 – 2.61 (m, 1H), 2.39 (ddd, *J* = 13.0, 7.1, 2.2 Hz, 1H), 2.35 – 2.30 (m, 1H), 2.30 – 2.25 (m, 1H), 2.24 – 2.17 (m, 1H), 2.12 – 2.08 (m, 1H), 2.06 – 1.98 (m, 1H), 1.80 – 1.66 (m, 2H), 0.78 (dd, *J* = 14.4, 7.5 Hz, 1H) ppm;

^{13}C NMR (101 MHz, Acetonitrile-*d_3*) δ 211.0, 179.1, 153.4, 140.2, 137.4, 134.9, 131.7, 131.4, 130.0, 129.6, 129.2, 129.1, 124.7, 124.5, 116.0, 68.2, 66.3, 62.2, 60.2, 58.2, 43.1, 42.3, 34.4, 32.2, 24.6, 24.2 ppm.

HRMS (ESI): *m/z* calcd. for $C_{28}H_{29}N_2O_3$ $[M+H]^+$: 441.2178, found: 441.2178.

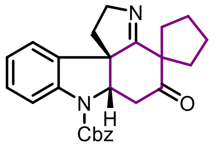

10j 60%

Compound **10j** was synthesized following general procedure C, brown foam, yield: 60%;

R*f* = 0.50 (petroleum ether/ethyl acetate = 1:1);

^{1}H NMR (400 MHz, Acetonitrile-*d_3*) δ 7.68 (d, *J* = 33.4 Hz, 1H), 7.49 – 7.32 (m, 5H), 7.20 (m, 1H), 6.97 (q, *J* = 4.6, 4.1 Hz, 2H), 5.41 – 5.18 (m, 2H), 4.72 (dd, *J* = 5.8, 2.7 Hz, 1H), 4.14 – 3.95 (m, 2H), 2.90 (dd, *J* = 13.7, 5.8 Hz,

1H), 2.78 (dd, J = 13.8, 2.7 Hz, 1H), 2.49 – 2.41 (m, 1H), 2.36 – 2.26 (m, 2H), 1.78 – 1.68 (m, 2H), 1.62 – 1.53 (m, 2H), 1.52 – 1.42 (m, 2H), 1.13 (m, 1H) ppm;

^{13}C NMR (101 MHz, Acetonitrile-d_3) δ 212.1, 181.1, 153.8, 142.7, 137.8, 135.0, 130.4, 130.0, 129.7, 129.5, 125.5, 124.9, 116.3, 68.6, 66.7, 62.4, 60.7, 42.9, 38.4, 38.1, 27.8, 26.5 ppm;

HRMS (ESI): m/z calcd. for $C_{26}H_{27}N_2O_3$ $[M+H]^+$: 415.2016, found: 415.2012.

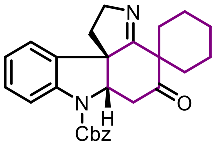

10k, 57%

Compound **10k** was synthesized following general procedure C;

Brown foam, yield: 57% (77% brsm, 24 h), 61% (94% brsm, 48h);

R$_f$ = 0.50 (petroleum ether/ethyl acetate = 1:1);

^{1}H NMR (400 MHz, Acetonitrile-d_3) δ 7.72 (s, 1H), 7.52 – 7.33 (m, 5H), 7.23 (t, J = 7.8 Hz, 1H), 7.04 – 6.89 (m, 2H), 5.36 – 5.20 (m, 2H), 4.68 (dd, J = 5.8, 3.0 Hz, 1H), 4.12 – 4.02 (m, 2H), 2.91 (dd, J = 13.7, 5.8 Hz, 1H), 2.79 (dd, J = 13.7, 3.0 Hz, 1H), 2.39 – 2.31 (m, 1H), 2.31 – 2.22 (m, 1H), 2.12 – 1.99 (m, 2H), 1.54 (m, 1H), 1.44 – 1.23 (m, 6H), 0.92 (m, 1H) ppm;

^{13}C NMR (101 MHz, Acetonitrile-d_3) δ 212.7, 180.1, 153.9, 142.4, 137.8, 135.3, 130.4, 130.0, 129.7, 129.5, 125.3, 124.8, 116.4, 68.7, 66.9, 63.0, 60.7, 56.5, 43.1, 42.9, 34.2, 34.1, 26.5, 23.2, 22.8 ppm;

HRMS (ESI): m/z calcd. for $C_{27}H_{29}N_2O_3$ $[M+H]^+$: 429.2173, found: 429.2172.

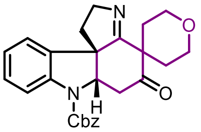

10l, 66%

Compound **10l** was synthesized following general procedure C;

Brown foam, yield: 66% (74% brsm);

R$_f$ = 0.40 (petroleum ether/ethyl acetate = 1:1);

^{1}H NMR (400 MHz, Acetonitrile-d_3) δ 7.70 (s, 1H), 7.52 – 7.26 (m, 5H), 7.22 (t, J = 7.7 Hz, 1H), 7.04 – 6.93 (m, 2H), 5.28 (q, J = 12.5 Hz, 2H), 4.70 (dd, J = 5.7, 3.1 Hz, 1H), 4.13 – 4.07 (m, 2H), 4.05 (m, 1H), 3.53 (m, 2H), 3.41 (m, 1H), 2.93 (dd, J = 13.9, 5.7 Hz, 1H), 2.81 (dd, J = 13.8, 3.1 Hz, 1H), 2.44 – 2.22 (m, 2H), 2.13 – 2.10 (m, 1H), 1.63 (m, 1H), 1.40 (m, 1H), 0.97 (d, J = 14.1 Hz, 1H) ppm;

^{13}C NMR (101 MHz, Acetonitrile-d_3) δ 211.6, 179.2, 153.8, 142.4, 137.7, 134.9, 130.5, 129.9, 129.6, 129.5, 125.2, 124.9, 116.5, 68.6, 66.7, 64.8, 64.1, 62.9, 60.7, 53.6, 43.0, 42.9, 33.9, 33.1 ppm;

HRMS (ESI): m/z calcd. for $C_{27}H_{29}N_2O_3$ $[M+H]^+$: 429.2173, found: 429.2172.

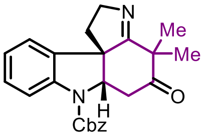

10m, 48h, 62%

Compound **10m** was synthesized following general procedure C;

Brown foam, yield: 50% (24 h), 62% (48 h);

R$_f$ = 0.50 (petroleum ether/ethyl acetate = 1:1);

^{1}H NMR (400 MHz, Acetonitrile-d_3) δ 7.82 – 7.51 (m, 1H), 7.49 – 7.32 (m, 5H), 7.23 (d, J = 8.2 Hz, 1H), 7.02 – 6.94 (m, 2H), 5.28 (q, J = 12.6 Hz, 2H), 4.73 (dd, J = 5.8, 2.6 Hz, 1H), 4.11 (m, 1H), 4.01 (m, 1H), 2.95 (dd, J = 13.9, 5.8 Hz, 1H), 2.85 – 2.76 (m, 1H), 2.45 – 2.34 (m, 1H), 2.34 – 2.22 (m, 1H), 1.30 (s, 3H), 0.73 (s, 3H) ppm;

^{13}C NMR (101 MHz, Acetonitrile-d_3) δ 212.9, 181.1, 153.8, 142.6, 137.8, 135.1, 130.5, 130.0, 129.7, 129.5, 125.5, 124.9, 116.4, 68.7, 66.9, 62.5, 60.7, 51.9, 43.2, 42.6, 26.0, 25.3 ppm;

HRMS (ESI): *m/z* calcd. for $C_{24}H_{24}N_2O_3Na$ $[M+Na]^+$: 411.1679, found: 411.1679.

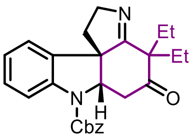

10n 48h, 58%(72% brsm)

Compound **10n** was synthesized following general procedure C;

Brown foam, yield: 44% (85% brsm, 24 h), 58% (72% brsm, 48h);

R_f = 0.50 (petroleum ether/ethyl acetate = 1:1);

^{1}H NMR (400 MHz, Acetonitrile-d_3) δ 7.68 (s, 1H), 7.50 – 7.31 (m, 5H), 7.21 (s, 1H), 7.05 (dd, J = 7.5, 1.4 Hz, 1H), 6.96 (dd, J = 7.4, 1.1 Hz, 1H), 5.40 – 5.14 (m, 2H), 4.69 (dd, J = 5.3, 2.7 Hz, 1H), 4.19 – 3.99 (m, 2H), 2.95 – 2.85 (m, 1H), 2.80 (dd, J = 13.8, 5.4 Hz, 1H), 2.43 (dd, J = 6.9, 2.5 Hz, 1H), 2.33 (d, J = 13.2 Hz, 1H), 1.85 (q, J = 7.4 Hz, 2H), 1.56 (m, 1H), 0.70 (t, J = 7.5 Hz, 3H), 0.56 (t, J = 7.4 Hz, 3H), 0.31 (d, J = 7.1 Hz, 1H) ppm;

^{13}C NMR (101 MHz, Acetonitrile-d_3) δ 212.6, 177.5, 154.0, 142.5, 137.8, 135.3, 130.4, 130.0, 129.7, 129.5, 124.9, 124.8, 116.4, 68.7, 66.7, 62.5, 60.7, 59.6, 44.5, 43.4, 27.8, 26.4, 9.2, 8.0 ppm;

HRMS (ESI): *m/z* calcd. for $C_{26}H_{29}N_2O_3$ $[M+H]^+$: 417.2173, found: 417.2173.

II: Computational details

The calculations were performed with the Gaussian 16 program package [3]. The geometry optimizations and two-dimension scans were performed using B3LYP functional [6] with 6-31G(d) basis set [7-10] for all atoms. Higher level of single point electronic energies for those structures were calculated at B3LYP/def2-TZVP level [11]. Dispersion corrections using DFT-D3 method [12] were applied in single point electronic energies calculations and solvation effect using SMD (MeCN) method [13] were applied in all calculations. Vibrational harmonic frequencies and thermal corrections were calculated using the same level as the optimization. An IRC [14], analysis was performed to confirm that all the stationary points were smoothly connected to each other. 3D structures were rendered by using CYLview [16]; spin density and NBO orbitals were rendered by using VMD [17].

Table S3-1. Calculated imaginary frequencies of all transition states species

Species	**Frequencies**
TS1	-287.38
TS2	-1077.06

Table S3-2. Calculated energy values (in Hartee)

**Species**	**E_0**	**E**	**H**	**G**	**SPE**	**$G_{(sol, MeCN)}$**
**IN1**	-1032.605777	-1032.583962	-1032.583018	-1032.658150	-1033.396682	-1033.096066
**TS1**	-1032.594947	-1032.573982	-1032.573038	-1032.645222	-1033.385736	-1033.082868
**IN2**	-1032.610661	-1032.589827	-1032.588883	-1032.660028	-1033.400483	-1033.095150
**TS2**	-1032.576069	-1032.555685	-1032.554740	-1032.625183	-1033.363619	-1033.060349
**IN3**	-1032.627819	-1032.607639	-1032.606695	-1032.676303	-1033.421594	-1033.113409
**IN4**	-1032.568780	-1032.548460	-1032.547516	-1032.617163	-1033.358297	-1033.052428

E_0 = Sum of electronic and zero-point Energies

E = Sum of electronic and thermal Energies

H = Sum of electronic and thermal Enthalpies

G = Sum of electronic and thermal Free Energies

SPE = Single point energies

$G_{(sol, MeCN)}$= Solvated Gibbs free energies

Two-dimension scans of simplified structures

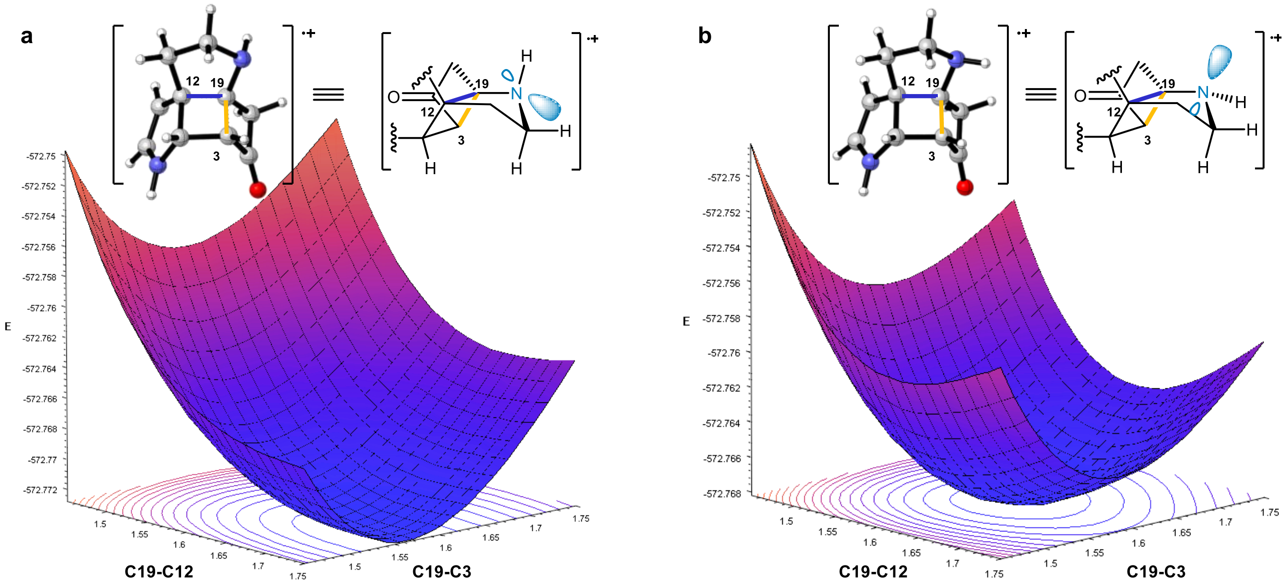

Figure S3-1. **Two-dimension scans of simplified structures.**

Two-dimension scans (C19–C12 and C19–C3 from 1.45 to 1.75Å) were performed by using simplified structures bearing sp^3-hybridized N atom with different conformation to find a minimum on PES. As shown in Figure S3-1a, no minimum was found when the nonbonding sp^3 orbital in N atom takes the *gauche* position related to the adjacent C19–C3 bond. When the nonbonding sp^3 orbital in N atom is in a position antiperiplanar to the adjacent C19–C3 bond, a minimum was found located on the PES in this conformation. Based on the 2D-scan results depicted in Figure S3-1b, the attempts to locate an intermediate with bicyclo[2.2.0]hexane unit was succeed (**IN4**).

Cartesian coordinates

IN1 (Charge = +1; Multiplicity = 2)

O	2.295698	-2.481132	-0.784135
C	2.588790	-1.434322	-0.015446
O	3.409849	-1.401598	0.875016
N	1.796811	-0.325630	-0.374174
C	1.831629	0.946084	0.245549
C	2.595127	1.419458	1.302940
H	3.315710	0.791955	1.809481
C	2.393557	2.753614	1.686793
H	2.976826	3.151639	2.511914
C	1.465225	3.582405	1.035548
H	1.343215	4.610130	1.363503
C	0.703846	3.099178	-0.023261
H	-0.011188	3.737931	-0.531923
C	0.883893	1.762303	-0.429509
C	0.278924	0.971156	-1.460833
C	-0.774985	1.363828	-2.440051
C	-2.095935	1.914531	-1.822287
H	-2.890028	1.785767	-2.565506
H	-2.006216	2.979361	-1.606093
N	-2.504191	1.286131	-0.565959
H	-2.820159	1.915018	0.168593
H	-0.403293	2.170518	-3.084481
H	-0.994605	0.514304	-3.090343
C	0.858587	-0.294127	-1.381516
H	0.701850	-1.152745	-2.015156
C	-2.014111	-1.153599	-0.938719
H	-1.757033	-1.379200	-1.965873
C	-2.432487	0.009370	-0.263943
C	-2.273521	-1.961994	0.256375
O	-2.168925	-3.138591	0.550530
C	-2.772649	-0.705816	1.051234
C	-4.268184	-0.732613	1.388286
C	-1.916717	-0.320796	2.261749
H	-4.452712	-1.470291	2.177085
H	-4.874647	-1.001381	0.516435
H	-2.085265	-1.038750	3.072066
H	-0.848495	-0.319926	2.020966
H	-2.191177	0.675334	2.628478
H	-4.599868	0.246343	1.753362
C	3.048960	-3.688363	-0.509586
H	2.669439	-4.424209	-1.217479
H	4.114165	-3.510998	-0.672579
H	2.872474	-4.015528	0.517252

IN2

Charge = +1; Multiplicity = 2

C	-3.806950	-1.208348	1.371566
C	-3.128385	-0.051538	0.951233
C	-2.009481	-0.213449	0.146569
C	-1.545401	-1.508288	-0.247444
C	-2.252597	-2.654063	0.187942
C	-3.374378	-2.490389	0.993775
N	-1.162110	0.751539	-0.427657
C	-0.068554	0.102759	-1.175044
C	-0.404057	-1.367468	-1.058997
C	2.279980	-0.647325	-0.199201
C	1.342081	0.462438	-0.581298
C	1.572429	0.976261	0.868600
N	2.509338	-1.762922	-0.783312
C	1.822285	-2.156931	-2.034168
C	0.338401	-2.459026	-1.769945
O	1.060935	1.830425	1.541064
O	-0.417636	2.741836	-1.125150
C	-1.356198	2.114789	-0.386644
O	-2.251458	2.675693	0.221274
H	-4.685598	-1.101955	2.001446
H	-3.468241	0.934086	1.238976
H	-1.918807	-3.644500	-0.107496
H	-3.923891	-3.363608	1.334559
H	-0.077972	0.445118	-2.215645
H	1.865476	1.157415	-1.255311
H	1.942145	-1.335608	-2.745600
H	2.337387	-3.034913	-2.423004
H	-0.110358	-2.656981	-2.756477
H	0.251355	-3.391690	-1.200633
H	3.134047	-2.439089	-0.339345
C	2.694160	-0.064172	1.140295
C	4.114630	0.531413	1.156206
C	2.424757	-0.971047	2.350237
H	4.232548	1.132951	2.062828
H	4.299702	1.168765	0.286138
H	3.149294	-1.791689	2.372976
H	1.414686	-1.391562	2.324210
H	2.534914	-0.380528	3.265100
H	4.854867	-0.274838	1.168506
C	-0.473227	4.181884	-1.099908
H	0.344636	4.511795	-1.741145
H	-1.429216	4.537039	-1.492111
H	-0.329276	4.550855	-0.081343

IN3

Charge = +1; Multiplicity = 2

C	-1.299499	3.407789	-1.282047
C	-1.944202	2.198155	-1.001490
C	-1.226446	1.221571	-0.308201
C	0.095670	1.450184	0.097327
C	0.727016	2.656368	-0.186898
C	0.020080	3.641655	-0.885634
N	-1.636904	-0.065129	0.093406
C	-0.475746	-0.834271	0.622402
C	0.598553	0.258263	0.915310
C	1.993912	-0.180468	0.518020
C	-0.056380	-1.874069	-0.371803
C	1.231259	-2.004153	-0.984222
N	2.847794	0.190160	1.412462
C	2.280520	0.898089	2.577451
C	0.784100	0.593675	2.430855
O	1.461596	-2.932521	-1.772464
O	-2.985360	-1.778501	0.601671
C	-2.931513	-0.529868	0.092093
O	-3.896811	0.095576	-0.314000
H	-1.842784	4.177156	-1.823842
H	-2.966598	2.020823	-1.305845
H	1.752765	2.831567	0.125640
H	0.500261	4.587469	-1.118132
H	-0.773352	-1.332381	1.549713
H	-0.799003	-2.612707	-0.656661
H	2.729315	0.498167	3.488114
H	2.528841	1.959519	2.491466
H	0.527419	-0.285413	3.028996
H	0.155084	1.427603	2.743711
H	3.849878	0.021648	1.328171
C	2.351010	-0.960977	-0.710658
C	3.709575	-1.672367	-0.570517
C	2.390749	-0.015334	-1.951367
H	3.855985	-2.317595	-1.438630
H	3.749810	-2.292520	0.331312
H	3.147759	0.761389	-1.807844
H	1.425134	0.457118	-2.142263
H	2.663593	-0.620781	-2.820668
H	4.531140	-0.948309	-0.545479
C	-4.300290	-2.370104	0.649796
H	-4.151726	-3.361378	1.078568
H	-4.964246	-1.777613	1.283899
H	-4.721110	-2.450219	-0.355469

IN4

Charge = +1; Multiplicity = 2

C	1.252957	3.608388	0.665361
C	1.848650	2.386738	0.438773
C	1.039945	1.371354	-0.129080
C	-0.352575	1.595044	-0.439225
C	-0.914644	2.840025	-0.201878
C	-0.112568	3.842956	0.346356
N	1.384565	0.096239	-0.474294
C	0.258348	-0.622271	-1.101745
C	-0.931348	0.361200	-1.030939
C	-1.768986	-0.741017	-0.175339
C	-0.488579	-1.686823	-0.256408
C	-0.369587	-1.490515	1.262351
N	-3.003696	-0.874284	-0.879030
C	-2.725698	-0.676806	-2.307339
C	-1.793600	0.541653	-2.298878
O	0.525928	-1.712478	2.043842
O	2.639235	-1.735948	-0.751798
C	2.646900	-0.515518	-0.234982
O	3.555445	0.026516	0.351660
H	1.838848	4.416267	1.091616
H	2.887458	2.204553	0.672534
H	-1.957012	3.030131	-0.434651
H	-0.534916	4.824971	0.534958
H	0.551043	-0.972001	-2.093370
H	-0.478566	-2.708270	-0.640094
H	-2.218430	-1.543153	-2.761942
H	-3.652386	-0.494315	-2.859296
H	-1.177325	0.621221	-3.198608
H	-2.379386	1.460270	-2.197476
H	-3.475910	-1.754986	-0.685611
C	-1.745060	-0.819570	1.392699
C	-2.760229	-1.892910	1.857851
C	-1.854005	0.424688	2.268752
H	-2.574937	-2.136267	2.909000
H	-2.690695	-2.819696	1.276926
H	-2.806397	0.936301	2.087948
H	-1.042300	1.134208	2.089070
H	-3.779220	-1.501827	1.765109
H	-1.819159	0.139204	3.326176
C	3.851921	-2.509974	-0.556578
H	3.664200	-3.457211	-1.059893
H	4.700468	-1.992319	-1.008057
H	4.022610	-2.665336	0.510781

TS1

Charge = +1; Multiplicity = 2

O	2.243416	1.961294	-1.065729
C	1.267499	2.227164	-0.194486

O	1.307566	3.053949	0.693644
N	0.160197	1.409033	-0.443480
C	-1.093254	1.504042	0.189727
C	-1.513535	2.301994	1.249118
H	-0.838319	2.992630	1.735768
C	-2.847779	2.180091	1.651258
H	-3.204834	2.795955	2.471630
C	-3.736887	1.284581	1.023899
H	-4.764603	1.224353	1.369395
C	-3.312663	0.481157	-0.024179
H	-3.993594	-0.209973	-0.511319
C	-1.972635	0.585553	-0.458782
C	-1.243121	-0.078724	-1.478918
C	-1.627029	-1.277722	-2.288104
C	-1.873039	-2.533118	-1.415954
H	-1.789585	-3.436945	-2.028207
H	-2.877840	-2.506803	-0.990302
N	-0.969860	-2.663897	-0.252067
H	-1.364752	-3.172731	0.538230
H	-2.557633	-1.100485	-2.839395
H	-0.849145	-1.479994	-3.028848
C	0.107922	0.393455	-1.416104
H	0.748147	0.513703	-2.278356
C	1.077637	-1.341606	-0.907128
H	1.335443	-1.543512	-1.942582
C	0.219635	-2.166062	-0.087584
C	2.029865	-1.248483	0.243008
O	3.091546	-0.699160	0.440588
C	1.119599	-2.145209	1.145413
C	1.762671	-3.505049	1.464488
C	0.541190	-1.477410	2.399076
H	2.579051	-3.358977	2.179643
H	2.169541	-3.983525	0.567264
H	1.344846	-1.320743	3.126749
H	0.086669	-0.507907	2.171796
H	-0.217645	-2.119788	2.860178
H	1.024662	-4.178230	1.914533
C	3.482827	2.672855	-0.852606
H	3.887710	2.437858	0.133966
H	4.152918	2.316263	-1.634365
H	3.322948	3.749179	-0.947829

TS2

Charge = +1; Multiplicity = 2

C	1.098235	3.625736	0.730339
C	1.758139	2.417625	0.496906
C	1.028619	1.386400	-0.100008
C	-0.330456	1.565000	-0.455682
C	-0.970792	2.783420	-0.211109
C	-0.249056	3.817161	0.379568
N	1.449695	0.094735	-0.452255
C	0.354870	-0.641742	-1.097089
C	-0.846200	0.333905	-1.101517
C	-1.776925	-0.743893	-0.188813
C	-0.431371	-1.656668	-0.246762
C	-0.409821	-1.479655	1.281849
N	-2.885707	-0.968594	-0.958469
C	-2.719824	-0.665874	-2.372815
C	-1.685887	0.477590	-2.383607
O	0.451599	-1.711829	2.091649
O	2.733528	-1.714315	-0.672402
C	2.696581	-0.447339	-0.218727
O	3.623765	0.132950	0.318466
H	1.647466	4.438962	1.196542
H	2.795646	2.279127	0.767719
H	-2.014217	2.917493	-0.481390
H	-0.726218	4.773416	0.570719
H	0.660839	-1.032320	-2.067545
H	-0.509249	-2.676451	-0.628883
H	-2.342358	-1.560767	-2.887405
H	-3.683127	-0.397147	-2.809267
H	-1.051987	0.453325	-3.273521
H	-2.207128	1.438541	-2.359881
H	-3.797542	-1.180882	-0.559803
C	-1.812794	-0.863566	1.370035
C	-2.808347	-1.971026	1.792835
C	-1.996410	0.377809	2.243387
H	-2.622535	-2.235567	2.838231
H	-2.712082	-2.877477	1.185672
H	-2.974424	0.832960	2.052670
H	-1.221982	1.126889	2.069322
H	-1.959015	0.084072	3.297872
H	-3.836760	-1.600320	1.712404
C	3.983964	-2.406706	-0.475744
H	3.832463	-3.397714	-0.904098
H	4.794238	-1.888283	-0.994062
H	4.213758	-2.484357	0.589770

R-50

Charge = +1; Multiplicity = 2

C	0.815369	3.697286	0.775822
C	1.524858	2.507872	0.539717
C	0.891153	1.510644	-0.187764
C	-0.415660	1.699220	-0.742718

C	-1.114390	2.897385	-0.465917
C	-0.495103	3.877683	0.301244
N	1.330810	0.215645	-0.526991
C	0.238668	-0.526010	-1.177539
C	-0.762570	0.560818	-1.497262
C	-1.909198	-1.318368	0.114624
C	-0.453481	-1.568779	-0.197405
C	-0.251536	-1.536139	1.344157
N	-2.898549	-1.134840	-0.679050
C	-2.689983	-0.974808	-2.130758
C	-1.949251	0.357753	-2.385444
O	0.712707	-1.635753	2.053784
O	2.716798	-1.527194	-0.802118
C	2.613792	-0.260273	-0.355883
O	3.531687	0.383031	0.124452
H	1.290059	4.485384	1.353402
H	2.526789	2.366905	0.922511
H	-2.120004	3.042655	-0.850495
H	-1.024612	4.799180	0.526949
H	0.610366	-1.068467	-2.047787
H	-0.302162	-2.593589	-0.565587
H	-2.107672	-1.828984	-2.481221
H	-3.664893	-0.988119	-2.613911
H	-1.645802	0.345731	-3.442730
H	-2.646888	1.192924	-2.263522
H	-3.824435	-0.944203	-0.290701
C	-1.766947	-1.304462	1.621121
C	-2.535053	-2.411094	2.354753
C	-2.019794	0.082370	2.254376
H	-2.206991	-2.445533	3.398703
H	-2.366250	-3.392753	1.900612
H	-3.089356	0.315706	2.217338
H	-1.466202	0.876617	1.750118
H	-1.702430	0.042013	3.300151
H	-3.607720	-2.191646	2.332955
C	4.031989	-2.110673	-0.688003
H	3.933067	-3.119729	-1.089002
H	4.756738	-1.538958	-1.272883
H	4.345500	-2.146661	0.358083

R-24

Charge = +1; Multiplicity = 2

C	0.817667	3.703786	0.668345
C	1.554030	2.538359	0.419359
C	0.886651	1.468285	-0.166982
C	-0.479846	1.588796	-0.563843
C	-1.210256	2.755848	-0.260008

C	-0.552478	3.805773	0.361116
N	1.352153	0.190746	-0.519185
C	0.265799	-0.588475	-1.139735
C	-0.853061	0.432216	-1.282872
C	-1.855821	-1.157450	0.027912
C	-0.420502	-1.646392	-0.222342
C	-0.252489	-1.510990	1.318469
N	-2.922600	-1.200596	-0.729926
C	-2.736941	-0.995999	-2.167939
C	-1.936677	0.327302	-2.305421
O	0.684580	-1.648668	2.059149
O	2.763232	-1.522686	-0.806772
C	2.650081	-0.258530	-0.362641
O	3.559662	0.403454	0.105994
H	1.322428	4.549583	1.126265
H	2.602199	2.470839	0.675634
H	-2.261645	2.823828	-0.522666
H	-1.089259	4.718894	0.600374
H	0.601412	-1.037606	-2.076553
H	-0.327059	-2.675730	-0.587903
H	-2.175983	-1.841336	-2.576742
H	-3.711477	-0.943778	-2.653108
H	-1.502018	0.366807	-3.313232
H	-2.613568	1.180458	-2.202512
H	-3.809157	-0.908605	-0.318339
C	-1.730718	-1.103050	1.538584
C	-2.583164	-2.248991	2.119454
C	-1.953989	0.206254	2.310146
H	-2.322361	-2.403598	3.170809
H	-2.428359	-3.187584	1.577495
H	-2.988006	0.546231	2.189806
H	-1.280864	1.001182	1.983880
H	-1.775461	0.014414	3.373123
H	-3.642572	-1.978885	2.053870
C	4.078934	-2.105255	-0.686927
H	3.982231	-3.113704	-1.089600
H	4.805683	-1.531795	-1.267401
H	4.385683	-2.142111	0.361144

F-10

Charge = +1; Multiplicity = 2

C	0.802926	3.708623	0.623789
C	1.536546	2.544852	0.361809
C	0.853550	1.453585	-0.175728
C	-0.524005	1.519507	-0.439032
C	-1.238168	2.683992	-0.181838
C	-0.565955	3.788106	0.354907

N	1.359250	0.181596	-0.538552
C	0.289702	-0.655116	-1.094150
C	-1.009943	0.208960	-1.013036
C	-1.797256	-0.810690	-0.091454
C	-0.268684	-1.748310	-0.181495
C	-0.256402	-1.505228	1.320981
N	-2.866919	-1.250346	-0.788053
C	-2.868054	-0.892461	-2.206017
C	-1.890818	0.291339	-2.281429
O	0.651037	-1.655932	2.100770
O	2.771141	-1.524354	-0.812198
C	2.653820	-0.252577	-0.375741
O	3.565073	0.408486	0.094061
H	1.319130	4.568180	1.042663
H	2.597245	2.489748	0.565071
H	-2.304133	2.732172	-0.387562
H	-1.110448	4.704076	0.564351
H	0.543747	-1.027624	-2.086464
H	-0.339590	-2.772414	-0.548290
H	-2.536582	-1.768046	-2.778309
H	-3.885907	-0.648074	-2.518160
H	-1.286511	0.268443	-3.190475
H	-2.449540	1.230585	-2.264475
H	-3.523155	-1.934669	-0.418438
C	-1.686486	-0.987116	1.467387
C	-2.574243	-2.158772	1.952085
C	-1.913150	0.245659	2.348445
H	-2.306541	-2.393074	2.986333
H	-2.443640	-3.066643	1.352760
H	-2.940712	0.605359	2.230840
H	-1.224779	1.058526	2.114320
H	-1.767072	-0.041210	3.395176
H	-3.629102	-1.863980	1.927729
C	4.085852	-2.103013	-0.684692
H	3.992610	-3.115342	-1.078906
H	4.813998	-1.534132	-1.268269
H	4.391999	-2.131160	0.364029

F-40

Charge = +1; Multiplicity = 2

C	0.801381	3.705618	0.603481
C	1.529233	2.541739	0.335626
C	0.840511	1.447271	-0.195300
C	-0.539450	1.507112	-0.428599
C	-1.248249	2.677899	-0.177032
C	-0.569857	3.785693	0.343629
N	1.345463	0.187111	-0.575854
C	0.247932	-0.719329	-1.041340
C	-1.058950	0.181099	-0.961077
C	-2.006563	-0.611881	-0.061767
C	0.194073	-1.890326	-0.096195
C	-0.241314	-1.551886	1.263420
N	-2.946633	-1.141081	-0.765233
C	-2.892376	-0.861145	-2.214170
C	-1.870636	0.280133	-2.282514
O	0.476057	-1.707752	2.238512
O	2.784192	-1.507718	-0.843066
C	2.643652	-0.241738	-0.399633
O	3.544930	0.424169	0.081296
H	1.322861	4.565765	1.014546
H	2.592344	2.486602	0.523946
H	-2.317197	2.724546	-0.366850
H	-1.112401	4.702987	0.552081
H	0.432080	-1.037911	-2.068148
H	0.797408	-2.762918	-0.268022
H	-2.564256	-1.779297	-2.710906
H	-3.887568	-0.595498	-2.574956
H	-1.226671	0.214564	-3.161275
H	-2.390605	1.241370	-2.305978
H	-3.628940	-1.793759	-0.380130
C	-1.693397	-0.985273	1.363703
C	-2.566274	-2.155561	1.875783
C	-1.805422	0.204192	2.341017
H	-2.231538	-2.427713	2.880077
H	-2.486266	-3.040955	1.235547
H	-2.837252	0.568539	2.370079
H	-1.143393	1.028974	2.075932
H	-1.531279	-0.151859	3.339001
H	-3.617577	-1.853013	1.937524
C	4.092888	-2.089230	-0.683733
H	4.001366	-3.107223	-1.063375
H	4.832851	-1.533158	-1.264872
H	4.381602	-2.101177	0.370120

Simplified structure in Figure 3-1a

C19-C12 = 1.45Å

C19-C3 = 1.45Å

Charge = +1; Multiplicity = 2

N	-1.353341	1.643197	0.684306
C	-1.207724	2.079846	-0.568832
C	-0.060033	1.543446	-1.151927
C	0.640277	0.669948	-0.169684
C	2.162735	0.924970	-0.012546
C	2.668643	-0.451359	0.475333

H	2.577284	-0.523546	1.566114
H	3.708405	-0.650129	0.205233
N	1.768929	-1.435630	-0.151433
H	2.080955	-1.599949	-1.109600
H	2.581664	1.157566	-0.997933
H	2.381036	1.754906	0.665258
C	-0.269834	0.768450	1.092905
H	0.158084	1.086948	2.044391
C	-0.423942	-0.791695	0.938636
H	-0.223278	-1.364163	1.847652
C	0.467470	-0.769292	-0.204775
C	-0.598210	-1.427696	-1.140433
C	-1.568917	-1.292358	0.036396
O	-2.749053	-1.474499	0.198272
H	0.248602	1.718020	-2.175406
H	-1.935790	2.750200	-1.009512
H	-2.129374	1.911113	1.287208
H	-0.871082	-0.941920	-2.082402
H	-0.355987	-2.481594	-1.327722

Simplified structure in Figure 3-1b

C19-C12 = 1.45Å

C19-C3 = 1.45Å

Charge = +1; Multiplicity = 2

N	-1.322873	1.643250	0.719364
C	-1.202585	2.092478	-0.532022
C	-0.076147	1.547744	-1.147795
C	0.642166	0.660560	-0.184568
C	2.169026	0.918446	-0.048007
C	2.682894	-0.440487	0.444286
H	2.593075	-0.509298	1.540703
H	3.729425	-0.617839	0.177311
N	1.794796	-1.371097	-0.269449
H	1.798912	-2.304280	0.141111
H	2.575977	1.147076	-1.037978
H	2.389392	1.750170	0.625076
C	-0.243279	0.749940	1.095227
H	0.205246	1.050333	2.043125
C	-0.422184	-0.805776	0.925520
H	-0.232092	-1.387926	1.831370
C	0.461433	-0.777602	-0.223801
C	-0.622481	-1.419531	-1.157575
C	-1.581739	-1.278830	0.026370
O	-2.764166	-1.441630	0.196919
H	0.213837	1.731627	-2.175062
H	-1.933612	2.775329	-0.947779
H	-2.081638	1.915135	1.343194
H	-0.897836	-0.929004	-2.097139
H	-0.393437	-2.475006	-1.354493

III: ^{1}H NMR and ^{13}C NMR spectra

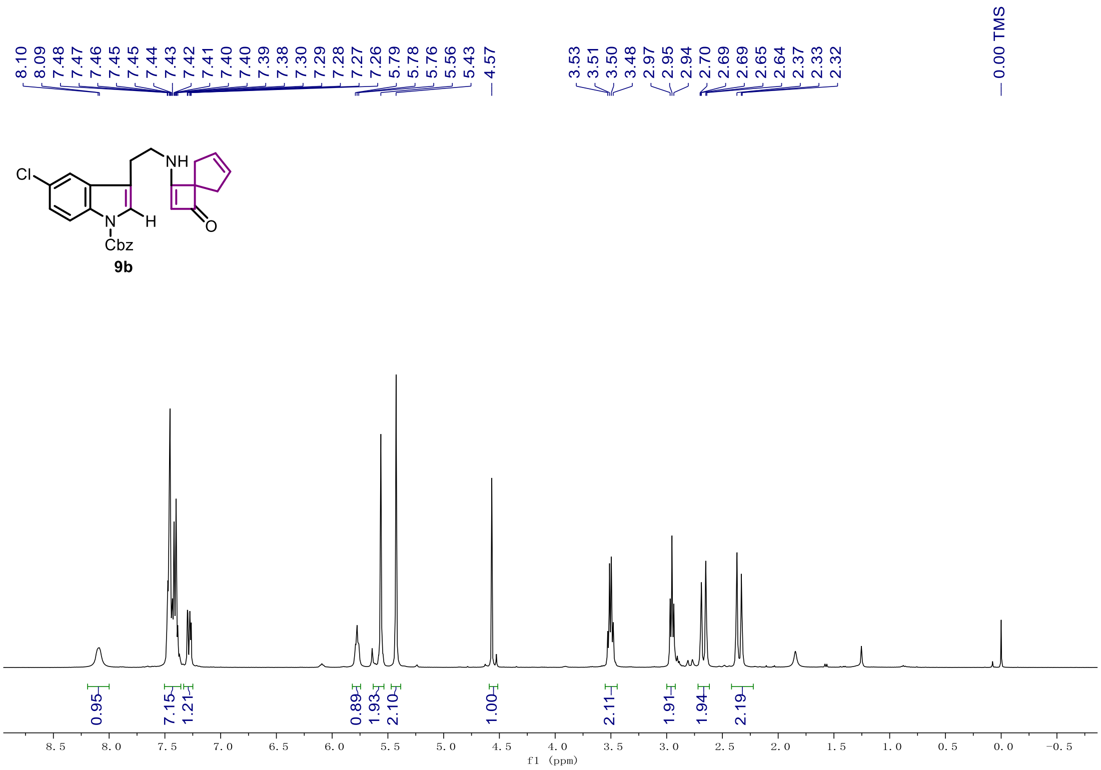

^{1}H NMR spectra of **9b** (400 MHz, Chloroform-*d*)

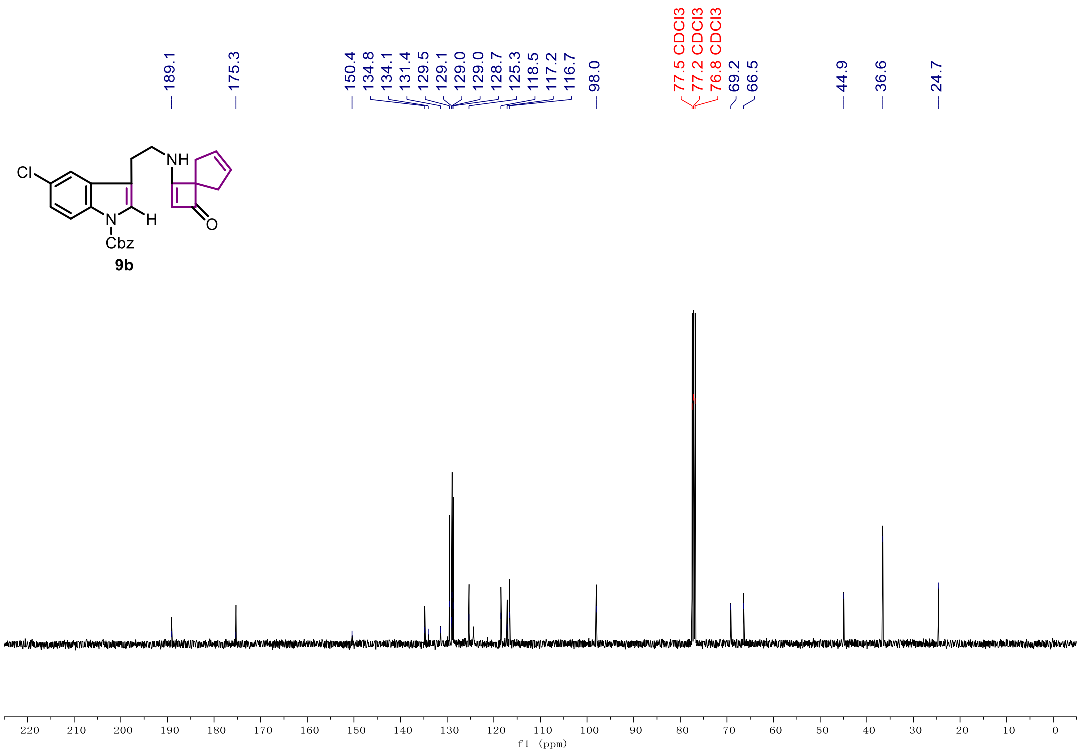

^{13}C NMR spectra of **9b** (101 MHz, Chloroform-*d*)

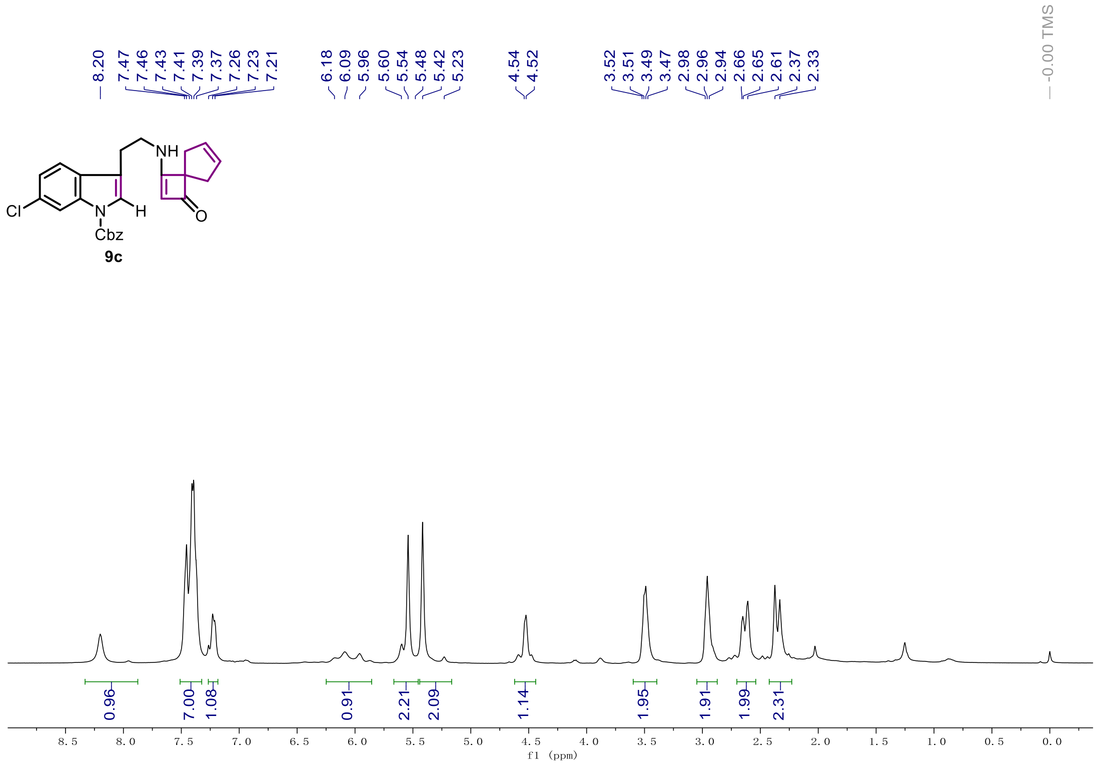

^{1}H NMR spectra of **9c** (400 MHz, Chloroform-*d*)

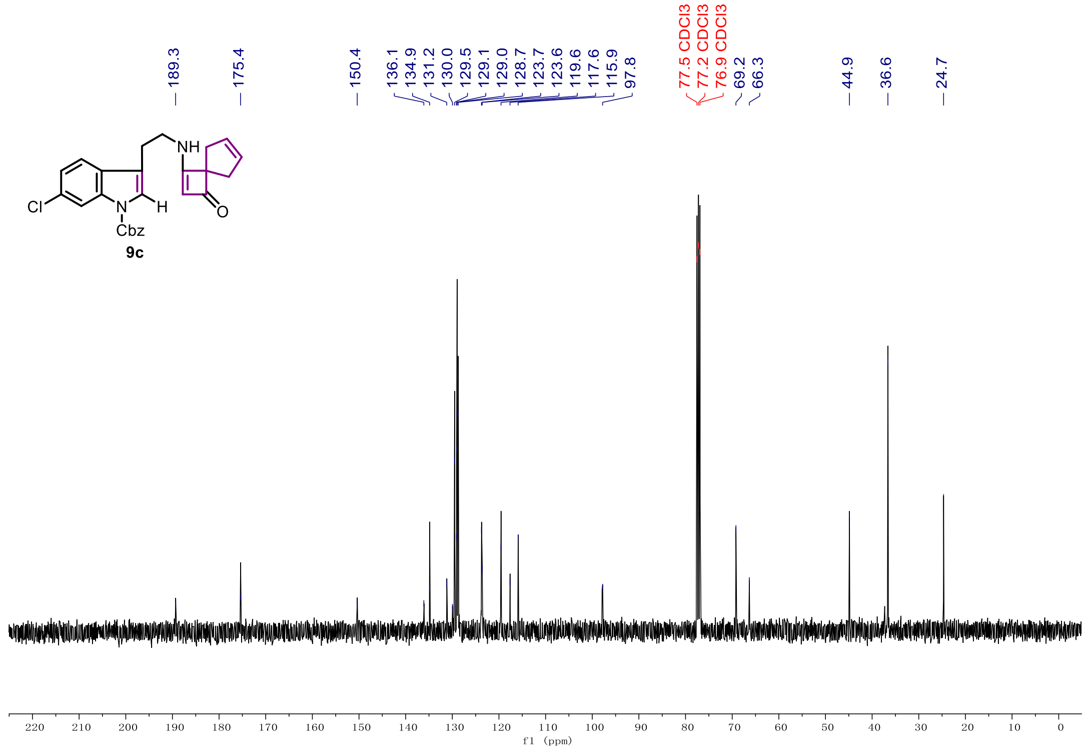

^{13}C NMR spectra of **9c** (101 MHz, Chloroform-*d*)

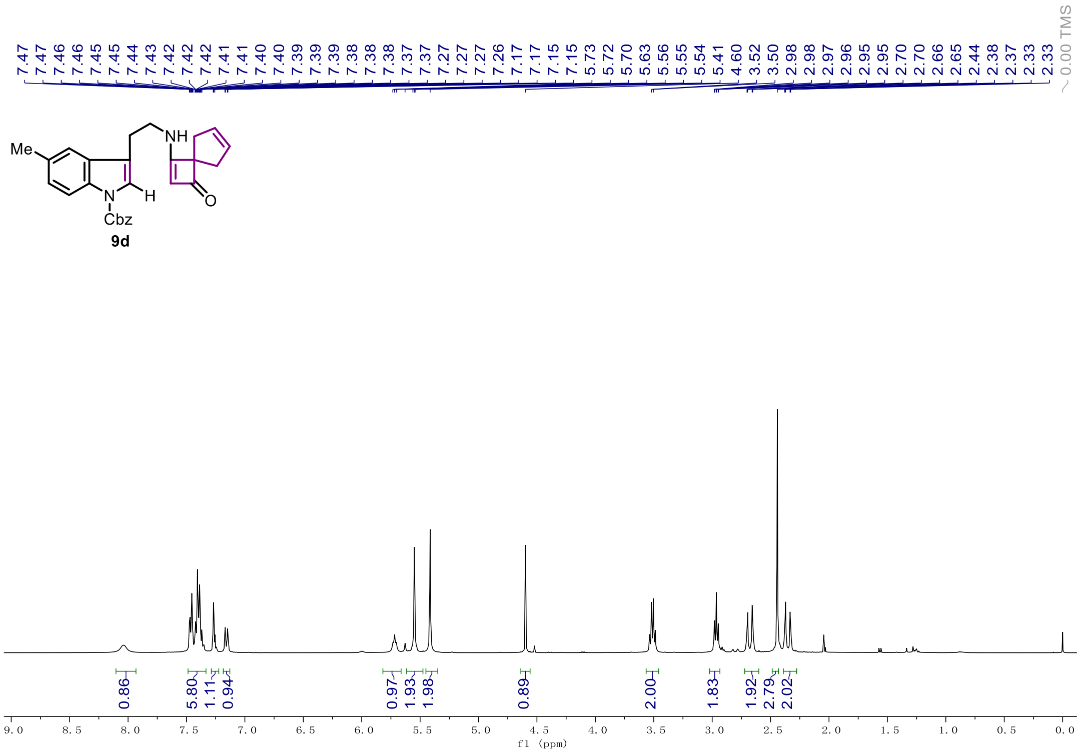

^{1}H NMR spectra of **9d** (400 MHz, Chloroform-*d*)

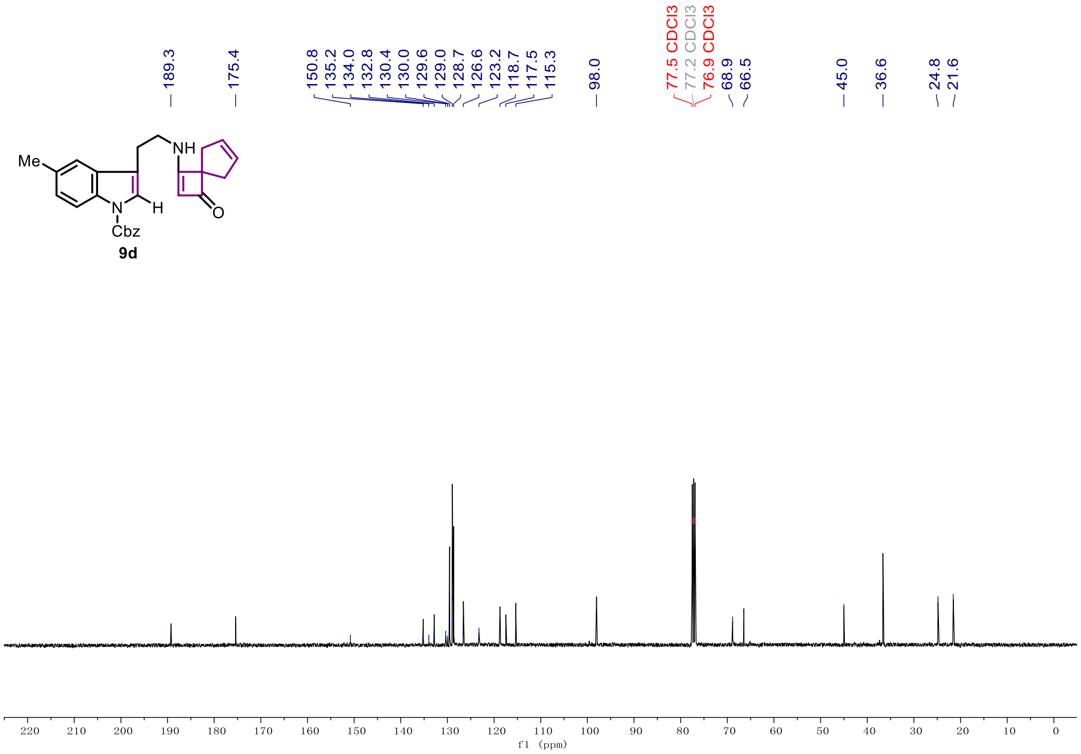

^{13}C NMR spectra of **9d** (101 MHz, Chloroform-*d*)

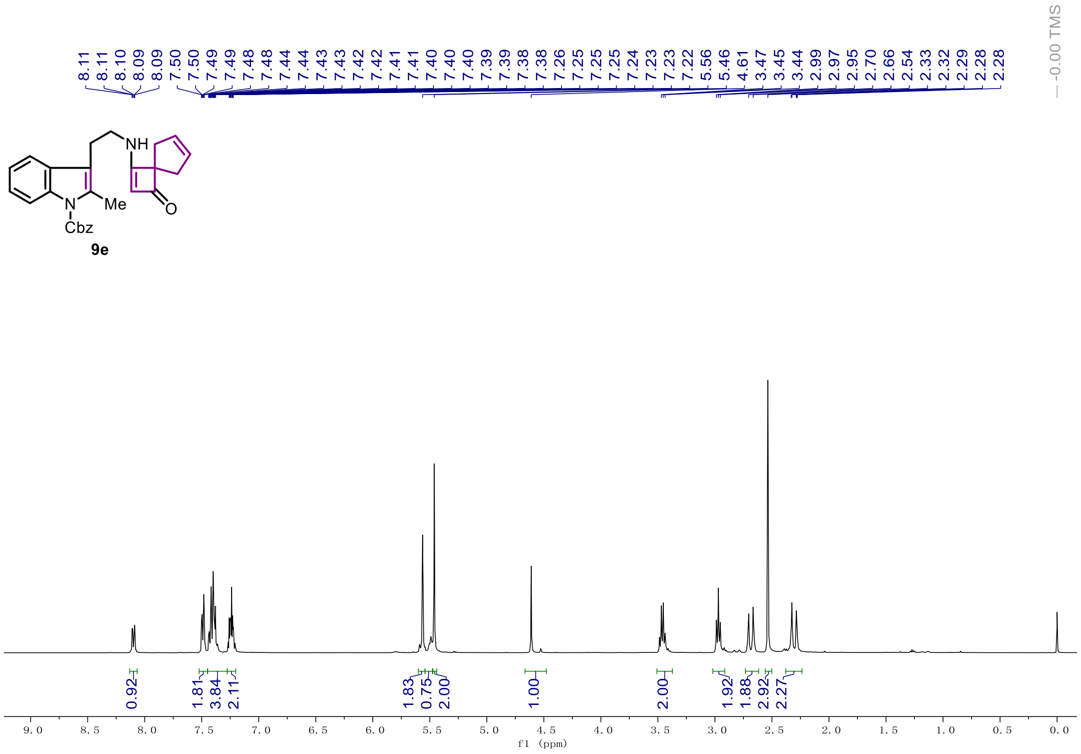

^{1}H NMR spectra of **9e** (400 MHz, Chloroform-*d*)

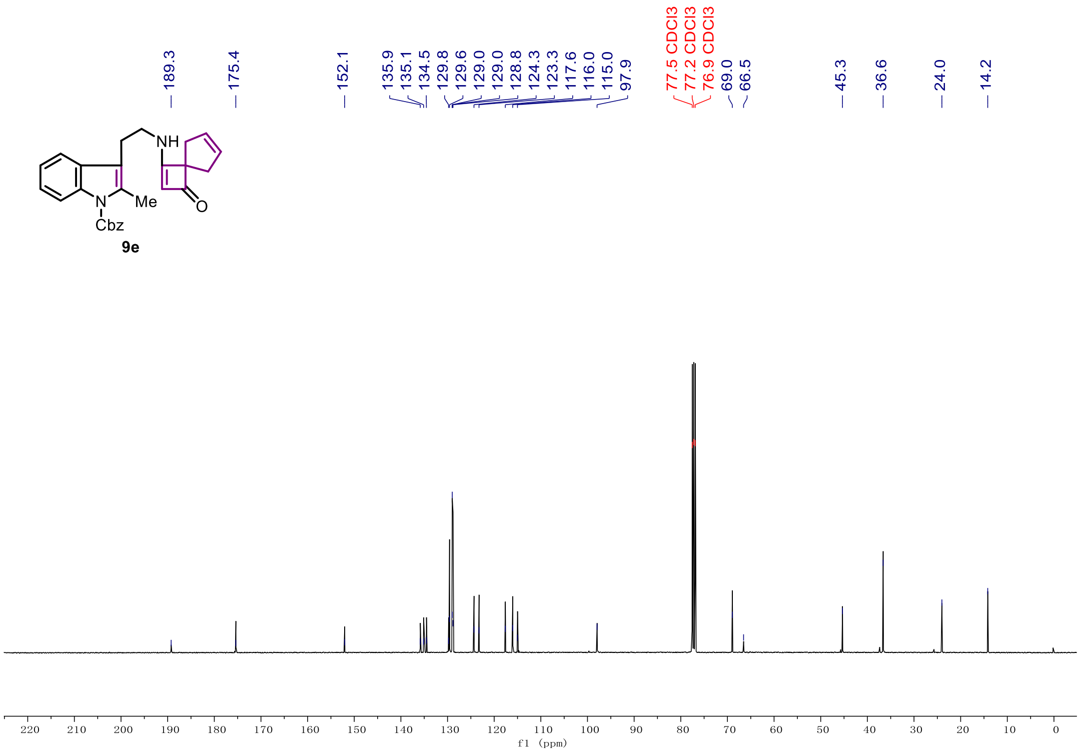

^{13}C NMR spectra of **9e** (101 MHz, Chloroform-*d*)

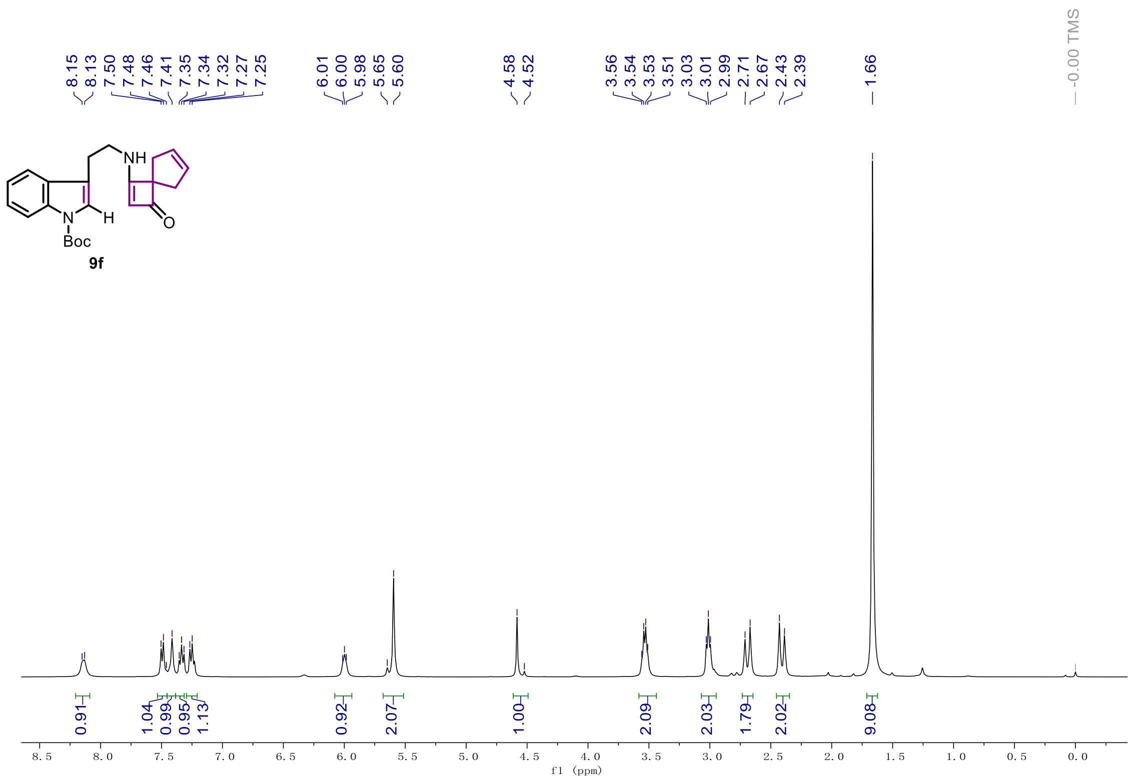

^{1}H NMR spectra of **9f** (400 MHz, Chloroform-d)

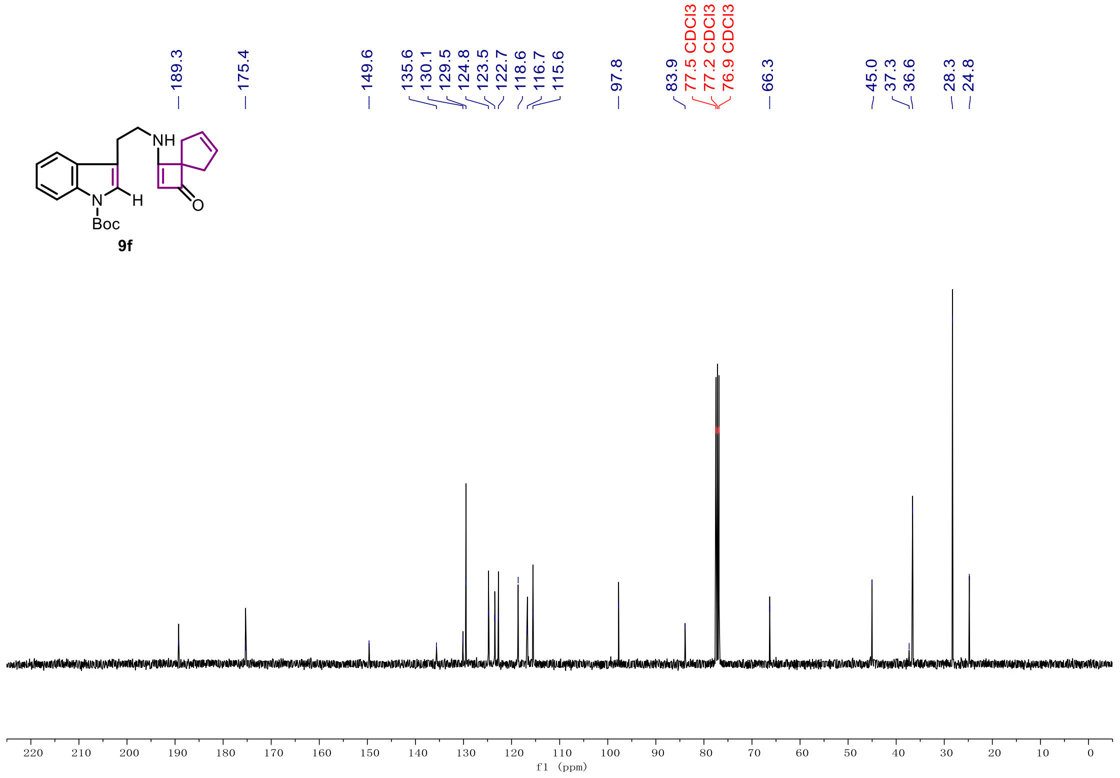

^{13}C NMR spectra of **9f** (101 MHz, Chloroform-d)

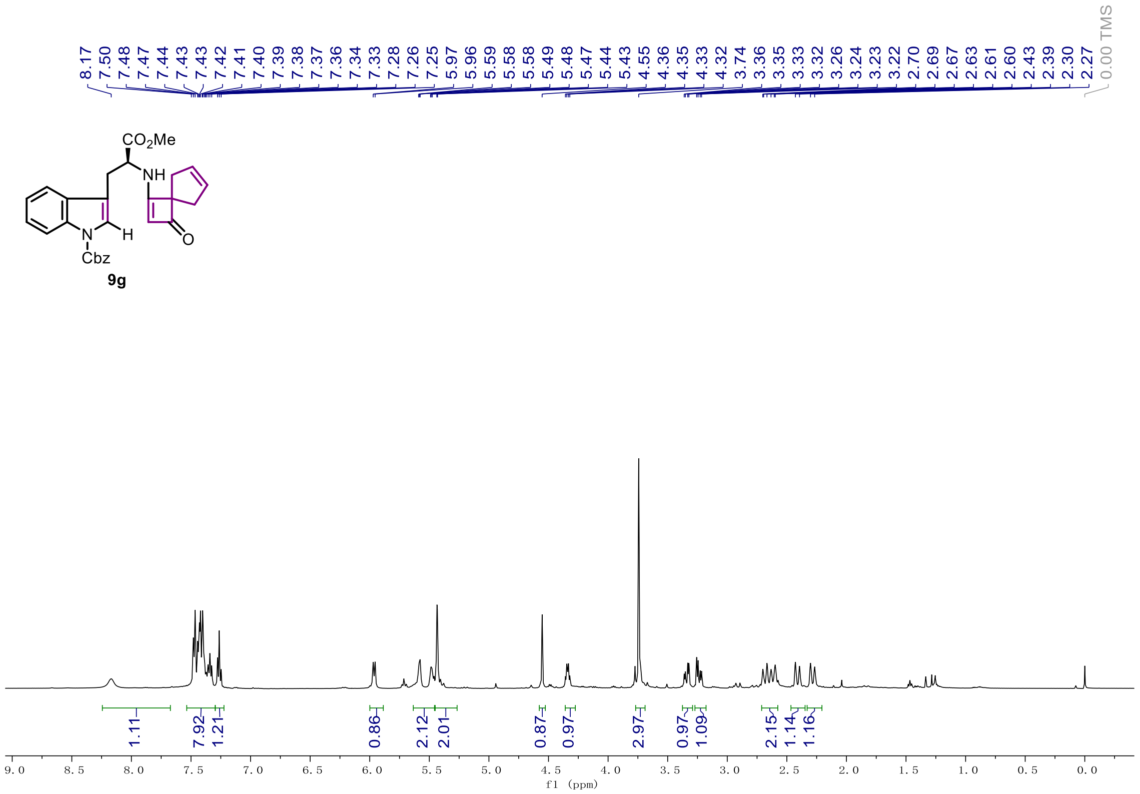

^{1}H NMR spectra of **9g** (500 MHz, Chloroform-*d*)

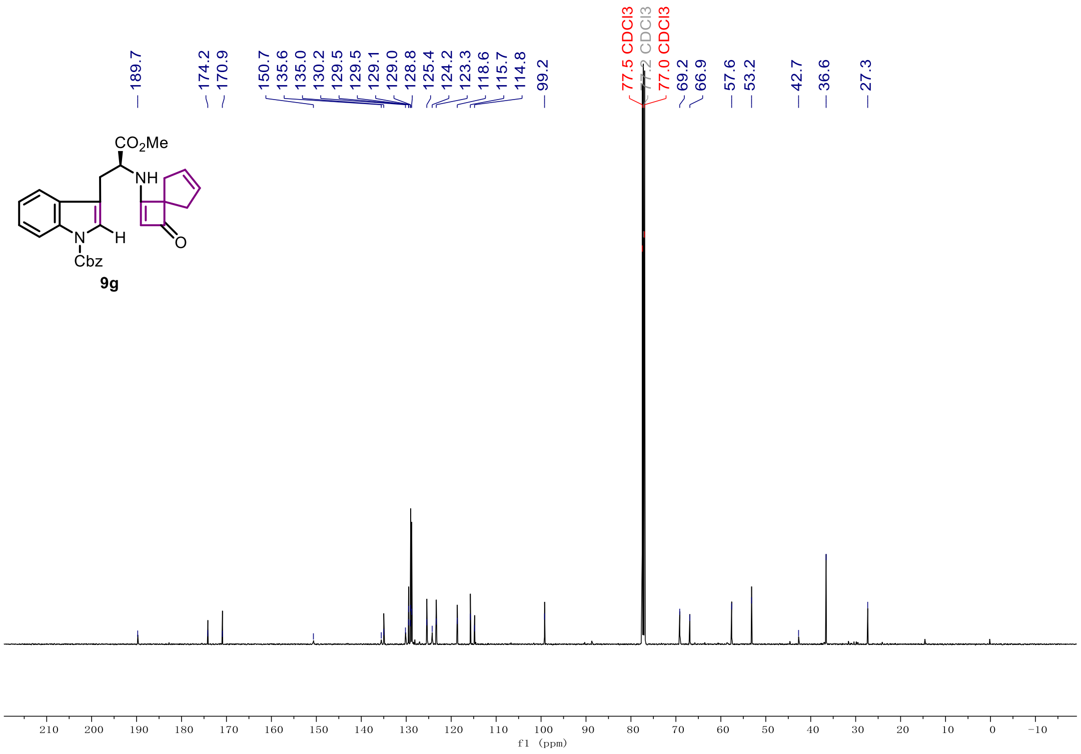

^{13}C NMR spectra of **9g** (126 MHz, Chloroform-*d*)

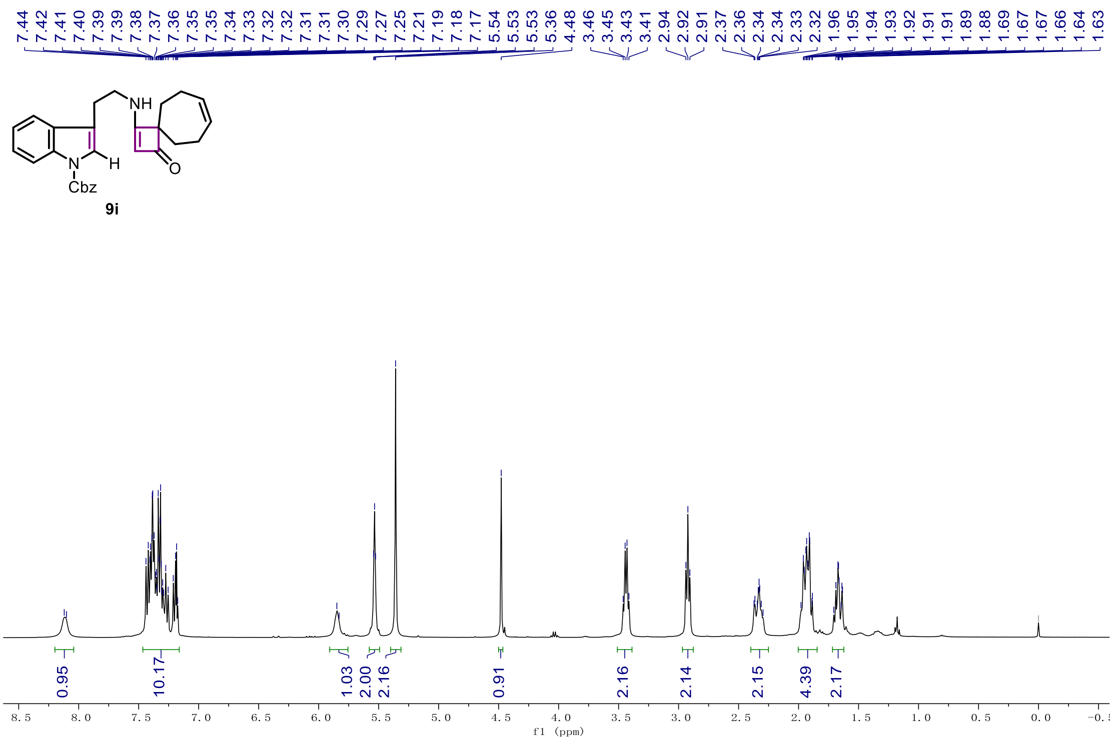

^{1}H NMR spectra of **9i** (400 MHz, Chloroform-*d*)

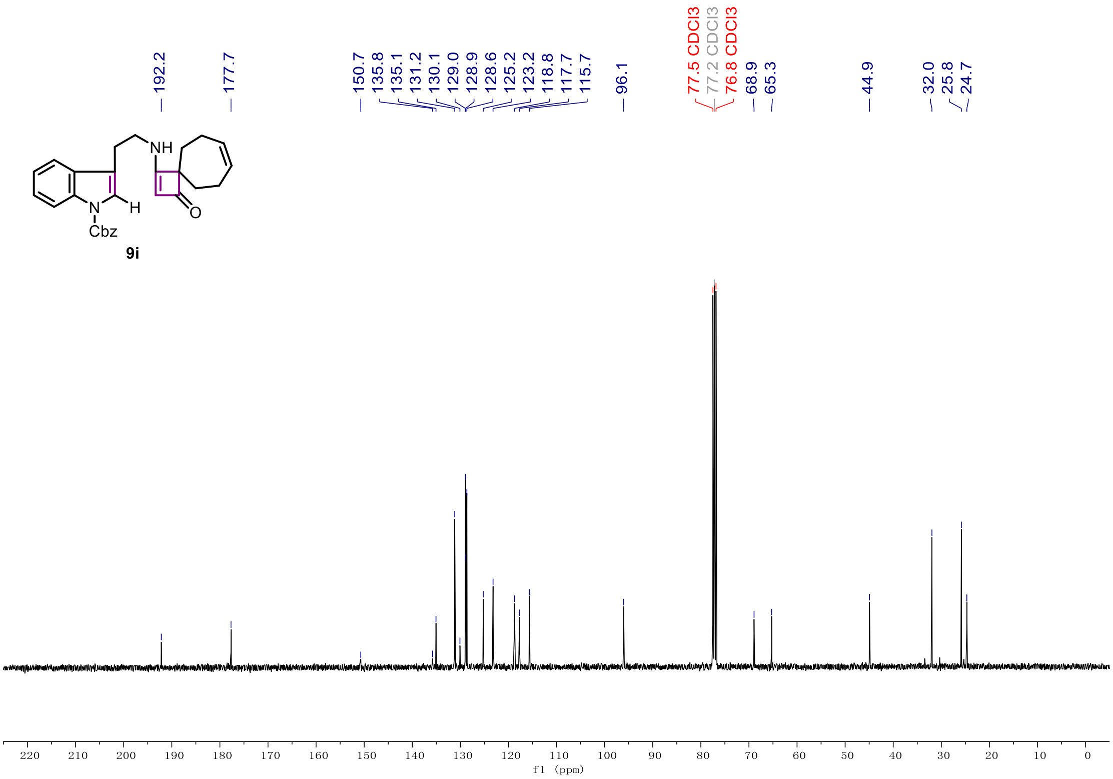

^{13}C NMR spectra of **9i** (101 MHz, Acetonitrile-*d*)

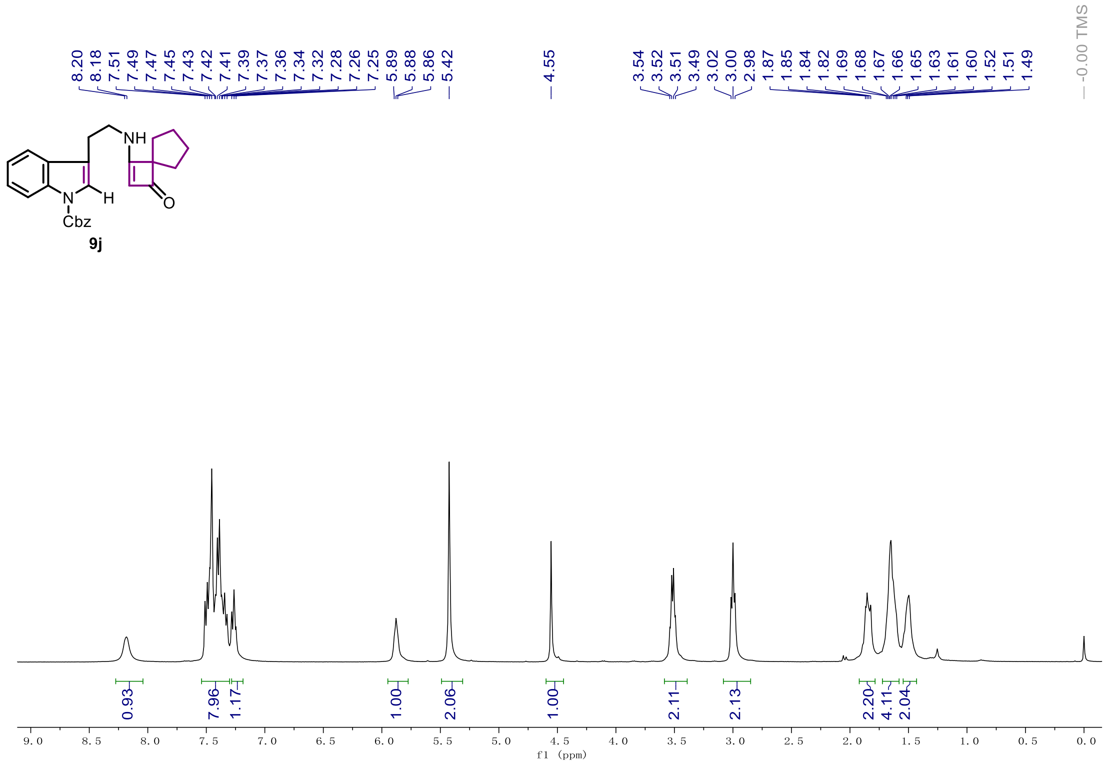

^{1}H NMR spectra of **9j** (400 MHz, Chloroform-*d*)

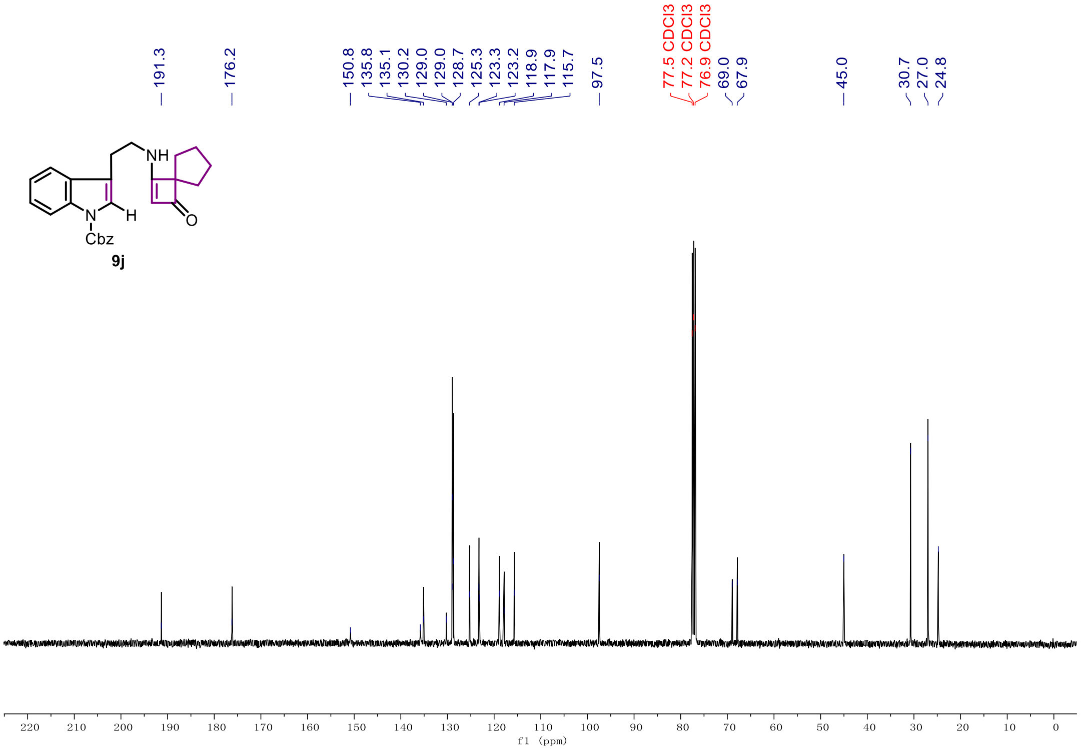

^{13}C NMR spectra of **9j** (101 MHz, Chloroform-*d*)

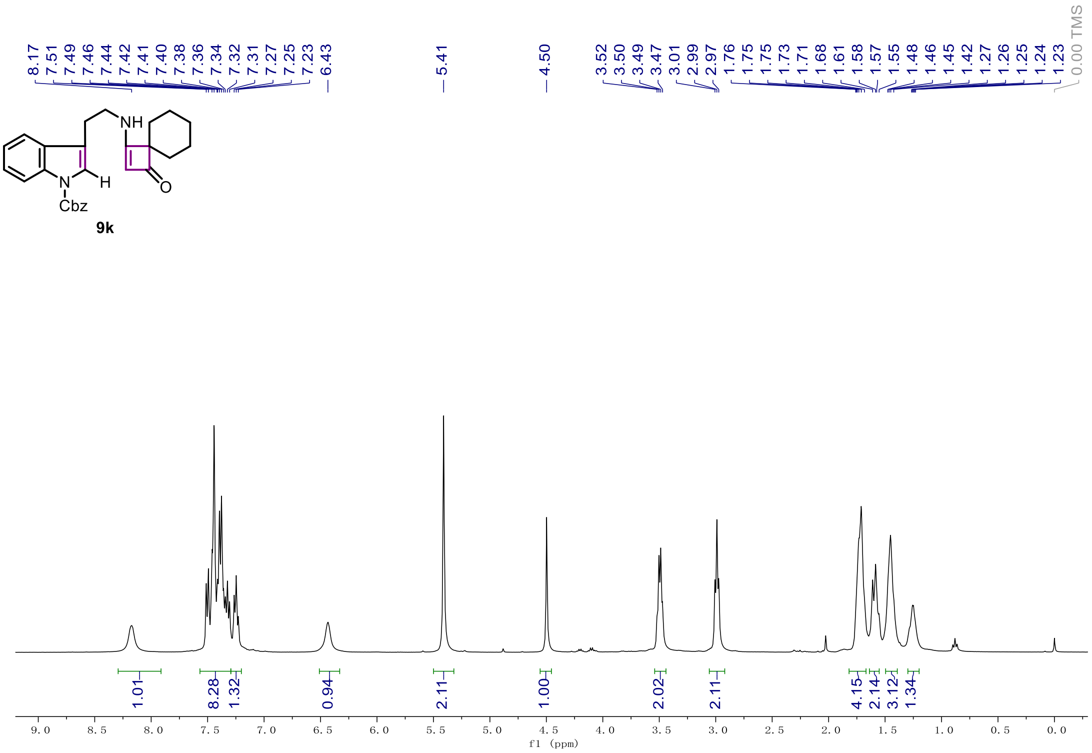

^{1}H NMR spectra of **9k** (400 MHz, Chloroform-*d*)

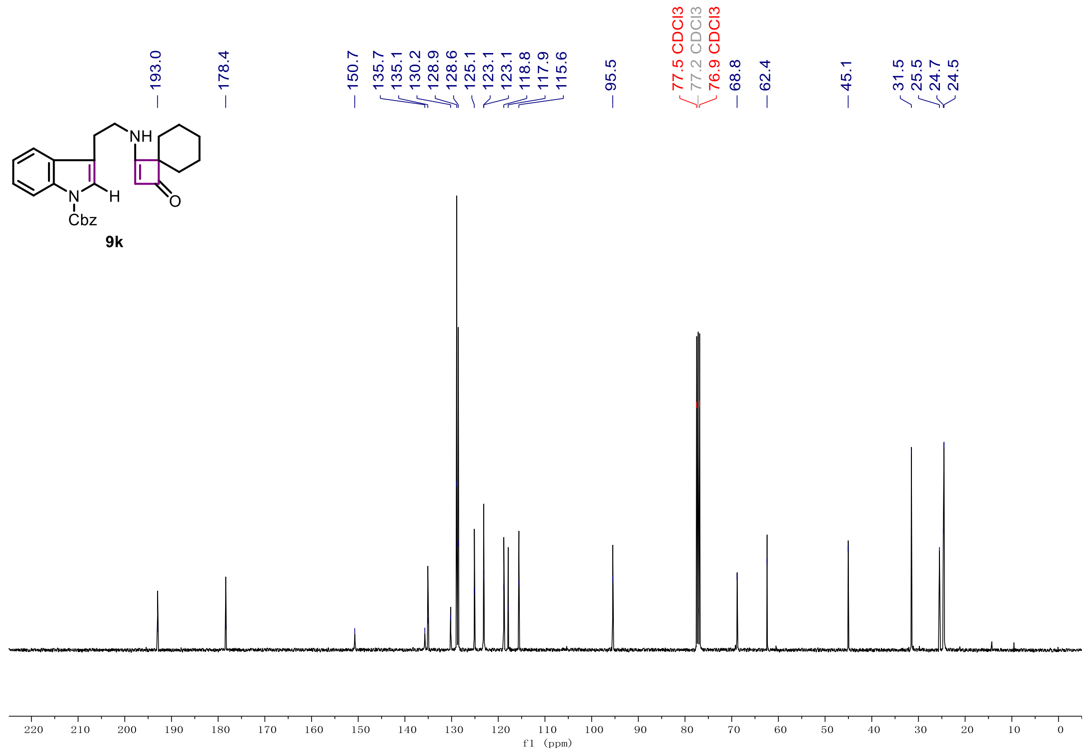

^{13}C NMR spectra of **9k** (101 MHz, Chloroform-*d*)

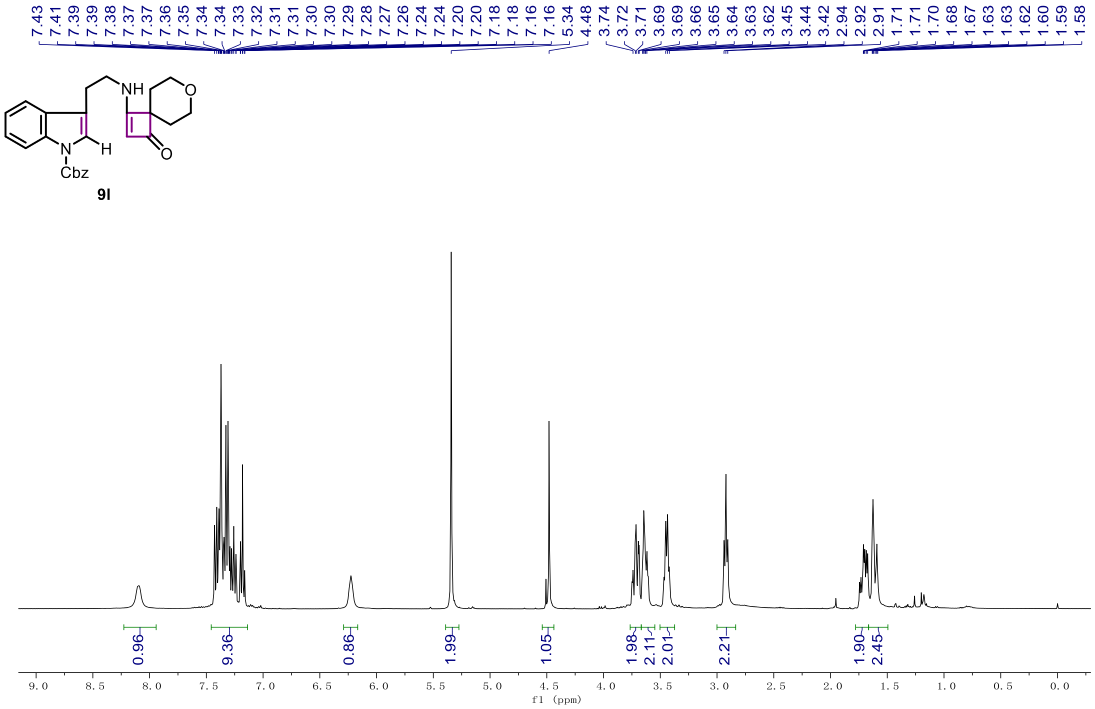

^{1}H NMR spectra of **9l** (400 MHz, Chloroform-*d*)

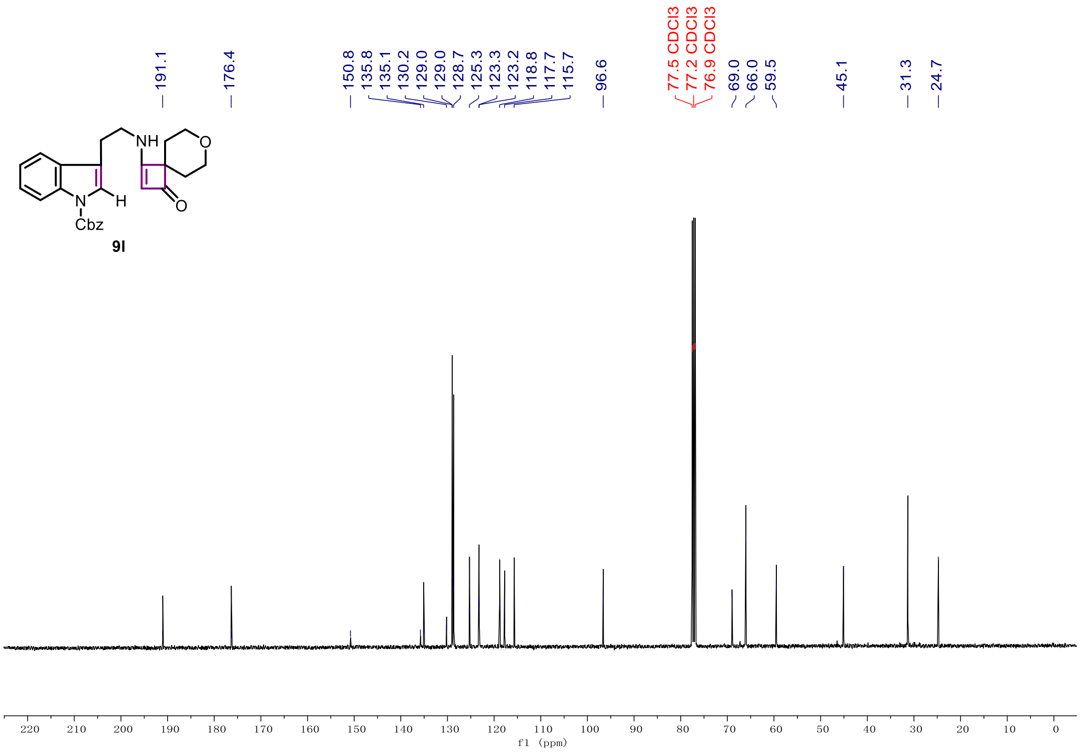

^{13}C NMR spectra of **9l** (101 MHz, Chloroform-*d*)

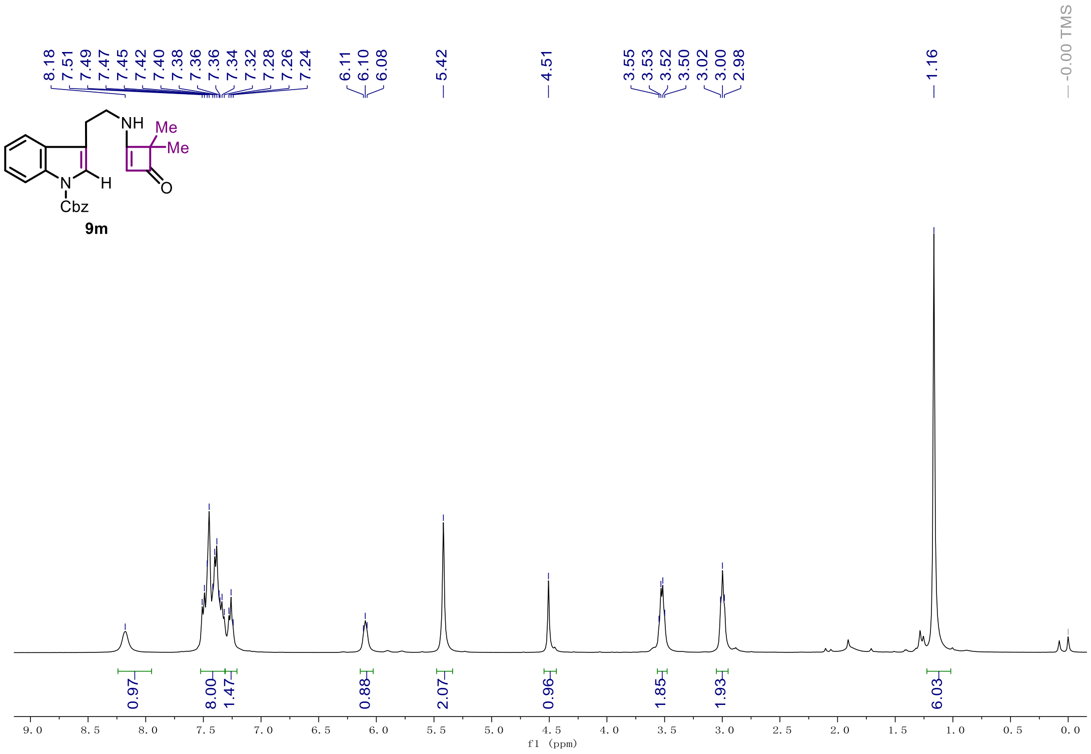

^{1}H NMR spectra of **9m** (400 MHz, Chloroform-*d*)

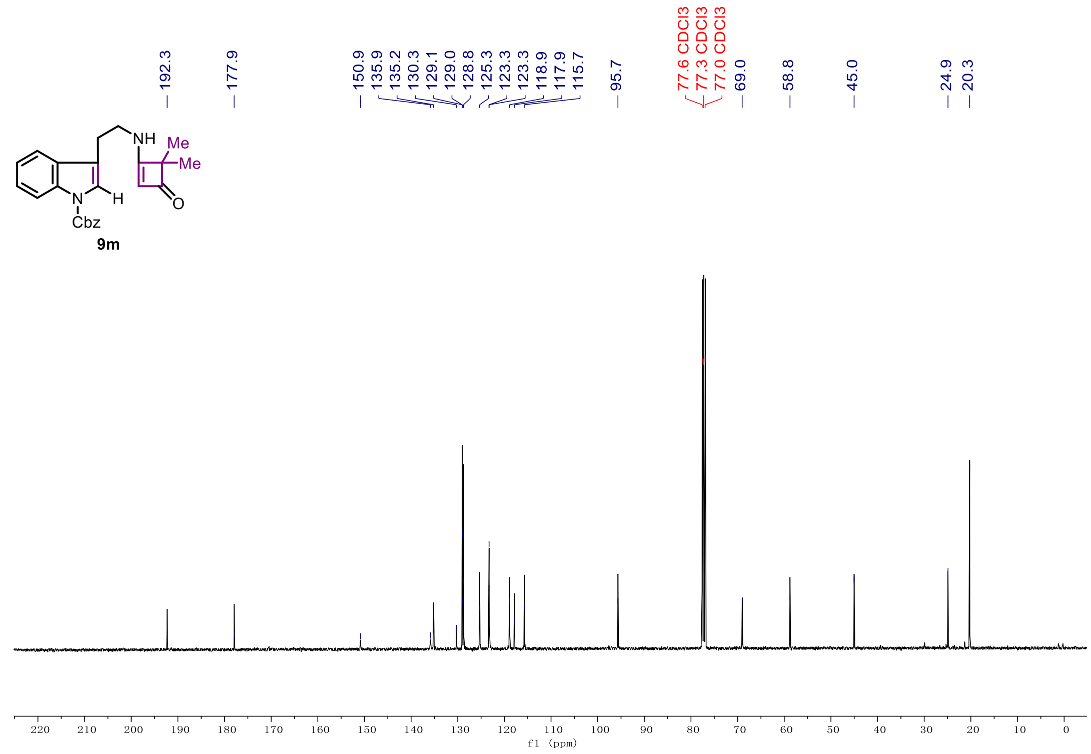

^{13}C NMR spectra of **9m** (101 MHz, Chloroform-*d*)

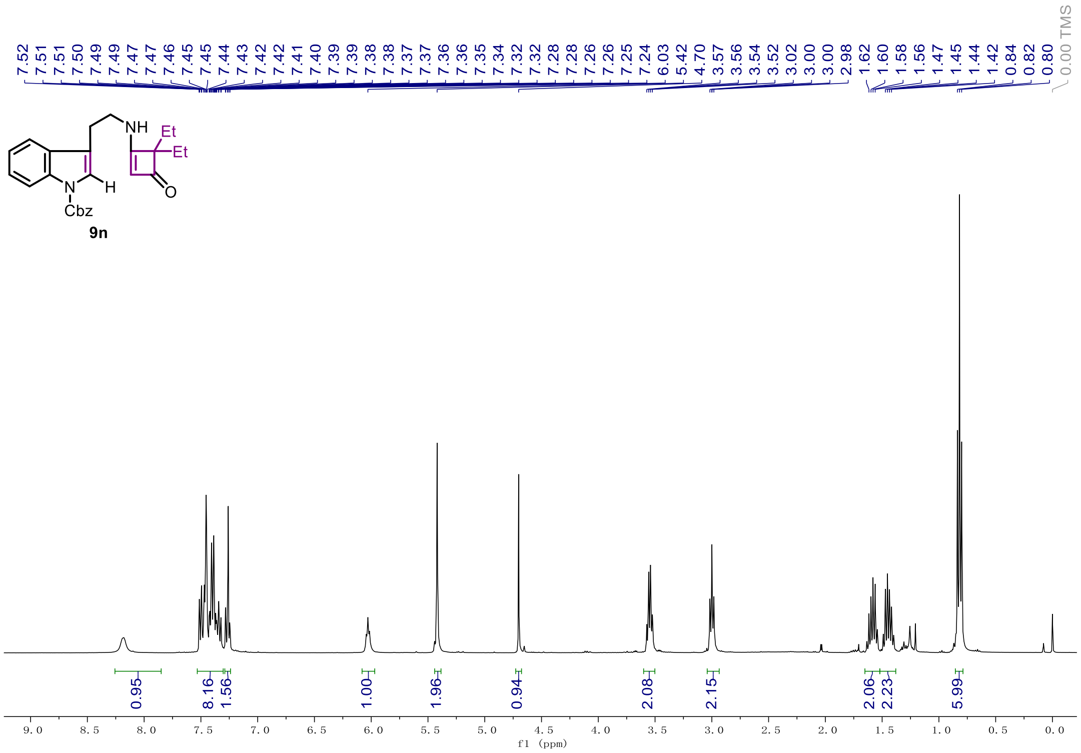

^{1}H NMR spectra of **9n** (400 MHz, Chloroform-*d*)

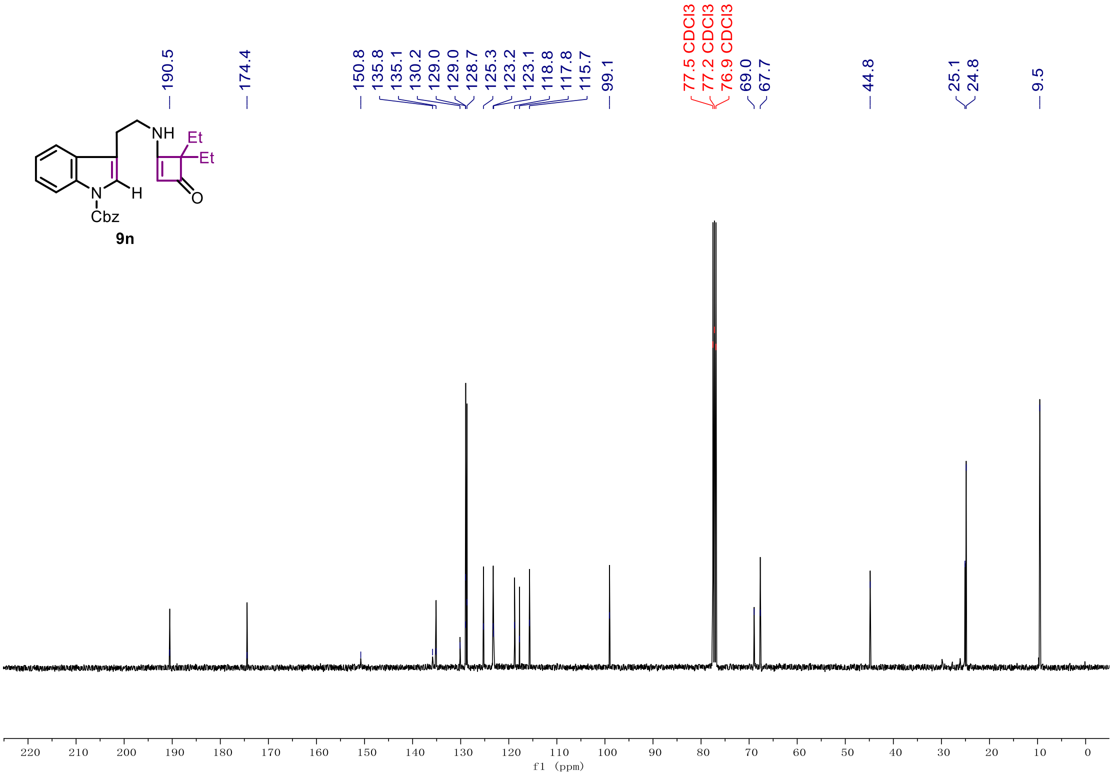

^{13}C NMR spectra of **9n** (101 MHz, Chloroform-*d*)

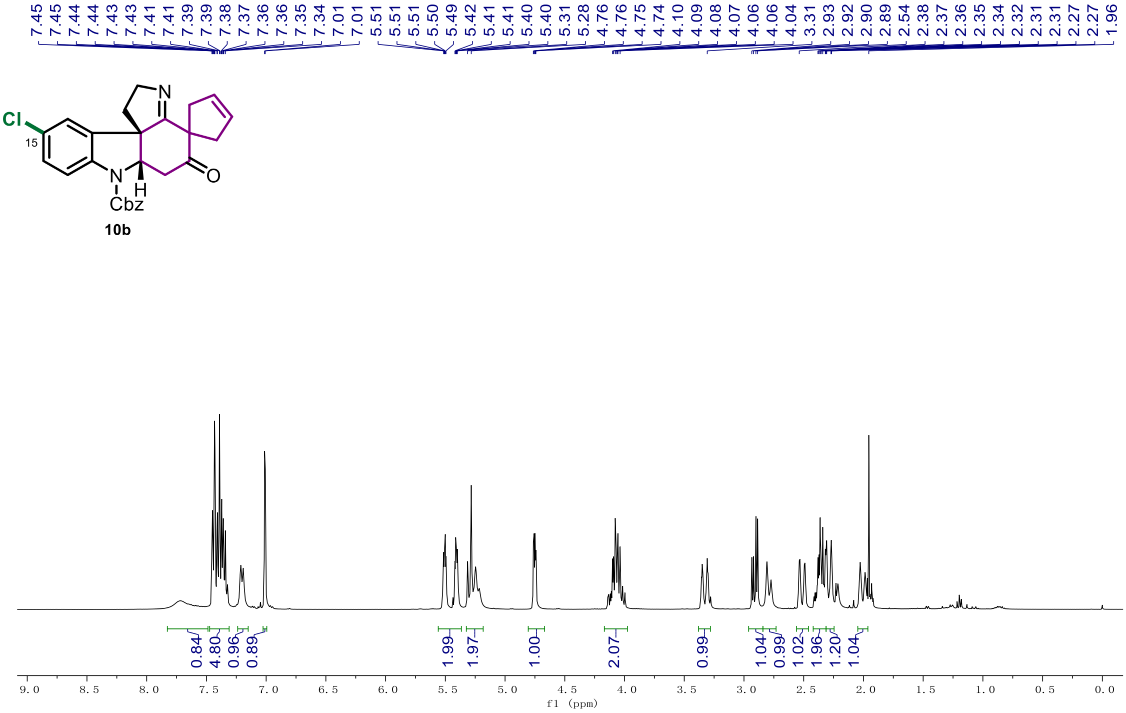

^{1}H NMR spectra of **10b** (400 MHz, Acetonitrile-d_3)

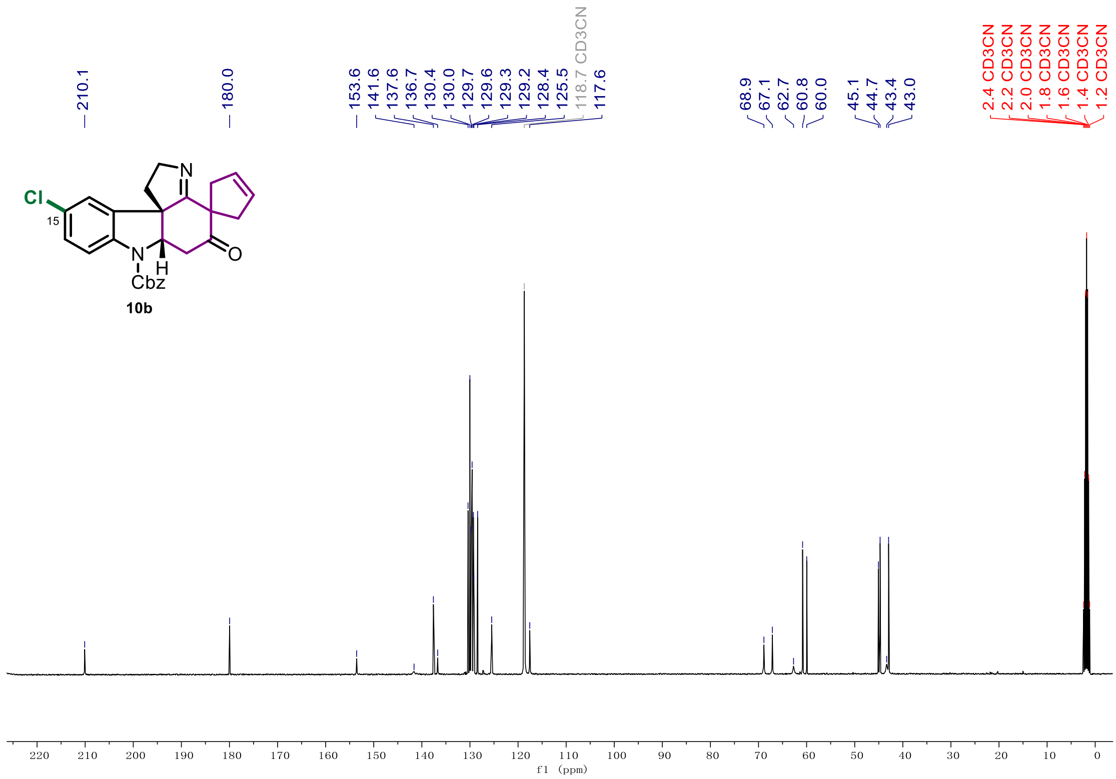

^{13}C NMR spectra of **10b** (101 MHz, Acetonitrile-d_3)

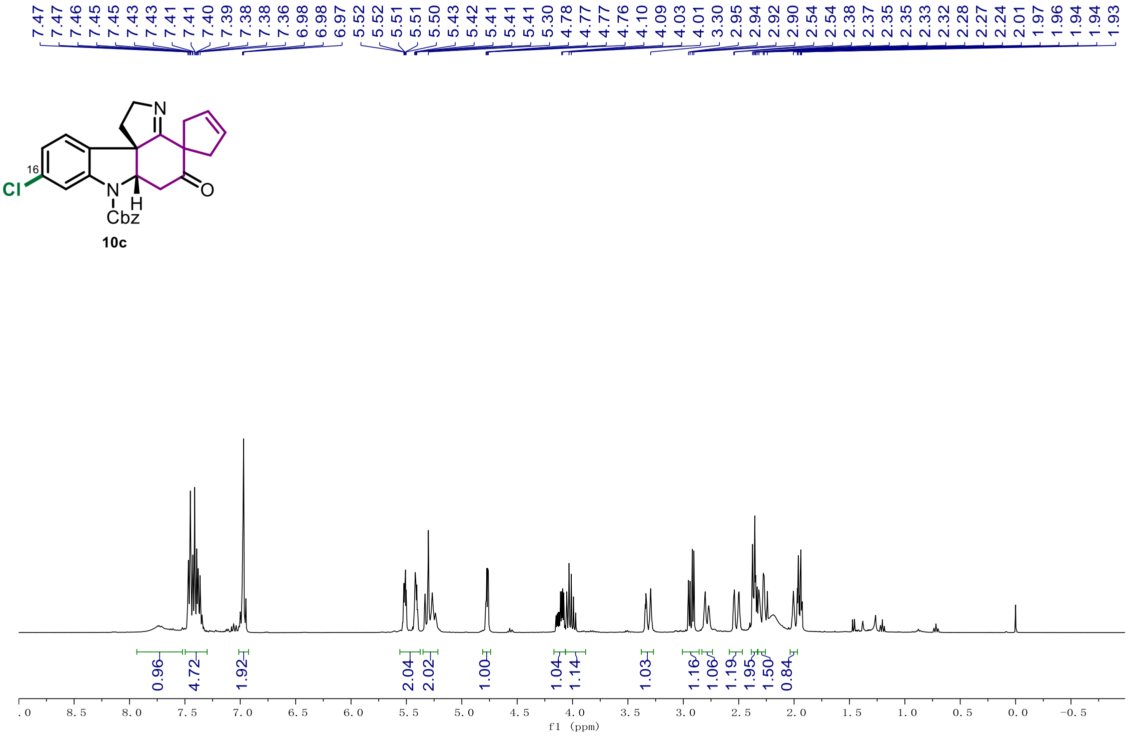

^{1}H NMR spectra of **10c** (400 MHz, Acetonitrile-d_3)

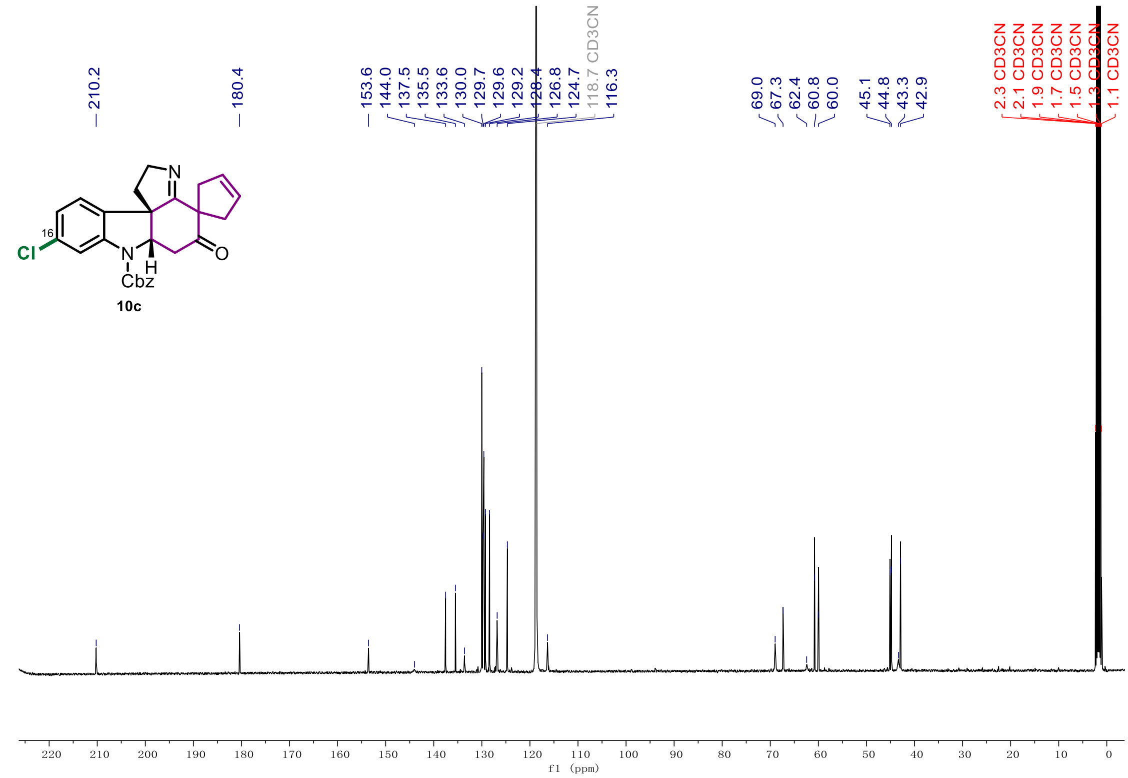

^{13}C NMR spectra of **10c** (101 MHz, Acetonitrile-d_3)

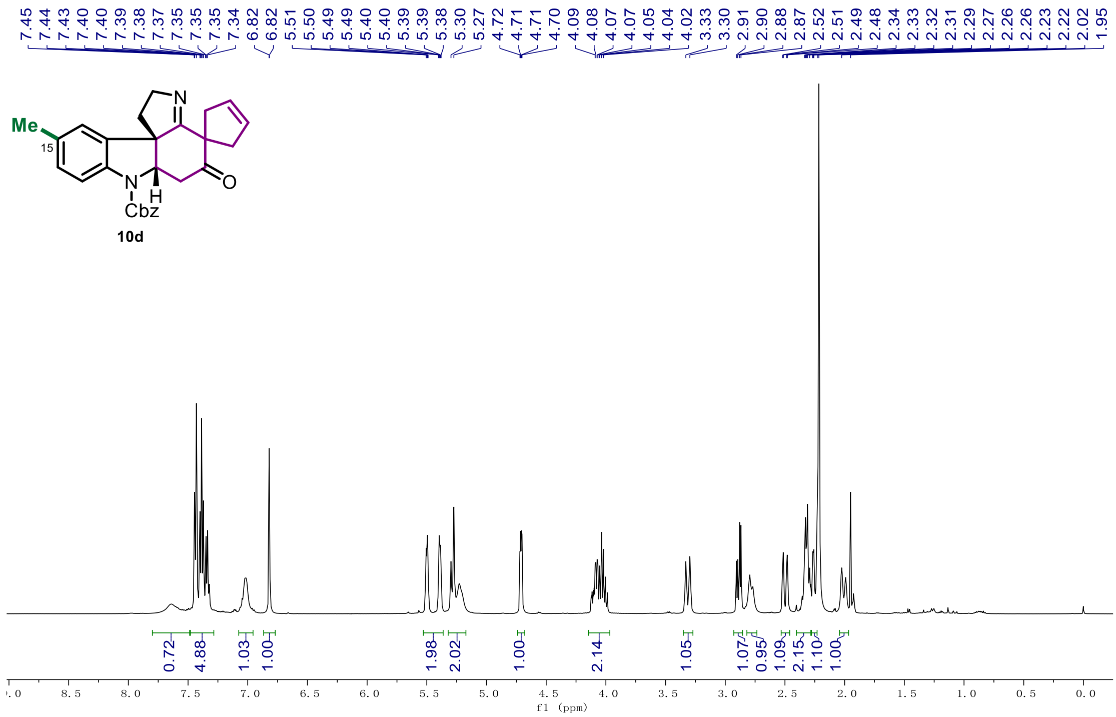

^{1}H NMR spectra of **10d** (400 MHz, Acetonitrile-d_3)

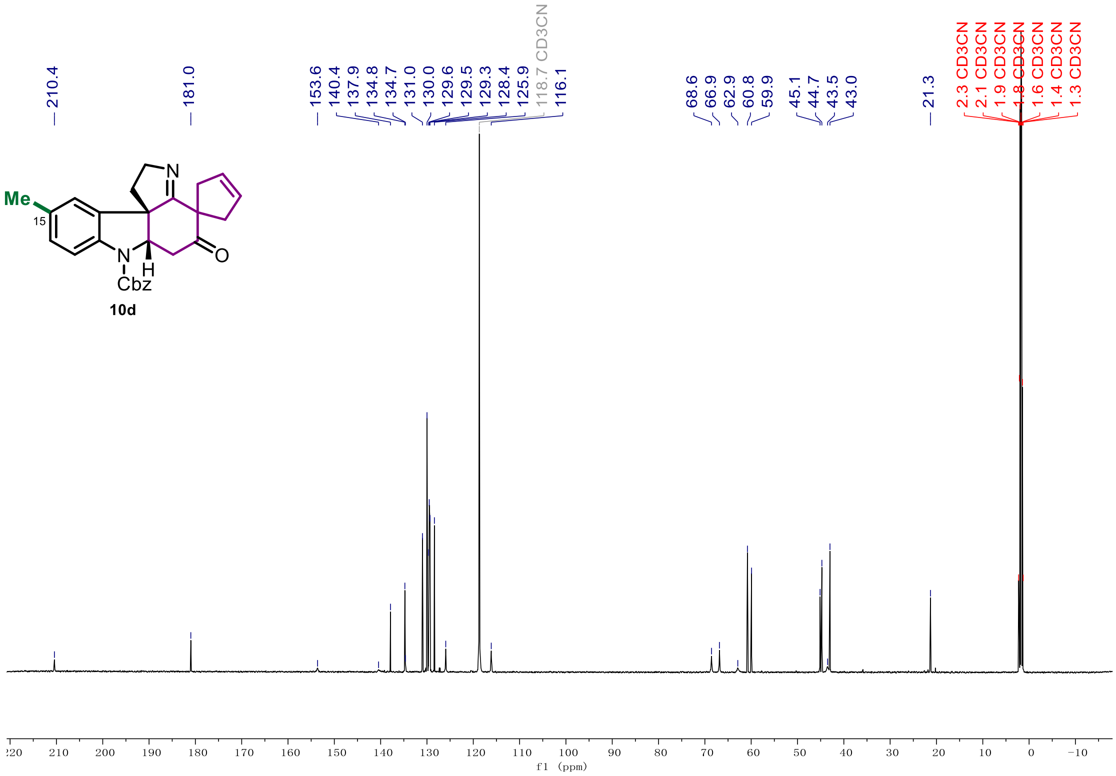

^{13}C NMR spectra of **10d** (101 MHz, Acetonitrile-d_3)

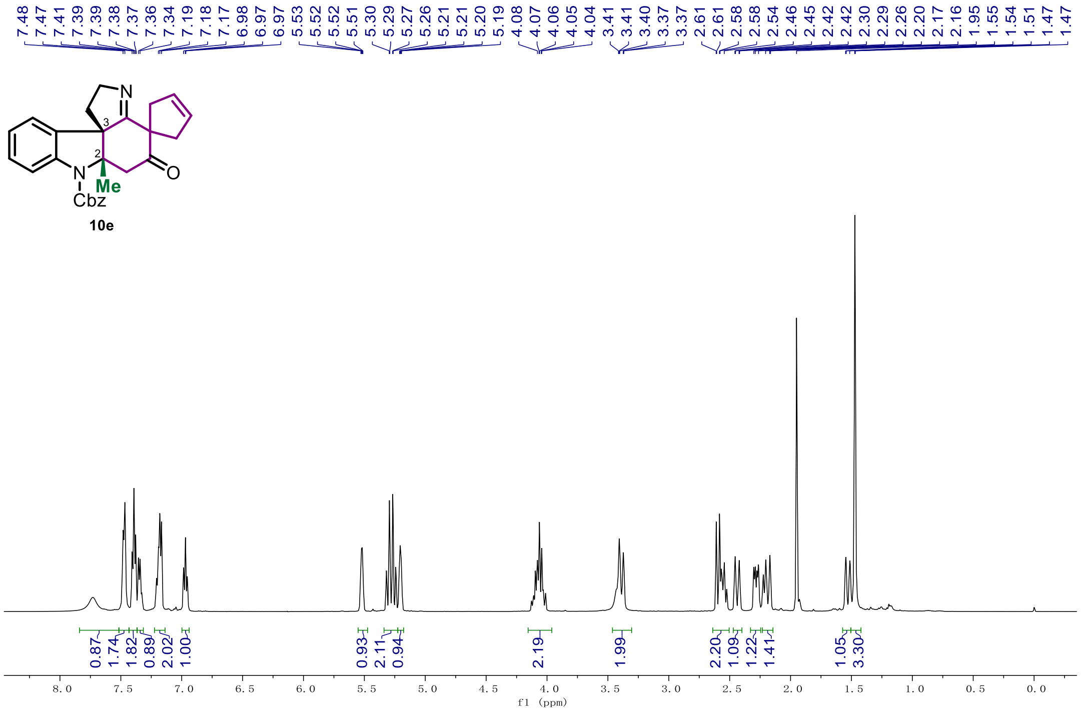

^{1}H NMR spectra of **10e** (500 MHz, Acetonitrile-d_3)

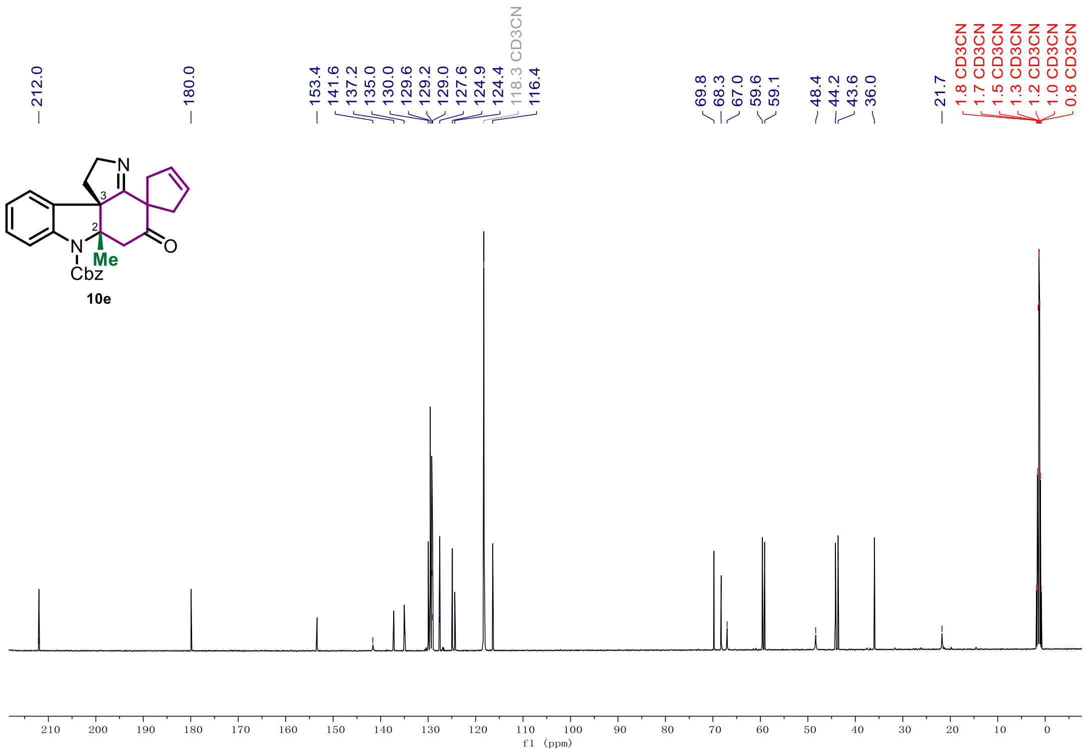

^{13}C NMR spectra of **10e** (126 MHz, Acetonitrile-d_3)

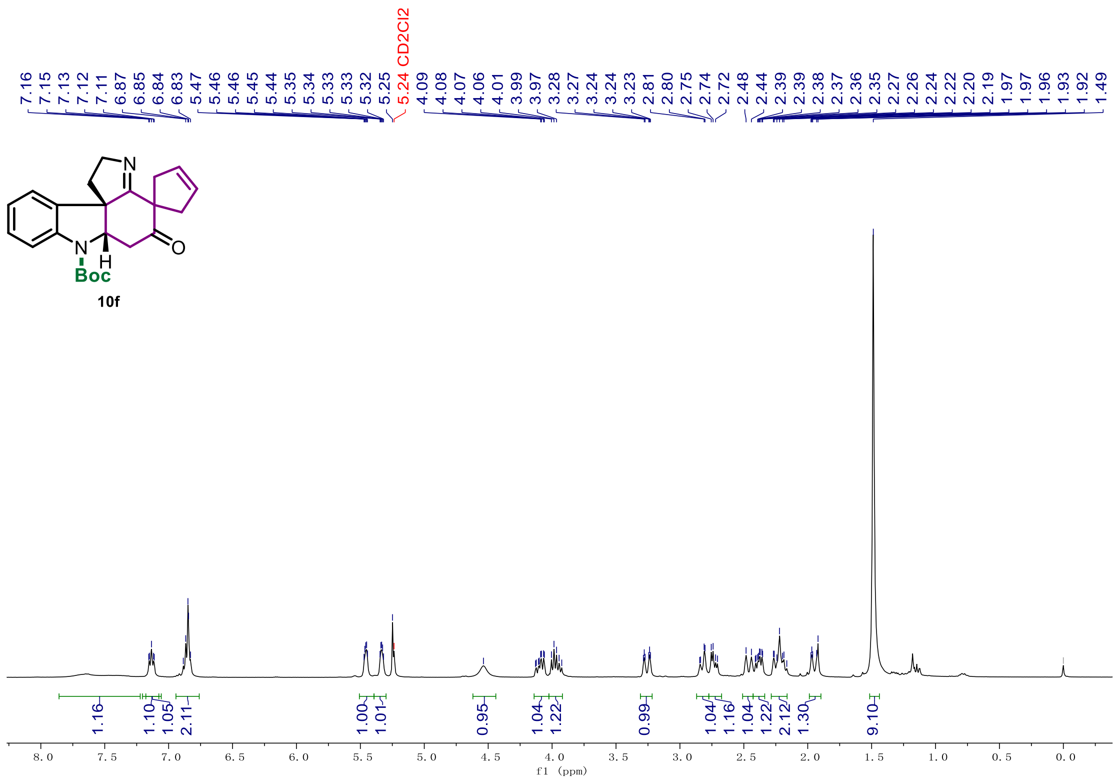

^{1}H NMR spectra of **10f** (500 MHz, Acetonitrile-d_3)

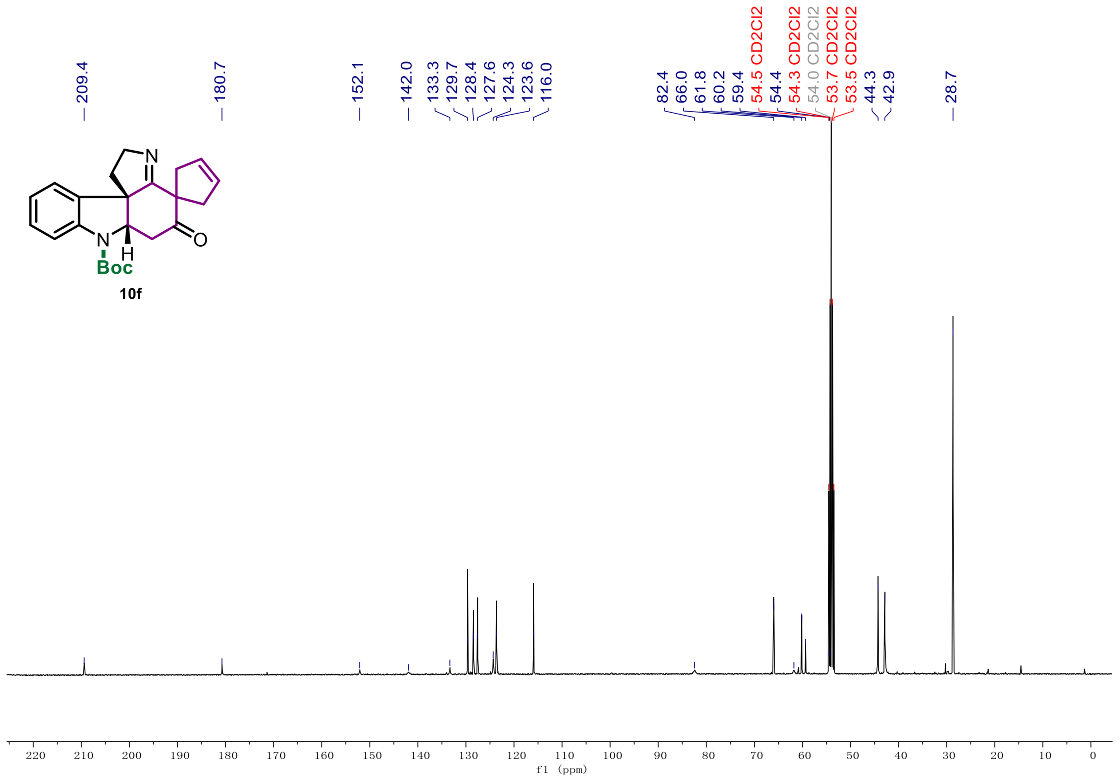

^{13}C NMR spectra of **10f** (126 MHz, Acetonitrile-d_3)

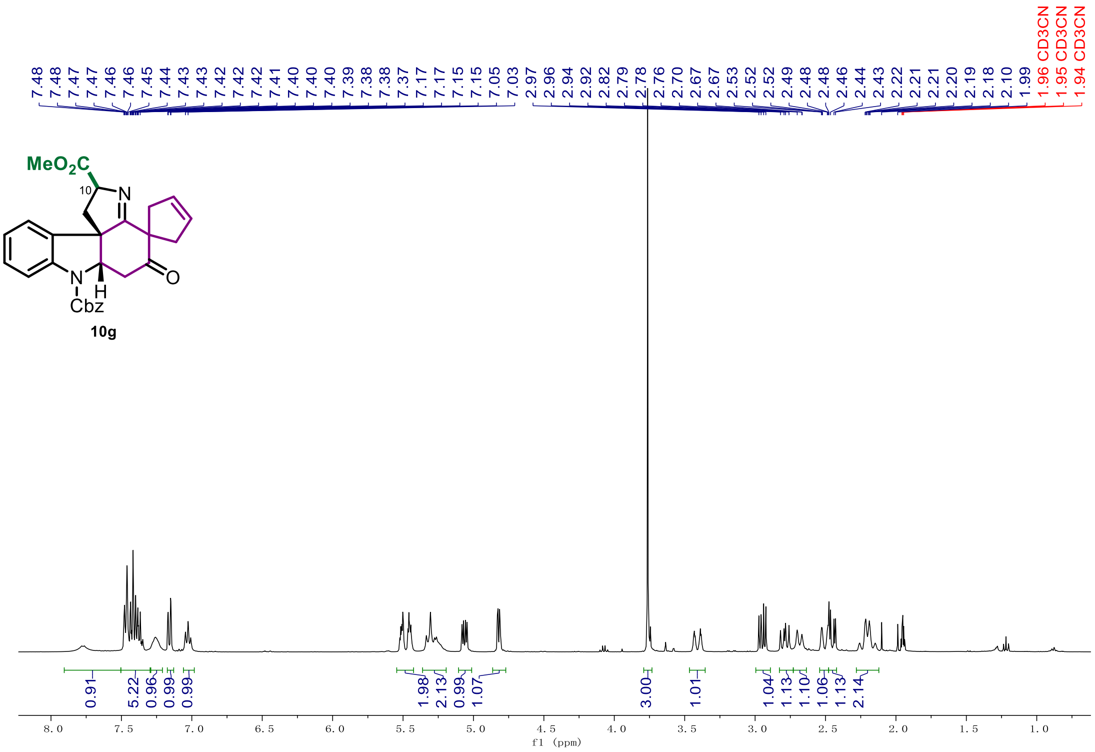

^{1}H NMR spectra of **10g** (400 MHz, Acetonitrile-d_3)

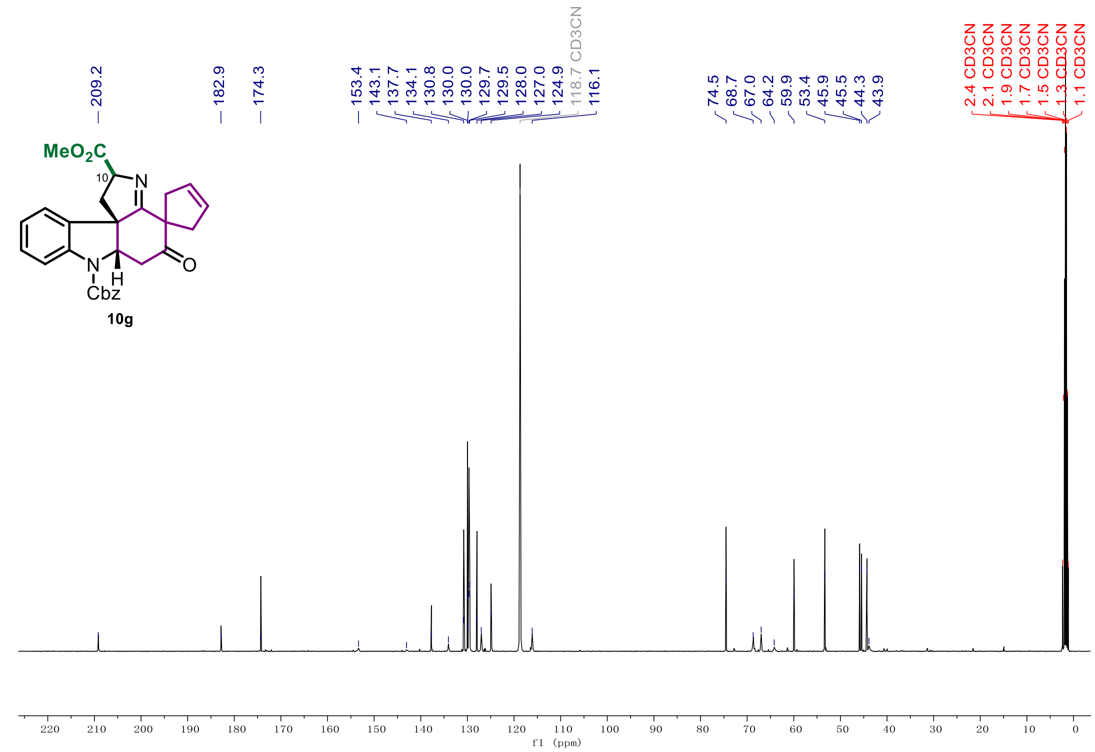

^{13}C NMR spectra of **10g** (101 MHz, Acetonitrile-d_3)

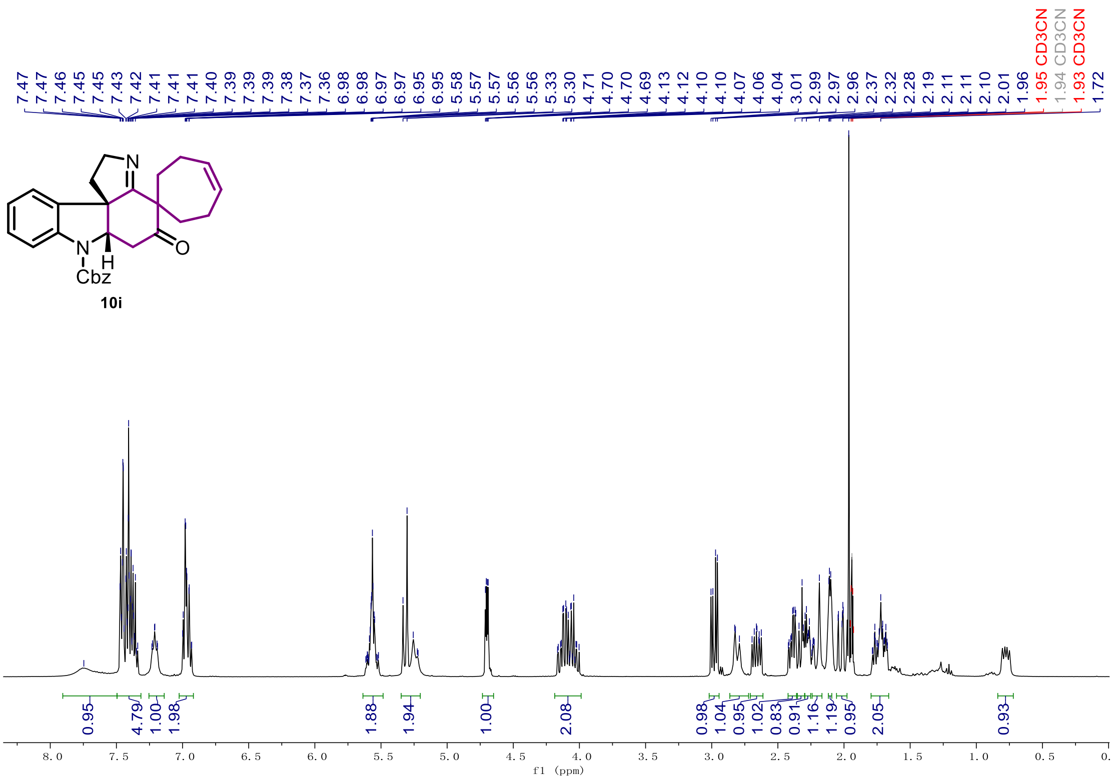

^{1}H NMR spectra of **10i** (400 MHz, Acetonitrile -*d*)

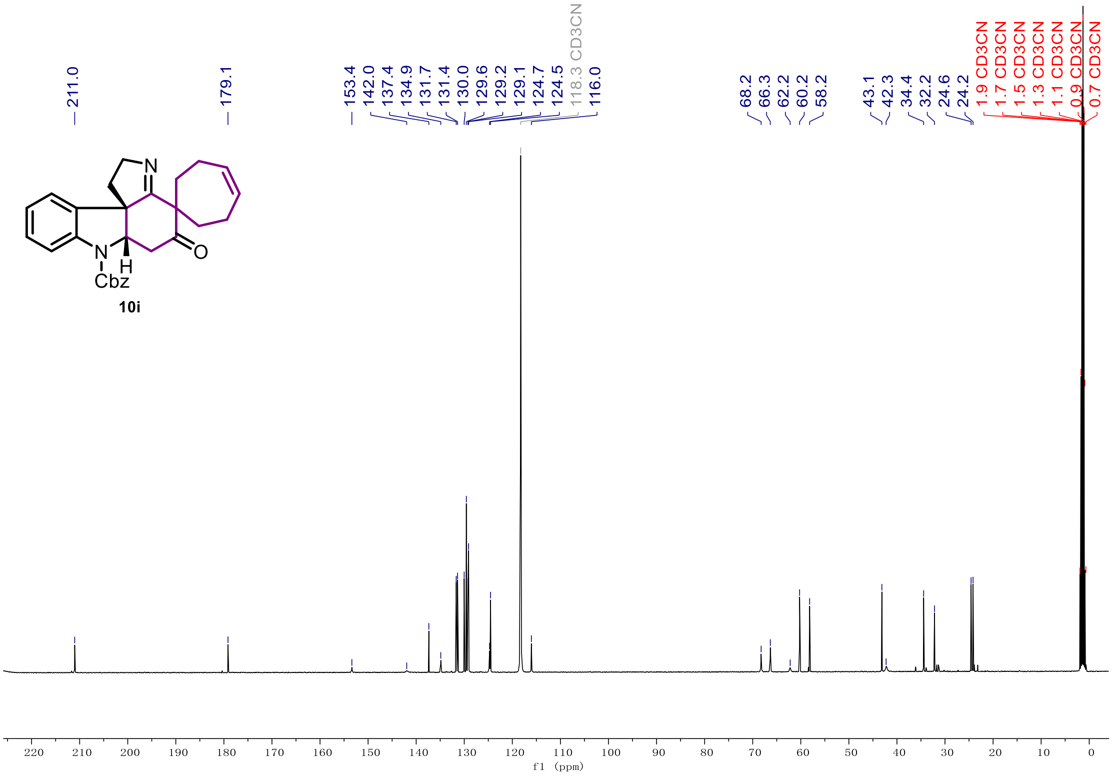

^{13}C NMR spectra of **10i** (101 MHz, Acetonitrile-*d*)

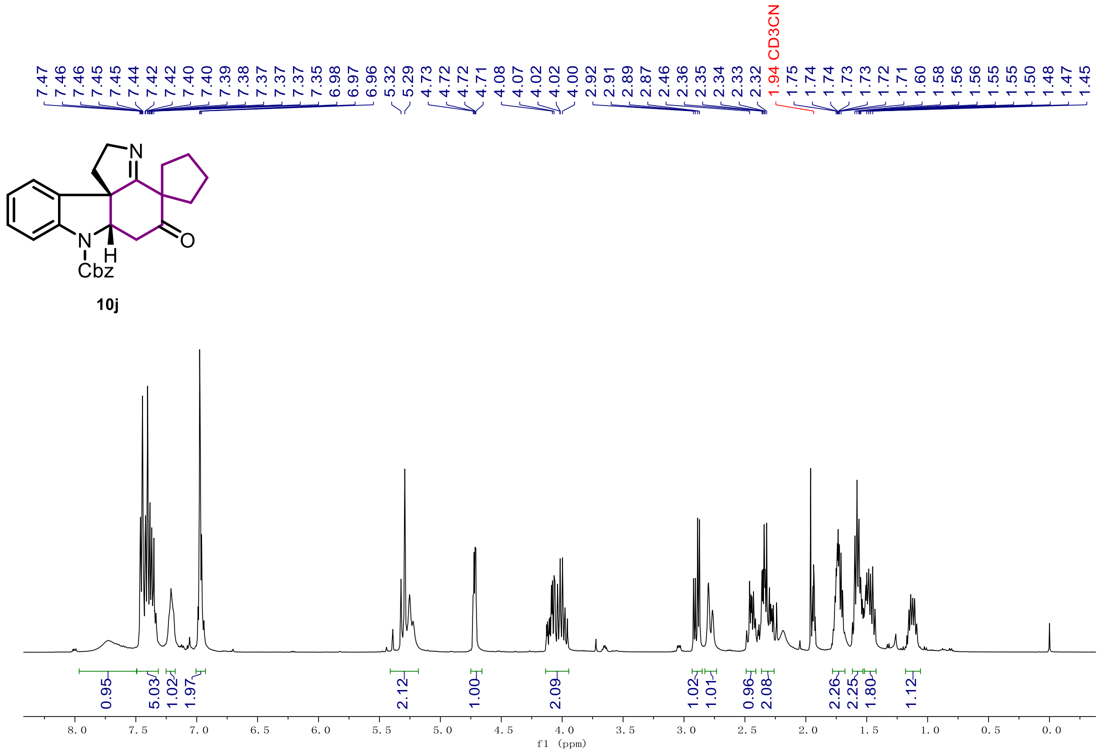

^{1}H NMR spectra of **10j** (400 MHz, Acetonitrile-d_3)

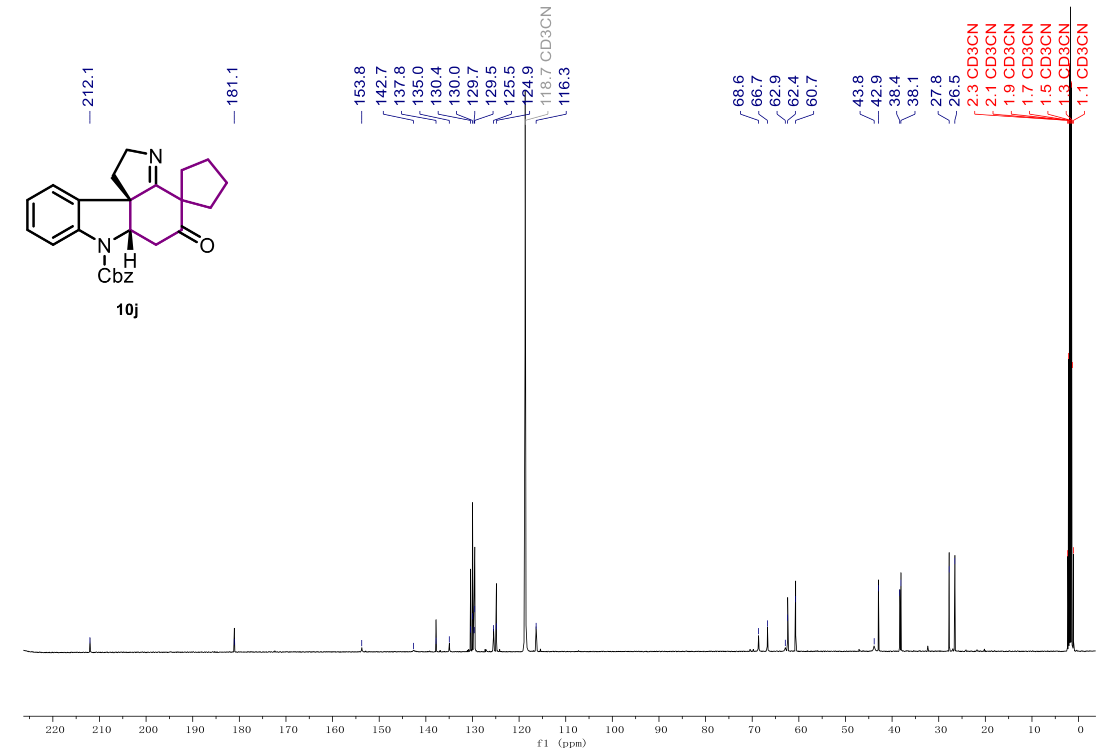

^{13}C NMR spectra of **10j** (101 MHz, Acetonitrile-d_3)

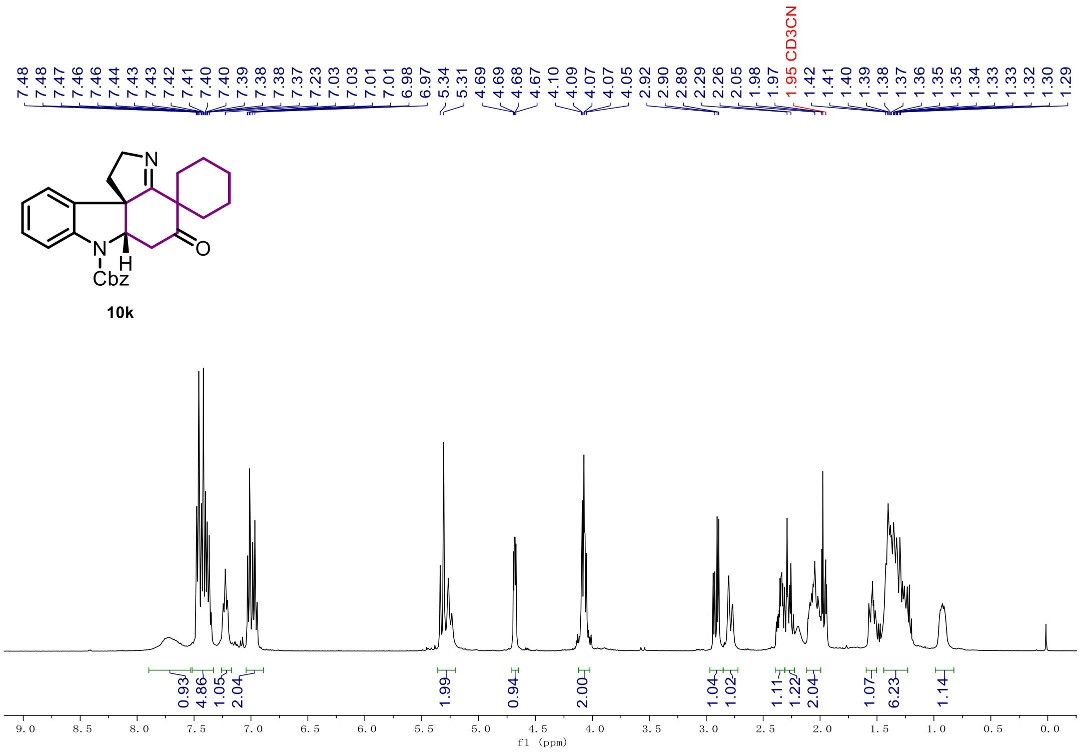

^{1}H NMR spectra of **10k** (400 MHz, Acetonitrile-d_3)

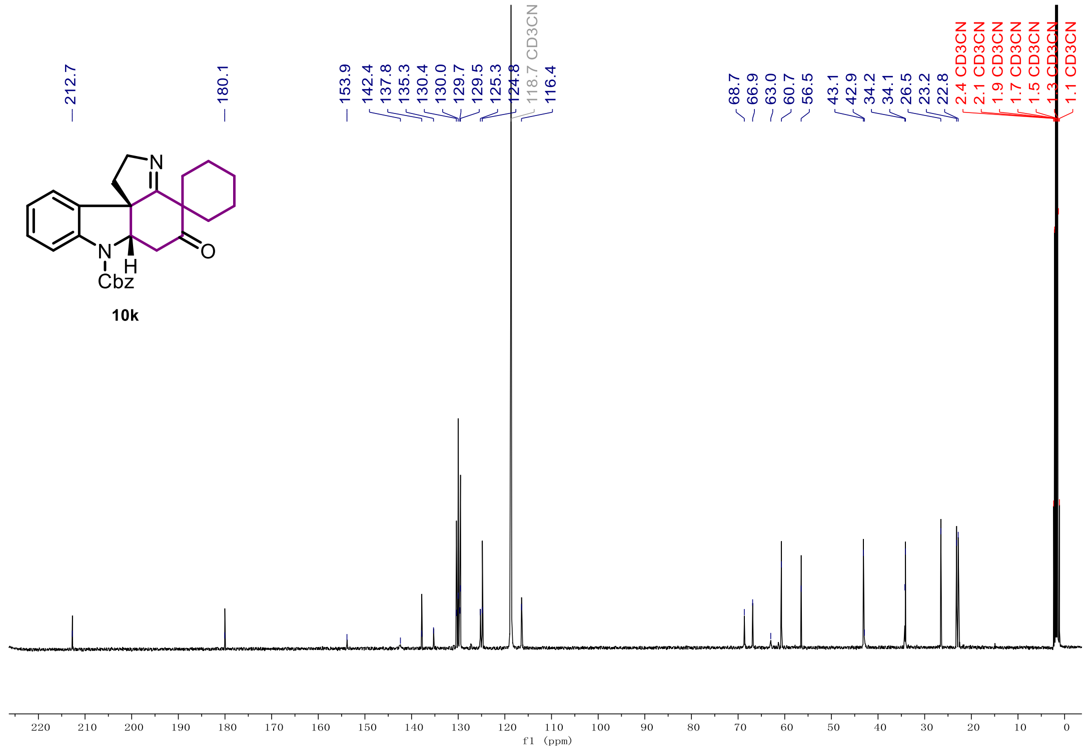

^{13}C NMR spectra of **10k** (101 MHz, Acetonitrile-d_3)

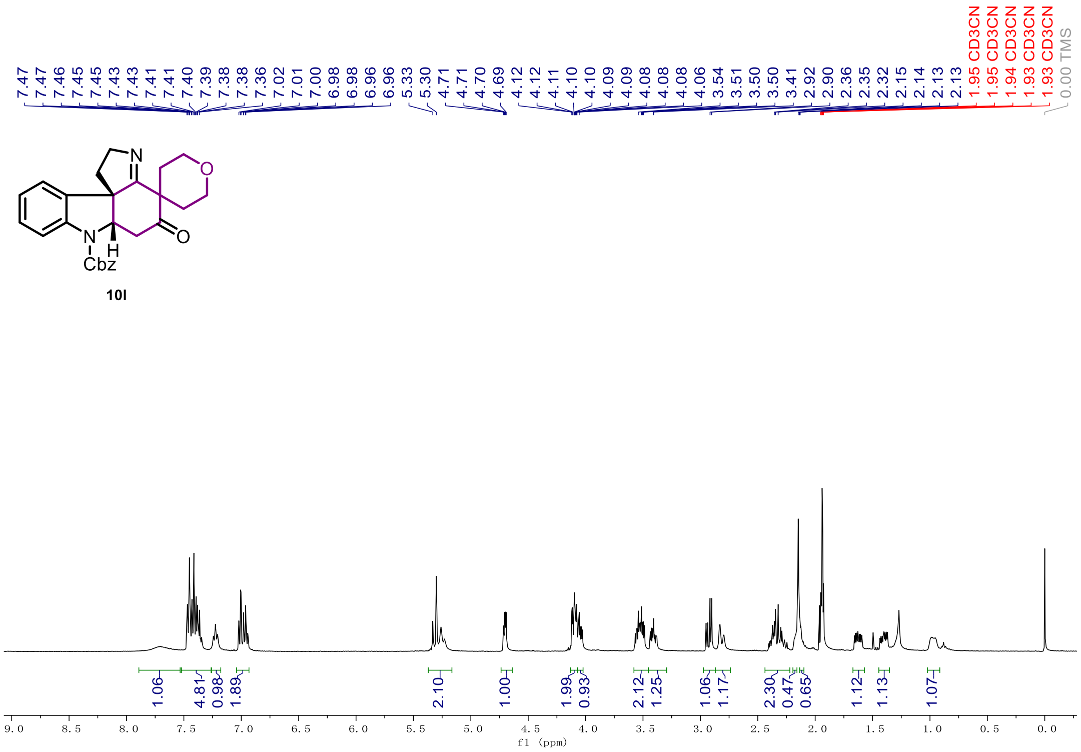

^{1}H NMR spectra of **10l** (400 MHz, Acetonitrile-d_3)

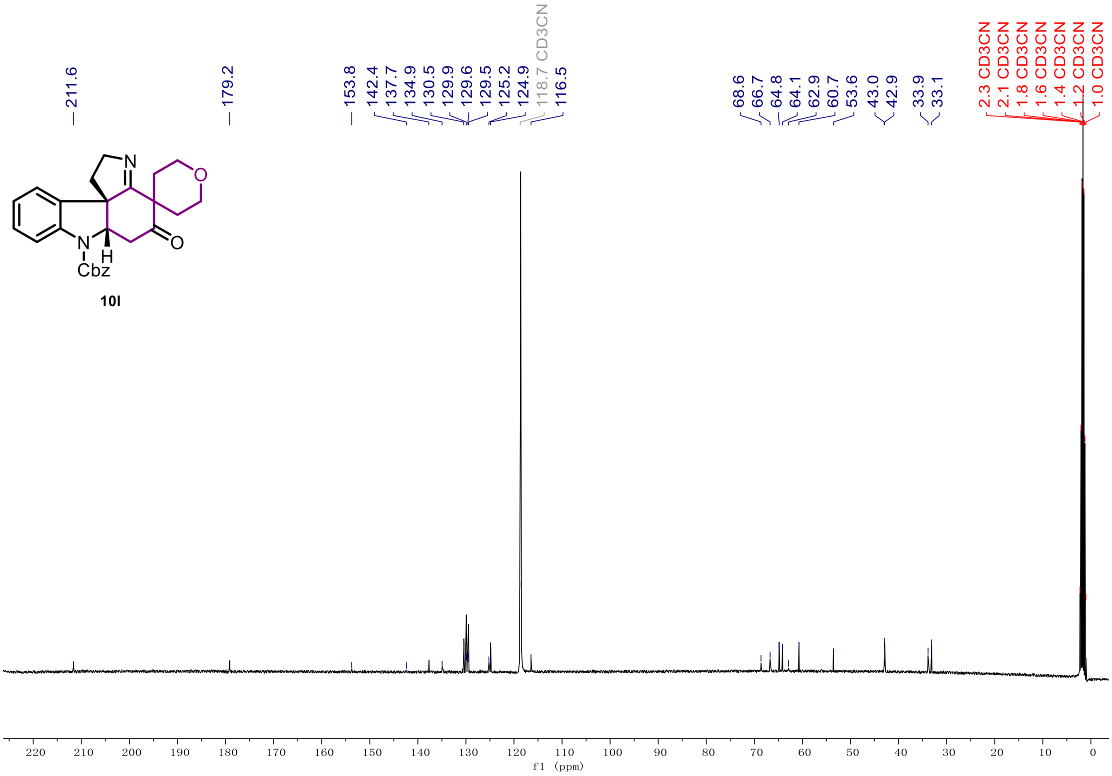

^{13}C NMR spectra of **10l** (101 MHz, Acetonitrile-d_3)

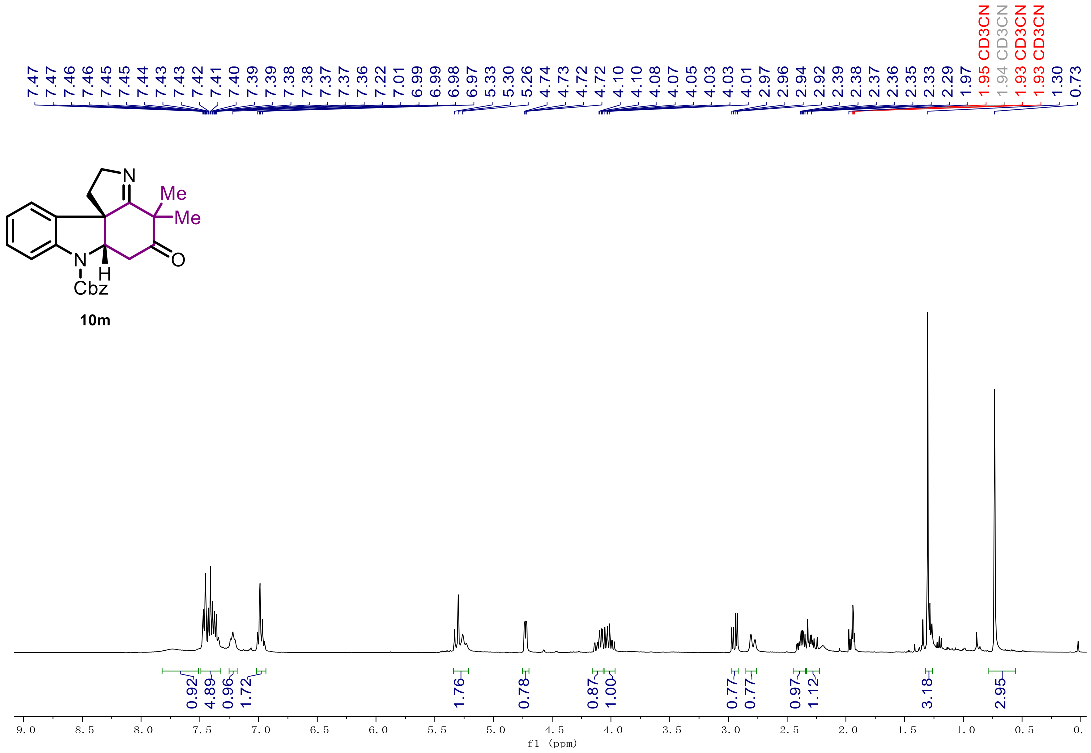

^{1}H NMR spectra of **10m** (400 MHz, Acetonitrile-d_3)

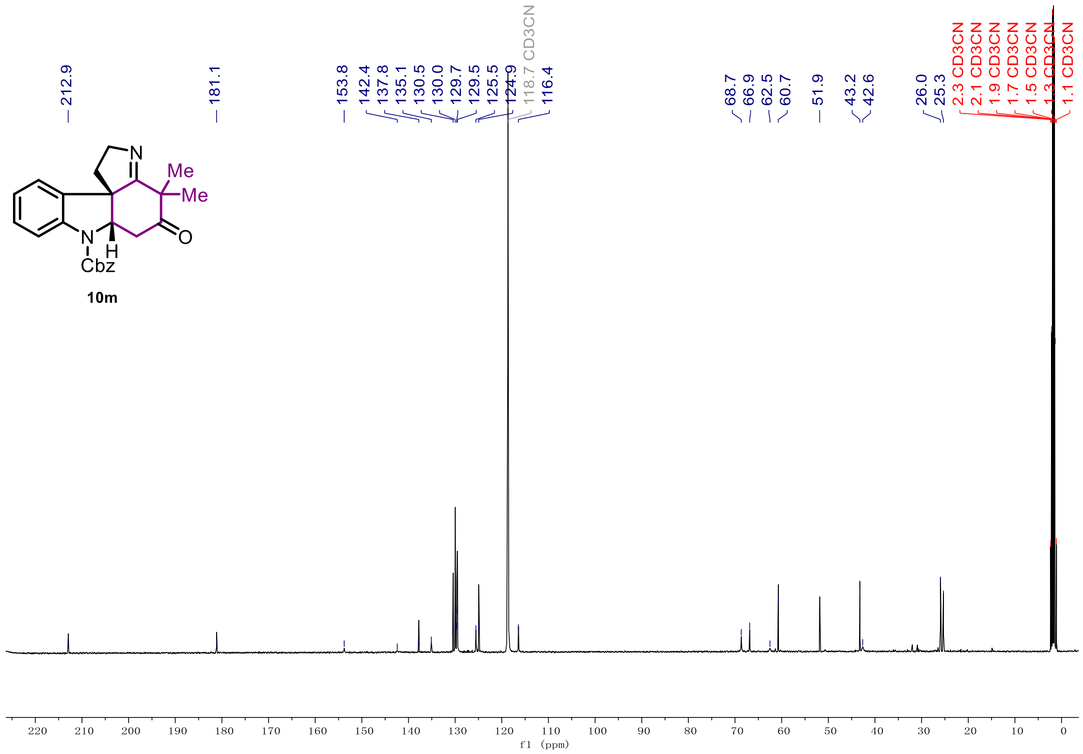

^{13}C NMR spectra of **10m** (101 MHz, Acetonitrile-d_3)

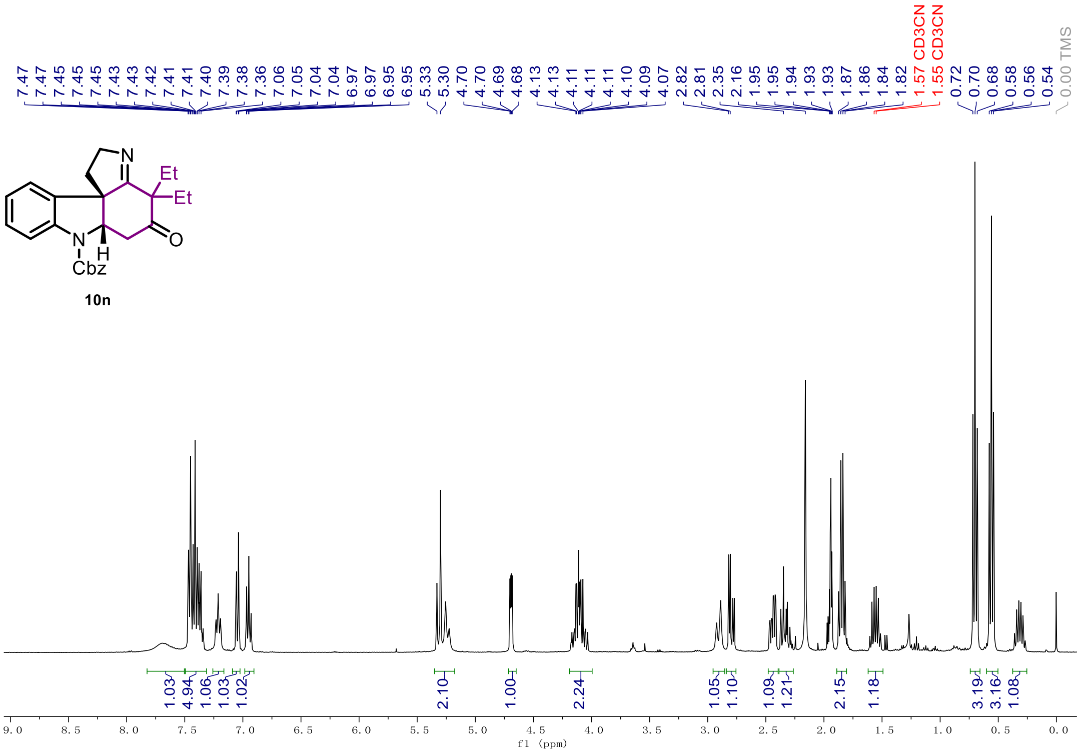

^{1}H NMR spectra of **10n** (400 MHz, Acetonitrile-d_3)

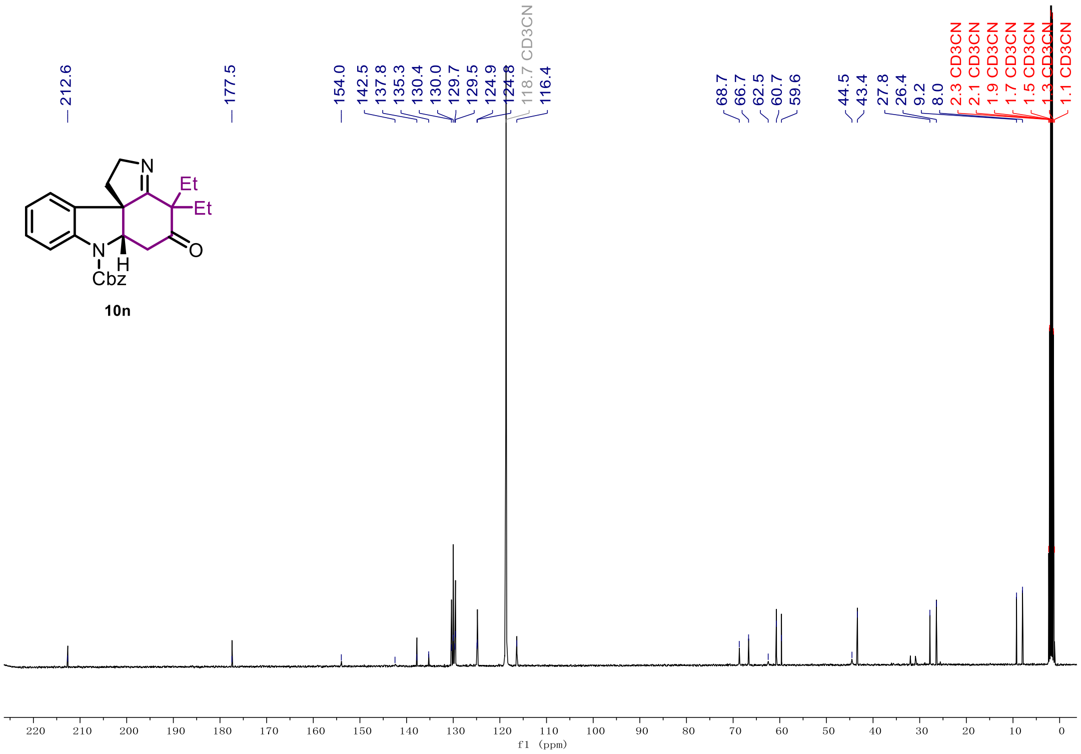

^{13}C NMR spectra of **10n** (101 MHz, Acetonitrile-d_3)

IV. References

[1] (a) Mizuno, K.; Sawa, M.; Harada, H.; Taoka, I.; Yamashita, H.; Oue, M.; Tsujiuchi, H.; Arai, Y.; Suzuki, S.; Furutani, Y.; Kato, S. *Bioorg. Med. Chem.* **2005**, *13*, 855-868. (b) Sato, S.; Hirayama, A.; Adachi, T.; Kawauchi, D.; Ueda, H.; Tokuyama, H. *Heterocycles*, **2017**, *94*, 1940-1957. (c) Furst, L.; Narayanam, J. M. R.; Stephenson, C. R. J. *Angew. Chem., Int. Ed.* **2011**, *50*, 9655–9659

[2] Brand, S.; de Candole, B. C.; Brown, J. A. *Org. Lett.* **2003**, *5*, 2343-2346.

[3] Long, J.-Y.; Liu, R.-D.; Mu, X.-P; Song, Z.-L.; Zhang, Z.-C.; Yang, Z. *Org. Lett.* **2024**, *26*, 2960−2964.

[4] (a) Li, J.; Han, Y. B.; Freedman, T. B.; Zhu, S. F.; Kerwood, D. J.; Luk, Y. -Y. *Tetrahedron Lett.* **2008**, *49*, 2128-2131. (b) White, J. D.; Li, Y.; Ihle, D. C. *J. Org. Chem.* **2010**, *75*, 3569-3577.

[5] Frisch, M. J., Trucks, G. W., Schlegel, H. B., Scuseria, G. E., Robb, M. A., Cheeseman, J. R., Scalmani, G., Barone, V., Mennucci, B., Petersson, G. A., Nakatsuji, H., Caricato, M., Li, X., Hratchian, H. P., Izmaylov, A. F., Bloino, J., Zheng, G., Sonnenberg, J. L., Hada, M., Ehara, M., Toyota, K., Fukuda, R., Hasegawa, J., Ishida, M., Nakajima, T., Honda, Y., Kitao, O., Nakai, H., Vreven, T., Montgomery, J. A., Peralta, J. E., Ogliaro, F., Bearpark, M., Heyd, J. J., Brothers, E., Kudin, K. N., Staroverov, V. N., Kobayashi, R., Normand, J., Raghavachari, K., Rendell, A., Burant, J. C., Iyengar, S. S., Tomasi, J., Cossi, M., Rega, N., Millam, J. M., Klene, M., Knox, J. E., Cross, J. B., Bakken, V., Adamo, C., Jaramillo, J., Gomperts, R., Stratmann, R. E., Yazyev, O., Austin, A. J., Cammi, R., Pomelli, C., Ochterski, J. W., Martin, R. L., Morokuma, K., Zakrzewski, V. G., Voth, G. A., Salvador, P., Dannenberg, J. J., Dapprich, S., Daniels, A. D., Farkas, O., Foresman, J. B., Ortiz, J. V., Cioslowski, J., Fox, D. J. *Gaussian 16, Revision C.01*. Gaussian, Inc., Wallingford CT, 2019.

[6] Stephens, P. J., Devlin, F. J., Chabalowski, C. F., Frisch, M. J. *J. Phys. Chem.* **1994**, *98*, 11623.

[7] Hariharan, P. C., Pople, J. A. *Theor. Chim. Acta.* **1973**, *28*, 213.

[8] Hay, P. J., Wadt, W. R. *J. Chem. Phys.* **1985**, *82*, 299.

[9] Wadt, W. R., Hay, P. J. *J. Chem. Phys.* **1985**, *82*, 284.

[10] Hehre, W. J., Radom, L., Schleyer, P. R., Pople, J. A. *Ab Initio Molecular Orbital Theory*, Wiley, New York, **1986**.

[11] Weigend, F., Ahlrichs, R. *Phys. Chem. Chem. Phys.* **2005**, *7*, 3297.

[12] Grimme, S., Antony, J., Ehrlich, S., Krieg, H. *J. Chem. Phys.* **2010**, *132*, 154104.

[13] Marenich, A. V., Cramer, C. J., Truhlar, D. G. *J. Phys. Chem. B* **2009**, *113*, 6378.

[14] Fukui, K. *J. Phys. Chem.* **1970**, *74*, 4161.

[15] Fukui, K. *Acc. Chem. Res.*, **1981**, *14*, 363.

[16] Legault, C. Y. *CYLview*, 1.0b; Universite de Sherbrooke, 2009. http://www.cylview.org.

[17] Humphrey, W., Dalke, A., Schulten, K. *J. Mol. Graphics* **1996**, *14*, 33.
